# Tissue MicroRNA profiles as diagnostic and prognostic biomarkers in patients with resectable pancreatic ductal adenocarcinoma and periampullary cancers

**DOI:** 10.1186/s40364-017-0087-6

**Published:** 2017-02-21

**Authors:** Dan Calatayud, Christian Dehlendorff, Mogens K. Boisen, Jane Preuss Hasselby, Nicolai Aagaard Schultz, Jens Werner, Heike Immervoll, Anders Molven, Carsten Palnæs Hansen, Julia S. Johansen

**Affiliations:** 1 Department of Surgical Gastroenterology and Transplantation, Rigshospitalet, Copenhagen University Hospital, Copenhagen, Denmark; 2 Danish Cancer Society Research Center, Danish Cancer Society, Copenhagen, Denmark; 3 Department of Oncology, Herlev and Gentofte Hospital, Copenhagen University Hospital, Herlev, Denmark; 4 Department of Pathology, Rigshospitalet, Copenhagen University Hospital, Copenhagen, Denmark; 5 Department of General, Visceral, and Transplant Surgery, LMU, University of Munich, Munich, Germany; 6 Gade Laboratory for Pathology, Department of Clinical Medicine, University of Bergen, Bergen, Norway; 7 Department of Pathology, Ålesund Hospital, Ålesund, Norway; 8 Department of Pathology, Haukeland University Hospital, Bergen, Norway; 9 Department of Medicine, Herlev and Gentofte Hospital, Copenhagen University Hospital, Herlev, Denmark; 10 Institute of Clinical Medicine, Faculty of Health and Medical Sciences, University of Copenhagen, Copenhagen, Denmark; 11 Department of Oncology, Herlev University Hospital, Herlev Ringvej 75, DK-2730 Herlev, Denmark

**Keywords:** Ampullary cancer, Biomarkers, microRNA, Pancreatic ductal adenocarcinoma, Pancreatic cancer

## Abstract

**Background:**

The aim of this study was to validate previously described diagnostic and prognostic microRNA expression profiles in tissue samples from patients with pancreatic cancer and other periampullary cancers.

**Methods:**

Expression of 46 selected microRNAs was studied in formalin-fixed paraffin-embedded tissue from patients with resected pancreatic ductal adenocarcinoma (*n* = 165), ampullary cancer (*n*=59), duodenal cancer (*n* = 6), distal common bile duct cancer (*n* = 21), and gastric cancer (*n* = 20); chronic pancreatitis (*n* = 39); and normal pancreas (*n* = 35). The microRNAs were analyzed by PCR using the Fluidigm platform.

**Results:**

Twenty-two microRNAs were significantly differently expressed in patients with pancreatic cancer when compared to healthy controls and chronic pancreatitis patients; 17 miRNAs were upregulated (miR-21-5p, −23a-3p, −31-5p, −34c-5p, −93-3p, −135b-3p, −155-5p, −186-5p, −196b-5p, −203, −205-5p, −210, −222-3p, −451, −492, −614, and miR-622) and 5 were downregulated (miR-122-5p, −130b-3p, −216b, −217, and miR-375). MicroRNAs were grouped into diagnostic indices of varying complexity. Ten microRNAs associated with prognosis were identified (let-7 g, miR-29a-5p, −34a-5p, −125a-3p, −146a-5p, −187, −205-5p, −212-3p, −222-5p, and miR-450b-5p). Prognostic indices based on differences in expression of 2 different microRNAs were constructed for pancreatic and ampullary cancer combined and separately (30, 5, and 21 indices).

**Conclusion:**

The study confirms that pancreatic cancer tissue has a microRNA expression profile that is different from that of other periampullary cancers, chronic pancreatitis, and normal pancreas. We identified prognostic microRNAs and microRNA indices that were associated with shorter overall survival in patients with radically resected pancreatic cancer.

**Electronic supplementary material:**

The online version of this article (doi:10.1186/s40364-017-0087-6) contains supplementary material, which is available to authorized users.

## Background

Pancreatic cancer (PC) is the fourth most common cause of cancer-related death in the Western world, although it only represents 3% of all new cancer cases [1, 2]. Most cases are pancreatic ductal adenocarcinomas (PDAC). Due to locally advanced or metastatic disease, only 20% of all patients diagnosed with PC are accessible to radical surgical treatment, and thereby have the potential for long-term survival [3, 4]. However, even in this group, the 5-year survival is only 20% due to the high recurrence rate [5, 6].

PC located in the head of the pancreas constitutes the majority (60–70%) of the group of cancers in the region, which also includes of ampullary adenocarcinomas (A-AC), accounting for 15–25%; and duodenal cancers (DC); and distal common bile duct (CBD) cancers, each accounting for approximately 10%[6]. The distribution of the different types of the periampullary cancers is variously reported, probably due to the complexity of the periampullary anatomy and histopathology. The 5-year survival rate after surgery is 45–55% for A-AC and DC [7, 8] and approximately 25% for distal CBD cancers [6].

Cancer antigen 19–9 (CA 19–9, also named carbohydrate antigen 19–9 and sialylated Lewis antigen) is the most widely used biomarker for patients with PC. Serum CA19-9 alone is insufficient as a diagnostic biomarker, although it may have prognostic value in the absence of cholestasis [9]. There is an obvious need for better biomarkers in PC, and microRNAs (miRNAs, miRs) could be interesting in this regard.

MiRNAs are small (18–24 nucleotides) non-coding RNAs that regulate gene expression post-transcriptionally by binding to messenger RNA molecules through nucleotide complementarity [10, 11]. MiRNAs regulate critical cellular processes such as differentiation, proliferation, apoptosis, and metastasis [12–16]. MiRNAs are stable and analyzable in formalin-fixed paraffin-embedded (FFPE) tissue, which is suitable for analysis [17, 18]. So far, 2603human miRNA sequences have been discovered and the number is increasing [19].

The expression patterns of miRNAs can be combined into profiles that are specific for a given type of tissue or disease. Several specific miRNA expression profiles in PC tissue have been described, with a promising consistency between studies and different array or PCR platforms. The expressions of miR-15b, −21, −95, −103, −107, −122, −135b, −148a, −155, −190, −196a, −200, −203, −210, −216b, −217, −221, −222, and miR-375 differ between PC and normal pancreas or chronic pancreatitis [20–28]. Furthermore, miRNA expression profiling indicates a close relationship between PDAC and A-AC [27]. Specific miRNAs have also been suggested as prognostic biomarkers in several cancers, including PC [23, 29–32].

The aim of the present study was to validate previously described diagnostic and prognostic miRNA expression profiles for PDAC and A-AC in FFPE specimens.

## Methods

### Patients

#### Diagnostic miRNA study

FFPE tumor specimens (*n* = 359 including an internal control) were obtained from patients who underwent resection with radical intent for the following diagnoses: PDAC (*n* = 165), A-AC (*n* = 59), DC (*n* = 6), distal CBD cancer (*n* = 21), chronic pancreatitis (CP) (*n* = 39), gastric cancer (GC) (*n* = 20), serous cyst adenoma (*n* = 2), and no cancer (*n* = 4; cysts or fibrosis that could not be classified as normal pancreas or pancreatitis and did not have any malignant foci) and healthy subjects (HS) (*n* = 35). The pancreatic and periampullary specimens came from patients who had undergone pancreaticoduodenectomy, distal pancreatectomy, or total pancreatectomy between 2004 and 2011 in Denmark (Herlev Hospital *n* = 9; Rigshospitalet *n* = 198), Germany (Heidelberg *n* = 69), and Norway (Bergen *n* = 55). The chronic pancreatitis specimens came from Copenhagen (*n* = 5) and Heidelberg (*n* = 34). All normal pancreas tissue was obtained from Heidelberg from organ donors or patients with traumatic pancreatic lesions leading to resection of healthy pancreatic tissue. The Danish patients were included in the BIOPAC Study (BIOmarkers in patients with Pancreatic Cancer). The gastric cancers came from patients who had undergone surgery at Gentofte Hospital. An experienced pathologist reassessed all samples to select the most representative part of the specimen, and tumors were classified and graded according to the World Health Organization criteria [33].

#### Prognostic miRNA study

One hundred fifty-seven FFPE tumor specimens were analyzed from patients who underwent surgery with radical intent for PDAC (*n* = 103) and A-AC (*n* = 54). The patients were included in the BIOPAC Study at Rigshospitalet in Denmark. Inclusion criteria were age ≥ 18 years and histologically verified PC in a resected specimen. After surgery, the majority of the patients (87%) were treated with adjuvant gemcitabine for 6 months or until disease recurrence.

Patient characteristics are shown in Table 1.

**Table 1:** Characteristics of the Danish patients

Characteristic	PDAC N = 110	A-AC N = 59	Duodenal cancer N = 6	Distal CBD cancer N = 21	Chronic pancreatitis N = 5	Serous cystadenoma and other benign diagnosis N = 6
Age, years median (range)	65.7 (37.4-81.3)	64.9 (38.3-80.5)	69.0 (54.3-74.4)	64.7 (38.6-74.6)	56.4 (43.8-68.2)	60.6 (46.7-84.7)
Gender						
Male	60 (55%)	37 (63%)	5 (83%)	11 (52%)	5 (100%)	2 (33%)
Female	50 (45%)	22 (37%)	1 (17%)	10 (48%)	0	4 (67%)
ASA score						
1	12 (11%)	9 (15%)	0	2 (10%)	1 (20%)	0
2	58 (53%)	38 (66%)	5 (83%)	15 (75%)	2 (40%)	4 (80%)
3	30 (27%)	11 (19%)	1 (17%)	3 (15%)	2 (40%)	1 (20%)
4	0	0		0	0	0
TNM-Stage						
IA	9 (8%)	4 (7%)	1 (17%)	1 (5%)		
IB	3 (3%)	7 (12%)	1 (17%)	1 (5%)		
IIA	27 (25%)	6 (10%)	2 (33%)	7 (52%)		
IIB	67 (65%)	24 (41%)	2 (33%)	11 (33%)		
III	0	16 (27%)	0	1 (5%)		

Values are N (%). Numbers may not add up due to missing values

No clinical information is available from the patients with gastric cancer and the patients and healthy subjects from Heidelberg and Bergen

### MiRNA purification from FFPE tissues

One FFPE block was selected from each patient for miRNA analysis. From each of these blocks, 3 10-μm sections were cut for miRNA extraction without micro-dissection. As method control, 9×3 sections were cut from a specimen from 1 of the PDAC patients. MiRNAs were extracted using Qiagen miRNeasy FFPE kit, Cat No./ID: 217504. Briefly, the sections were deparaffinized in xylene and ethanol and then treated with proteinase K, and RNA was isolated using the one-column spin column protocol for total RNA. The concentration of small RNAs was assessed by absorbance spectrometry on a DTX 880 (Beckman Coulter).

### MiRNA analysis

The following 46 miRNAs were selected for analysis: miR-21-5p, −23a-3p, −29a-5p, −31-5p, −34a-5p, −34c-5p, −93-3p, −122-5p, −125a-3p, −130b-3p, −135b-3p, −136-3p, −146a-5p, −148a-3p, −148a-5p, −155-5p, −186-5p, −187-3p, −194-3p, −196b-5p, −198, −203, −205-5p, −210, −212-3p, −216b, −217, −222-3p, −222-5p, −375, −411-5p, −431-5p, −450b-5p, −451a, −490-3p, −492, −509-5p, −571, −614, −622, −625-5p, −675-5p, −769-5p, −939, −944, and let-7 g. The selection was based on the previously described relationship of the miRNAs to PC in particular and to cancer biology in general (Detailed information on each specific miRNA is available in “Additional file 1”).

The miRNAs were analyzed in triplicate using the Fluidigm BioMark System™. This system can perform multiple simultaneous real-time PCR measurements running gold-standard Taqman® assays in nanolitre quantities. The instructions from Fluidigm were followed in all details (https://www.fluidigm.com). The analyses were performed at AROS Applied Biotechnology A/S (www.arosab.com, Aarhus, Denmark).

### Statistical analysis

Differences in miRNA expression according to diagnosis were tested by univariate logistic regression including the raw miRNA expression level as continuous variables on the cycle threshold scale. Odds ratios (OR) per inter-quartile increase and 95% confidence intervals were computed for both PC vs. HS and PC vs. HS and CP.

Diagnostic indices were identified in 3 different ways among the significant miRNAs: (1) As a manually defined index by including 2 miRNA with OR > 1 and 2 with OR < 1 (indices I and IV);(2) As a computer generated index found by backwards elimination of a model with miRNAs chosen from 18 miRNAs described in an previous index (the so-called LASSO-classifier: miR-23a, 34c-5p, −122, −135b-3p, −136-3p, −186, −196b, −198, −203, −222-3p, −451, −490, −492, −509-5p, −571, −614, −622, and miR-93 [27]) which were significant at a 1% significance level, to account for multiple testing and with less than 10% missing values (indices II and V) and (3) as a computer generated index like (2) but based on all significant miRNAs (indices III and VI). A total of 6 indices were identified: I, II, and III developed for the PC vs. HS comparison and IV, V, and VI developed for the PC vs. HS + CP comparison. The indices were evaluated by means of boxplots, and their performance was evaluated by computing sensitivity, specificity, accuracy, area under curve (AUC), true positives (TP), true negatives (TN), false positives (FP), and false negatives (FN). The indices were also tested on other cancer types. For each index, we first found a suitable cut-off by requiring a sensitivity of 85% in the PC vs. HS or vs. HS + CP comparison. Subsequently, this cut-off point was applied in all other comparisons.

It was not possible to stratify our patients according to TNM due to the very uneven distribution of cancer stages and resulting small subgroups.

For the prognostic study, the association between overall survival (OS) and miRNA expression was illustrated by Kaplan–Meier curves by dichotomizing the miRNA expression into below and above the median expression for each miRNA. The association was tested by means of univariate Cox proportional hazards regression both on the continuous variables and on the dichotomized variables, and presented as hazard ratios (HR) and corresponding 95% confidence intervals (CIs). In addition, analyses adjusted for age, sex, tumor stage, ASA score, and tumor differentiation were performed. Finally, we considered differences between 2 miRNAs at a time as a continuous variable in the Cox models (unadjusted and adjusted) for OS. Analyses were made for the diagnoses PDAC and A-AC together and separately.

In all analysis, the software package R version 3.1.1 (R Core Team 2014; R: A language and environment for statistical computing. R Foundation for Statistical Computing, Vienna, Austria. www.R-project.org) was used, and *P-*values below 5% were considered statistically significant.

## Results

### Diagnosis – Pancreatic cancer vs. healthy subjects

The following 14 miRNAs were upregulated in PC compared to HS: miR-21-5p, −23a-3p, −31-5p, −34c-5p, −93-3p, −135b-3p, −155-5p, −196b-5p, −203, −205-5p, −210, −222-3p, −451, and miR-622. The following 5 miRNAs were downregulated in PC: miR-122-5p, −130b-3p, −216b, − 217, and miR-375 (Table 2).

**Table 2:** Significantly deregulated microRNAs

microRNA upregulated in PC compared to healthy subjects					
miRNA	OR (CI)	*p*-value	PC	HS	Missing
miR-21-5p	0.11 (0.03–0.25)	0.0000	134	13	53
miR-23a-3p	0.36 (0.13–0.67)	0.0100	156	5	39
miR-31-5p	0.38 (0.28–0.50)	0.0000	165	35	0
miR-34c-5p	0.17 (0.09–0.28)	0.0000	165	35	0
miR-93-3p	0.14 (0.06–0.26)	0.0000	165	34	1
miR-135b-3p	0.31 (0.20–0.44)	0.0000	165	30	5
miR-155-5p	0.11 (0.03–0.23)	0.0000	165	33	2
miR-196b-5p	0.14 (0.02–0.45)	0.0151	147	3	50
miR-203	0.37 (0.25–0.51)	0.0000	165	35	0
miR-205-5p	0.71 (0.59–0.82)	0.0000	148	21	31
miR-210	0.12 (0.05–0.22)	0.0000	165	34	1
miR-222-3p	0.06 (0.02–0.15)	0.0000	165	35	0
miR-451	0.14 (0.06–0.27)	0.0000	165	35	0
miR-622	0.57 (0.41–0.76)	0.0003	165	34	1
microRNA downregulated in PC compared to healthy subjects					
miRNA	OR (CI)	*p*-value	PC	HS	Missing
miR-122-5p	2.08 (1.40–3.51)	0.0014	30	18	152
miR-130b-3p	5.34 (3.17–9.98)	0.0000	165	35	0
miR-216b	6.30 (3.36–14.24)	0.0000	149	35	16
miR-217	2.94 (2.03–4.69)	0.0000	142	35	23
miR-375	26.10 (9.48–90.22)	0.0000	165	35	0
microRNA upregulated in PC compared to healthy subjects and chronic pancreatitis					
miRNA	OR (CI)	*p*-value	PC	HS + CP	Missing
miR-21-5p	0.24 (0.14–0.36)	0.0000	134	42	63
miR-23a-3p	0.54 (0.38–0.74)	0.0003	156	31	52
miR-31-5p	0.50 (0.41–0.59)	0.0000	165	74	0
miR-34c-5p	0.33 (0.25–0.43)	0.0000	165	74	0
miR-93-3p	0.27 (0.17–0.40	0.0000	165	73	1
miR-135b-3p	0.31 (0.22–0.41	0.0000	165	58	16
miR-155-5p	0.46 (0.37–0.56	0.0000	165	72	2
miR-186-5p	0.71 (0.55–0.89	0.0041	165	74	0
miR-196b-5p	0.53 (0.39–0.70	0.0000	147	20	72
miR-203	0.36 (0.26–0.46	0.0000	165	74	0
miR-205-5p	0.79 (0.71–0.88	0.0000	148	46	45
miR-210	0.27 (0.18–0.36	0.0000	165	73	1
miR-222-3p	0.23 (0.16–0.32	0.0000	165	74	0
miR-451	0.44 (0.35–0.54	0.0000	165	74	0
miR-492	0.46 (0.22–0.78	0.0097	57	4	178
miR-614	0.75 (0.57–0.94	0.0219	110	14	115
miR-622	0.52 (0.41–0.66	0.0000	165	72	2
microRNA downregulated in PC compared to healthy subjects and chronic pancreatitis					
miRNA	OR (CI)	*p*-value	PC	HS + CP	Missing
miR-122-5p	1.99 (1.46–2.98)	0.0001	30	40	169
miR-130b-3p	1.71 (1.33–2.23)	0.0001	165	74	0
miR-216b	1.55 (1.34–1.84)	0.0000	149	73	17
miR-217	1.46 (1.28–1.69)	0.0000	142	71	26
miR-375	2.22 (1.62–3.15)	0.0000	165	74	0

Three indices of miRNA expression, index I, II, and III, were identified to separate PC from HS (i.e., normal pancreas tissue):

(I) A manually defined index: miR-375 + miR-130b-3p – miR-451 – miR34c-5p.

(II) A computer-generated index based on univariate significant miRNAs chosen from 18 miRNAs describes in a previous index with less than 10% missing: 292.6458–3.0539×miR-34c-5p + 4.007×miR-203–10.4×miR-222-3p–3.6057×miR-451–4.3015×miR-622.

The potential miRNAs for index II were miR-34c-5p, −135-3p, −203, −222-3p, −451,and miR-622.

(III) A computer-generated index based on all univariate significant miRNAs with less than 10% missing values: 118.7249 + 77.2459×miR-130b-3p–23.7911×miR-34c-5p–49.923×miR-451.

The potential miRNAs for index III were miR-31-5p, −34c-5p,-93-3p, −130b-3p, −135b-3p, −155-5p, −203, −205-5p, −210, −216b, −217, −222-3p, −375, −451,and miR-622.

**Fig. 1:**
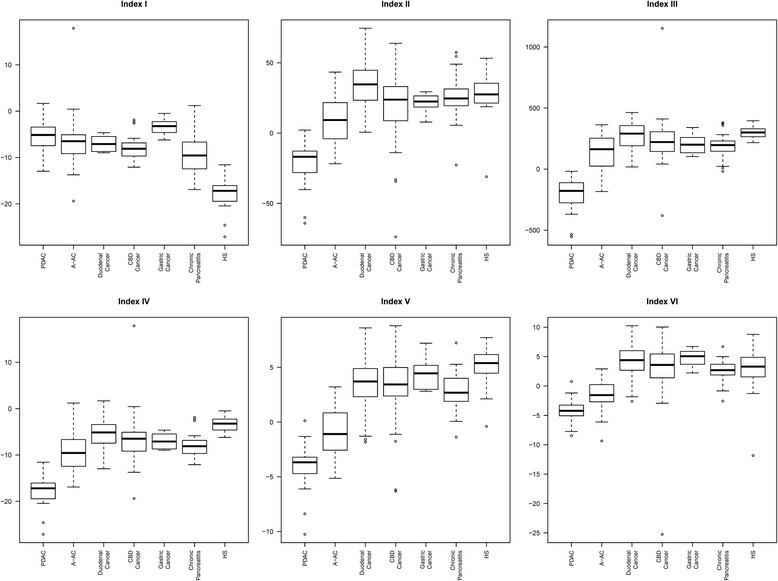
Performance of diagnostic indices for PC vs. HS and for PC vs. HS + CP

The performances of these indices are illustrated in box plots in Fig. 1 and Table 3 (upper part). The manually calculated index I was able to separate PC from HS with a sensitivity of 84.9 (CI 78.5–90.0), but could also differentiate the other malignant diagnoses from HS, with a sensitivity varying from 66.7 (distal CBD cancer) to 100.0 (DC and GC). The computer-generated index II performed in the same way with regard to PC vs. HS, but was inferior for separating the other malignancies from HS except for distal CBD cancer, where it performed better than index I. The computer-generated index III performed slightly better than index II with regard to separating A-AC and DC cancer from HS, but was inferior for separating distal CBD cancer and GC.

**Table 3:** Performance of diagnostic indices

Study	Index	Designed sensitivity	cutoff	Sensitivity (CI)	Specificity (CI)	Accuracy (CI)	AUC (CI)	TP	TN	FP	FN
Performance of diagnostic indices developed on PC vs. HS											
PC vs. HS	I	0.85	−9.13	84.85 (78.45–89.95)	100.00 (90.00–100.00)	87.50 (82.10–91.74)	1.00 (1.00–1.00)	140	35	0	25
A-AC vs. HS	I		−9.13	74.58 (61.56–85.02)	100.00 (90.00–100.00)	84.04 (75.05–90.78)	0.99 (0.96–1.00)	44	35	0	15
DC vs. HS	I		−9.13	100.00 (54.07–100.00)	100.00 (90.00–100.00)	100.00 (91.40–100.00)	1.00 (1.00–1.00)	6	35	0	0
CBD vs. HS	I		−9.13	66.67 (43.03–85.41)	100.00 (90.00–100.00)	87.50 (75.93–94.82)	1.00 (0.99–1.00)	14	35	0	7
A-AC, DC, CBD vs. HS	I		−9.13	74.42 (63.87–83.22)	100.00 (90.00–100.00)	81.82 (73.78–88.24)	0.99 (0.97–1.00)	64	35	0	22
GC vs. HS	I		−9.13	100.00 (83.16–100.00)	100.00 (90.00–100.00)	100.00 (93.51–100.00)	1.00 (1.00–1.00)	20	35	0	0
PC vs. HS	II	0.85	16.68	84.85 (78.45–89.95)	100.00 (90.00–100.00)	87.50 (82.10–91.74)	1.00 (1.00–1.00)	140	35	0	25
A-AC vs. HS	II		16.68	67.80 (54.36–79.38)	100.00 (90.00–100.00)	79.79 (70.25–87.37)	0.94 (0.89–0.98)	40	35	0	19
DC vs. HS	II		16.68	83.33 (35.88–99.58)	100.00 (90.00–100.00)	97.56 (87.14–99.94)	1.00 (1.00–1.00)	5	35	0	1
CBD vs. HS	II		16.68	80.95 (58.09–94.55)	100.00 (90.00–100.00)	92.86 (82.71–98.02)	0.97 (0.90–1.00)	17	35	0	4
A-AC, DC, CBD vs. HS	II		16.68	72.09 (61.38–81.23)	100.00 (90.00–100.00)	80.17 (71.94–86.86)	0.95 (0.91–0.99)	62	35	0	24
GC vs. HS	II		16.68	95.00 (75.13–99.87)	100.00 (90.00–100.00)	98.18 (90.28–99.95)	0.96 (0.87–1.00)	19	35	0	1
PC vs. HS	III	0.85	149.10	84.85 (78.45–89.95)	100.00 (90.00–100.00)	87.50 (82.10–91.74)	1.00 (1.00–1.00)	140	35	0	25
A-AC vs. HS	III		149.10	72.88 (59.73–83.64)	100.00 (90.00–100.00)	82.98 (73.84–89.95)	0.98 (0.95–1.00)	43	35	0	16
DC vs. HS	III		149.10	66.67 (22.28–95.67)	100.00 (90.00–100.00)	95.12 (83.47–99.40)	1.00 (1.00–1.00)	4	35	0	2
CBD vs. HS	III		149.10	71.43 (47.82–88.72)	100.00 (90.00–100.00)	89.29 (78.12–95.97)	1.00 (0.99–1.00)	15	35	0	6
A-AC, DC, CBD vs. HS	III		149.10	72.09 (61.38–81.23)	100.00 (90.00–100.00)	80.17 (71.94–86.86)	0.99 (0.97–1.00)	62	35	0	24
GC vs. HS	III		149.10	100.00 (83.16–100.00)	100.00 (90.00–100.00)	100.00 (93.51–100.00)	1.00 (1.00–1.00)	20	35	0	0
Performance of diagnostic indices developed on PC vs. HS + CP											
PC vs. HS + CP	IV	0.85	−9.13	84.85 (78.45–89.95)	75.68 (64.31–84.90)	82.01 (76.54–86.66)	0.89 (0.84–0.94)	140	56	18	25
A-AC vs. HS + CP	IV		−9.13	74.58 (61.56–85.02)	75.68 (64.31–84.90)	75.19 (66.96–82.26)	0.83 (0.76–0.90)	44	56	18	15
DC vs. HS + CP	IV		−9.13	100.00 (54.07–100.00)	75.68 (64.31–84.90)	77.50 (66.79–86.09)	0.85 (0.76–0.93)	6	56	18	0
4 vs. HS + CP	IV		−9.13	66.67 (43.03–85.41)	75.68 (64.31–84.90)	73.68 (63.65–82.19)	0.80 (0.71–0.88)	14	56	18	7
A-AC, DC, CBD vs. HS + CP	IV		−9.13	74.42 (63.87–83.22)	75.68 (64.31–84.90)	75.00 (67.55–81.50)	0.83 (0.76–0.89)	64	56	18	22
CG vs. HS + CP	IV		−9.13	100.00 (83.16–100.00)	75.68 (64.31–84.90)	80.85 (71.44–88.24)	0.97 (0.93–1.00)	20	56	18	0
PC vs. HS + CP	V	0.85	1.38	84.85 (78.45–89.95)	91.89 (83.18–96.97)	87.03 (82.10–91.01)	0.96 (0.94–0.98)	140	68	6	25
A-AC vs. HS + CP	V		1.38	77.97 (65.27–87.71)	91.89 (83.18–96.97)	85.71 (78.59–91.17)	0.93 (0.87–0.97)	46	68	6	13
DC vs. HS + CP	V		1.38	100.00 (54.07–100.00)	91.89 (83.18–96.97)	92.50 (84.39–97.20)	1.00 (0.98–1.00)	6	68	6	0
CBD vs. HS + CP	V		1.38	85.71 (63.66–96.95)	91.89 (83.18–96.97)	90.53 (82.78–95.58)	0.94 (0.89–0.98)	18	68	6	3
A-AC, DC, CBD vs. HS + CP	V		1.38	81.40 (71.55–88.98)	91.89 (83.18–96.97)	86.25 (79.93–91.18)	0.94 (0.89–0.97)	70	68	6	16
GC vs. HS + CP	V		1.38	95.00 (75.13–99.87)	91.89 (83.18–96.97)	92.55 (85.26–96.95)	0.99 (0.96–1.00)	19	68	6	1
PC vs. HS + CP	VI	0.85	1.46	84.85 (78.45–89.95)	93.24 (84.93–97.77)	87.45 (82.57–91.37)	0.97 (0.95–0.99)	140	69	5	25
A-AC vs. HS + CP	VI		1.46	72.88 (59.73–83.64)	93.24 (84.93–97.77)	84.21 (76.88–89.95)	0.92 (0.87–0.96)	43	69	5	16
DC vs. HS + CP	VI		1.46	100.00 (54.07–100.00)	93.24 (84.93–97.77)	93.75 (86.01–97.94)	0.99 (0.97–1.00)	6	69	5	0
CBD vs. HS + CP	VI		1.46	76.19 (52.83–91.78)	93.24 (84.93–97.77)	89.47 (81.49–94.84)	0.93 (0.87–0.98)	16	69	5	5
A-AC, DC, CBD vs. HS + CP	VI		1.46	75.58 (65.13–84.20)	93.24 (84.93–97.77)	83.75 (77.10–89.10)	0.93 (0.89–0.96)	65	69	5	21
GC vs. HS + CP	VI		1.46	75.00 (50.90–91.34)	93.24 (84.93–97.77)	89.36 (81.30–94.78)	0.91 (0.80–0.98)	15	69	5	5

*AUC* Area under Curve, *TP* True positive, *TN* True negative, *FP* False positive, *FN* False negative, *PC* Pancreatic Cancer, *A-AC* Ampullary Adenocarcinoma, *DC* Duodenal Cancer, *CBD* Common bile duct cancer, *GC* Gastric cancer, *HS* Healthy subjects

### Diagnosis - Pancreatic cancer vs. healthy subjects + chronic pancreatitis

The following 17 miRNAs were upregulated in PDAC compared with benign specimens (HS and CP combined): miR-21-5p, −23a-3p, −31-5p, −34c-5p, −93-3p, −135b-3p, −155-5p, −186-5p, −196b-5p, −203, −205-5p, −210, −222-3p, −451, −492, −614, and miR-622. The following 5 miRNAs were downregulated in PDAC compared to benign specimens (HS and CP combined): miR-122-5p, −130b-3p, −216b, −217, and miR-375 (Table 2).

Three indices, IV, V, and VI, of miRNA expression to separate PC from benign tissue (i.e., HS and CP combined) were identified.

(IV) A manually defined index: miR-375 + miR-130b-3p – miR-451 – miR-34c-5p.

(V) A computer-generated index based on significant miRNAs chosen from 18 miRNAs described in a previous index with less than 10% missing values: 20.5487–1.5899×miR-222-3p–0.4006×miR-451–0.3864×miR-203–0.5056×miR-622+ 1.203×miR-186-5p.

The potential miRNAs for index V weremiR-34c-5p, −135b-3p, −186-5p, −203, −222-3p, −451, and miR-622.

(VI) A computer-generated index based on all significant miRNAs with less than 10% missing values: 7.1834–0.5175×miR-210 + 1.3893×miR-93-3p – 0.7423×miR-375–2.6184×miR-222-3p – 0.3414×miR-451–0.3852×miR-203–0.5316×miR-622 + 1.822×miR-186-5p.

The potential miRNAs for index VI were miR-31-5p, −34c-5p, −93-3p, −130b-3p, −135b-3p, −155-5p, −186-5p, −203, −210, −216b, −217, −222-3p, −375, −451, and miR-622.

The performances of these indices are illustrated in box plots in Fig. 1 and in Table 3 (lower part). Index IV could separate HS from the other diagnoses. Indices V and VI were able to separate CP from the malignant diagnoses.

### Diagnostic miRNA indices previously identified for pancreatic cancer

**Fig. 2:**
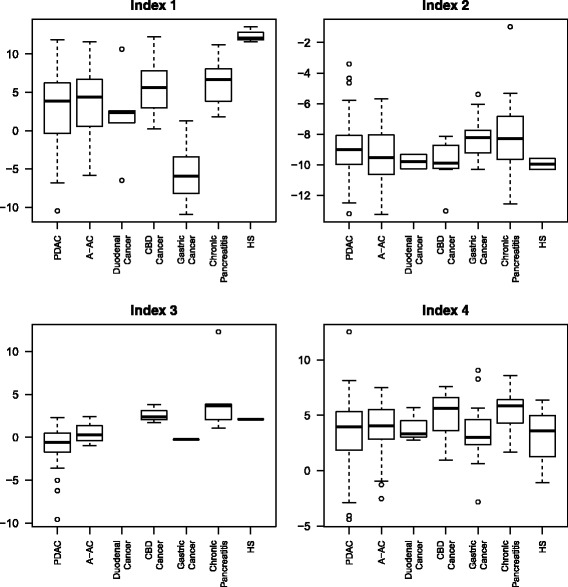
Performance of two miRNA diagnostic indices

We have previously described the following 4 different diagnostic miRNA indices in FFPE cancer tissues consisting of 2 different miRNAs [27]: (1) miR-196b-5p – miR-217; (2) miR-411 – miR-198; (3) miR-614 – miR-122-5p; and (4) miR-614 – miR-93-3p. The performance of the 4 indices in the present cohort was tested using the Fluidigm method. Since many samples had non-detectable miRNAs, we only used observations that were non-missing, i.e., not imputed by a large C_t_-value. Index 1 had 97 samples with at least 1miRNA missing, index 2 had 122 samples with at least 1 miRNA missing, index 3 had 213 samples with at least 1 miRNA missing, and index 4 had 115 samples with at least 1miRNA missing. For indices 2 and 3, it was not possible to consider HS alone. The performances of these indices are shown in box plots in Fig. 2. Index 1 could separate HS from PC patients but could not separate CP from A-AC. Index 1 could separate GC from all other diagnoses with high accuracy. Indices 2, 3, and 4 could not separate samples with benign from malignant diagnoses. Further information is given in the “Additional file 2”.

### Prognostic miRNAs – PDAC and A-AC patients combined

**Fig. 3:**
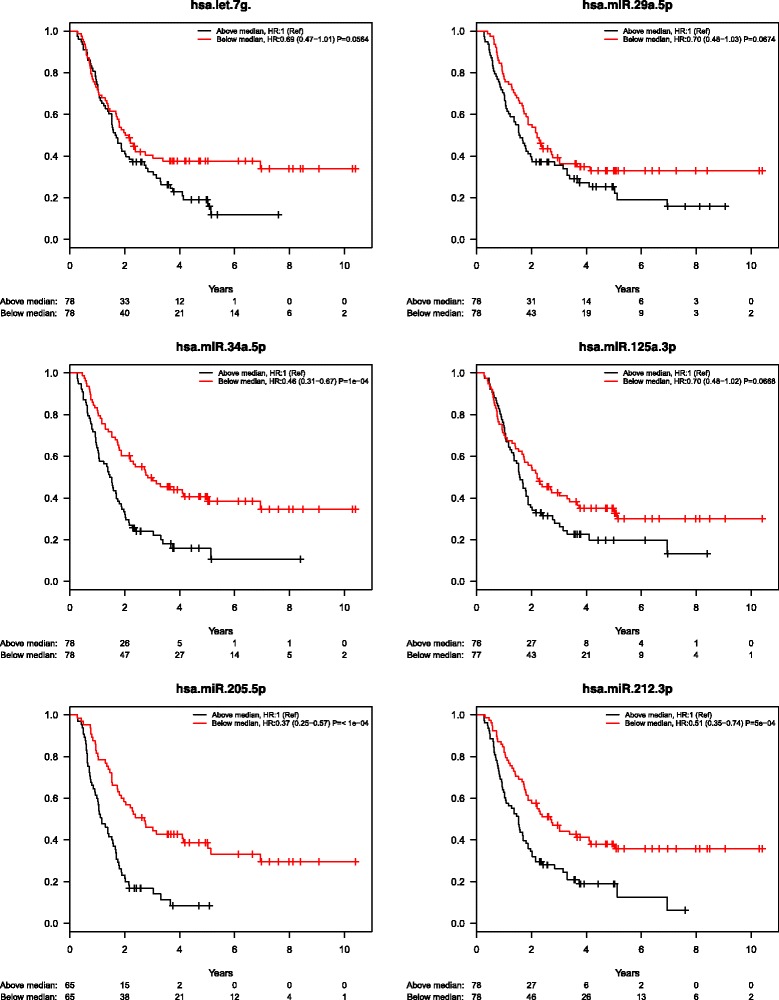
Kaplan–Meier curves for miRNAs significantly associated to survival in patients with PC + A-AC

In all, 157 patients with either PDAC or A-AC were available for the survival analysis, and 112died during the follow-up period. Table 4 illustrates that low expression of 6 miRNAs (miR-29a-5p, miR-34a-5p, miR-125a-3p, miR-146a-5p, miR-205-5p, and miR-212-3p) was associated with short OS, both with and without adjustment for age, sex, tumor stage/differentiation, and ASA-score. When patients were divided into 2 groups for each miRNA (defined as expression under or above the median level), low miR-34a-5p, miR-205-5p, miR-212-3p, and miR-222-5plevels were significantly associated with short OS. After adjusting for age, sex, tumor stage/differentiation, and ASA-score, let-7 g, miR-29a-5p, miR-34a-5p, miR-205-5p, and miR-212-3p were associated with short OS. Figure 3 illustrates Kaplan–Meier curves for the6 miRNAs reaching a significance level below 0.01.

**Table 4:** Prognostic miRNAs in patients with PC + A-AC, PC and A-AC

PDAC and A-AC							
CT-expression (per IQR increase)							
	Unadjusted			Adjusted			
miRNA	HR (CI)	P	N	HR (CI)		P	N
miR-29a-5p	0.87 (0.76–0.99)	0.0302	156	0.85 (0.74–0.98)		0.0212	145
miR-34a-5p	0.66 (0.54–0.81)	<0.0001	156	0.64 (0.52–0.79)		<0.0001	145
miR-125a-3p	0.83 (0.73–0.95)	0.0051	153	0.83 (0.72–0.95)		0.0077	142
miR-146a-5p	0.87 (0.76–0.99)	0.0296	157	0.85 (0.74–0.97)		0.0191	146
miR-205-5p	0.91 (0.86–0.96)	4e-04	130	0.92 (0.87–0.97)		0.0037	120
miR-212-3p	0.81 (0.72–0.91)	4e-04	156	0.80 (0.71–0.91)		4e-04	145
Under median vs. over median							
	Unadjusted			Adjusted			
miRNA	HR (CI)	P	N	HR (CI)		P	N
let-7 g	NS			0.62 (0.41–0.93)		0.0220	145
miR-29a-5p	NS			0.64 (0.42–0.96)		0.0314	145
miR-34a-5p	0.46 (0.31–0.67)	<0.0001	156	0.47 (0.31–0.71)		0.0003	145
miR-205-5p	0.37 (0.25–0.57)	<0.0001	130	0.44 (0.28–0.69)		0.0003	120
miR-212-3p	0.51 (0.35–0.74)	5e-04	156	0.53 (0.35–0.79)		0.0021	145
miR-222-5p	0.68 (0.47–1.00)	0.0495	152	NS			
PDAC							
CT-expression (per IQR increase)							
	Unadjusted			Adjusted			
miRNA	HR (CI)	P	N	HR (CI)		P	N
miR-34a-5p	0.72 (0.56–0.93)	0.0104	103	0.70 (0.52–0.93)		0.0144	93
miR-212-3p	0.83 (0.71–0.99)	0.0328	103	0.82 (0.68–0.99)		0.0350	93
Under median vs. over median							
	Unadjusted			Adjusted			
miRNA	HR (CI)	P	N	HR	(CI)	P	N
miR-34a-5p	0.49 (0.31–0.77)	0.0020	103	0.53 (0.32–0.89)		0.0151	93
miR-212-3p	0.64 (0.41–0.98)	0.0417	103	0.59 (0.36–0.97)		0.0358	93
A-AC							
CT-expression (per IQR increase)							
	Unadjusted			Adjusted			
miRNA	HR (CI)	P	N	HR (CI)		P	N
let-7 g	0.74 (0.58–0.93)	0.0100	53	NS			
miR-34a-5p	0.66 (0.46–0.94)	0.0218	53	0.58 (0.38–0.89)		0.0121	52
miR-187	1.51 (1.01–2.24)	0.0439	24	2.34 (1.22–4.48)		0.0104	24
miR-205-5p	0.73 (0.63–0.86)	0.0001	37	NS			
miR-450b-5p	NS			0.48 (0.23–0.99)		0.0458	26
Under median vs. over median							
	Unadjusted			Adjusted			
miRNA	HR (CI)	P	N	HR (CI)		P	N
miR-34a-5p	0.40 (0.19–0.86)	0.0183	53	0.36 (0.16–0.85)		0.0195	52

*NS* Not significant

Table 5 shows 30 and 27 combinations of 2 miRNAs significantly associated with short OS in an unadjusted and an adjusted analysis in PDAC and A-AC in combination.

**Table 5:** Differences of miRNA

Unadjusted effects on differences					Adjusted effects on differences				
miRNA1	miRNA2	HR (CI)	P	N	miRNA1	miRNA2	HR (CI)	P	N
PDAC + AAC									
miR-148a	miR-212-3p	1.20 (1.09–1.33)	0.0002	155	miR-34a-5p	miR-148a	0.82 (0.73–0.92)	0.0011	144
miR-205-5p	miR-769-5p	0.90 (0.85–0.95)	0.0003	129	miR-205-5p	miR-769-5p	0.91 (0.85–0.96)	0.0015	119
miR-148a	miR-205-5p	1.08 (1.04–1.13)	0.0004	130	miR-146a-5p	miR-212-3p	1.33 (1.11–1.60)	0.0017	145
miR-34a-5p	miR-148a	0.83 (0.75–0.92)	0.0009	155	miR-34a-5p	miR-187	0.67 (0.52–0.88)	0.0038	44
miR-34a-5p	miR-187	0.64 (0.50–0.83)	0.0009	47	miR-148a	miR-205-5p	1.07 (1.02–1.12)	0.004	120
miR-146a-5p	miR-212-3p	1.32 (1.12–1.57)	0.0013	156	miR-29a-5p	miR-205-5p	1.08 (1.03–1.15)	0.0046	119
miR-187	miR-212-3p	1.55 (1.18–2.04)	0.0016	47	miR-125a-3p	miR-769-5p	0.81 (0.69–0.94)	0.0071	140
miR-34a-5p	miR-769-5p	0.74 (0.62–0.89)	0.0017	154	miR-187	miR-212-3p	1.47 (1.11–1.96)	0.0078	44
miR-212-3p	miR-769-5p	0.81 (0.70–0.92)	0.0020	154	let-7 g	miR-187	0.74 (0.59–0.93)	0.0085	44
miR-205-5p	miR-625-5p	0.91 (0.86–0.97)	0.0023	72	miR-146a-5p	miR-205-5p	1.08 (1.02–1.14)	0.0097	120
miR-205-5p	miR-450b-5p	0.91 (0.86–0.97)	0.0031	94	miR-205-5p	miR-222-5p	0.93 (0.87–0.99)	0.0152	117
miR-146a-5p	miR-205-5p	1.08 (1.03–1.14)	0.0033	130	miR-29a-5p	miR-769-5p	0.81 (0.68–0.96)	0.0171	143
miR-205-5p	miR-222-5p	0.92 (0.86–0.97)	0.0034	127	let-7 g	miR-205-5p	1.07 (1.01–1.13)	0.018	120
let-7 g	miR-205-5p	1.08 (1.02–1.14)	0.0048	130	miR-29a-5p	miR-194-3p	0.68 (0.50–0.94)	0.0188	46
miR-194-3p	miR-205-5p	1.26 (1.07–1.48)	0.0062	36	miR-125a-3p	miR-187	0.76 (0.61–0.96)	0.0188	43
miR-29a-5p	miR-205-5p	1.07 (1.02–1.13)	0.0072	129	let-7 g	miR-212-3p	1.14 (1.02–1.28)	0.0233	144
miR-125a-3p	miR-205-5p	1.08 (1.02–1.15)	0.0074	128	miR-125a-3p	miR-205-5p	1.07 (1.01–1.14)	0.0236	118
let-7 g	miR-187	0.82 (0.70–0.95)	0.0093	47	miR-205-5p	miR-450b-5p	0.93 (0.87–0.99)	0.024	85
miR-34a-5p	miR-205-5p	1.07 (1.02–1.13)	0.0125	130	miR-34a-5p	miR-194-3p	0.64 (0.43–0.94)	0.0262	45
miR-125a-3p	miR-148a	0.90 (0.83–0.98)	0.0139	152	miR-194-3p	miR-212-3p	1.39 (1.04–1.85)	0.0273	45
miR-125a-3p	miR-769-5p	0.84 (0.73–0.97)	0.0146	151	miR-212-3p	miR-625-5p	0.86 (0.75–0.98)	0.0298	74
miR-125a-3p	miR-187	0.80 (0.66–0.96)	0.0155	46	miR-34a-5p	miR-205-5p	1.07 (1.01–1.13)	0.0307	120
miR-212-3p	miR-625-5p	0.87 (0.77–0.98)	0.0194	79	miR-194-3p	miR-205-5p	1.21 (1.02–1.45)	0.0326	33
let-7 g	miR-212-3p	1.12 (1.01–1.25)	0.0332	155	miR-625-5p	miR-944	1.51 (1.03–2.22)	0.0339	20
miR-187	miR-194-3p	1.41 (1.02–1.96)	0.0366	21	miR-125a-3p	miR-148a	0.91 (0.84–1.00)	0.0383	141
miR-205-5p	miR-212-3p	0.95 (0.90–1.00)	0.0410	130	miR-146a-5p	miR-769-5p	0.84 (0.71–1.00)	0.0394	144
miR-34a-5p	miR-625-5p	0.88 (0.78–1.00)	0.0443	79	miR-34a-5p	miR-625-5p	0.87 (0.75–1.00)	0.0478	74
miR-146a-5p	miR-187	0.79 (0.63–1.00)	0.0452	47					
miR-187	miR-205-5p	1.12 (1.00–1.26)	0.0468	38					
miR-34a-5p	miR-146a-5p	0.83 (0.68–1.00)	0.0488	156					
PDAC									
miR-148a	miR-212-3p	1.18 (1.04–1.33)	0.0077	103	miR-34a-5p	miR-769-5p	0.63 (0.47–0.84)	0.002	92
miR-34a-5p	miR-148a	0.86 (0.76–0.97)	0.0156	103	miR-29a-5p	miR-187	1.99 (1.20–3.29)	0.0072	20
miR-34a-5p	miR-769-5p	0.75 (0.59–0.96)	0.0199	102	miR-187	miR-769-5p	0.54 (0.33–0.87)	0.0111	20
miR-146a-5p	miR-212-3p	1.26 (1.01–1.56)	0.0371	103	miR-187	miR-205-5p	0.72 (0.56–0.94)	0.0138	19
miR-34a-5p	miR-146a-5p	0.74 (0.56–0.99)	0.0427	103	miR-212-3p	miR-769-5p	0.75 (0.60–0.95)	0.0153	92
					miR-148a	miR-212-3p	1.18 (1.03–1.34)	0.016	93
					miR-450b-5p	miR-944	1.56 (1.06–2.30)	0.0243	24
					miR-34a-5p	miR-148a	0.86 (0.75–0.99)	0.0341	93
					miR-146a-5p	miR-212-3p	1.29 (1.02–1.63)	0.0343	93
					miR-148a	miR-431-5p	1.32 (1.02–1.72)	0.0364	34
					miR-146a-5p	miR-187	1.57 (1.01–2.44)	0.0438	20
					miR-222-5p	miR-769-5p	0.84 (0.70–1.00)	0.0491	92
A-AC									
miR-205-5p	miR-769-5p	0.71 (0.60–0.84)	*<*0.0001	36	miR-34a-5p	miR-769-5p	0.51 (0.32–0.81)	0.0043	51
miR-34a-5p	miR-187	0.44 (0.27–0.72)	0.0011	24	miR-125a-3p	miR-187	0.37 (0.18–0.75)	0.0055	23
miR-148a	miR-205-5p	1.25 (1.09–1.44)	0.0018	37	miR-34a-5p	miR-187	0.48 (0.28–0.82)	0.0067	24
miR-125a-3p	miR-187	0.69 (0.54–0.88)	0.0032	23	miR-148a	miR-187	0.59 (0.40–0.87)	0.0074	24
miR-187	miR-205-5p	1.35 (1.10–1.66)	0.0041	17	miR-29a-5p	miR-769-5p	0.65 (0.48–0.89)	0.0077	52
miR-187	miR-212-3p	2.22 (1.29–3.82)	0.0042	24	miR-222-5p	miR-450b-5p	2.12 (1.18–3.81)	0.0123	25
miR-205-5p	miR-450b-5p	0.73 (0.59–0.91)	0.0045	22	miR-187	miR-769-5p	2.09 (1.16–3.78)	0.0148	24
let-7 g	miR-205-5p	1.28 (1.07–1.52)	0.006	37	miR-29a-5p	miR-187	0.62 (0.42–0.91)	0.0154	24
miR-146a-5p	miR-205-5p	1.19 (1.05–1.34)	0.0065	37	miR-187	miR-212-3p	2.23 (1.16–4.30)	0.016	24
let-7 g	miR-769-5p	0.74 (0.59–0.93)	0.0083	52	miR-146a-5p	miR-187	0.54 (0.33–0.90)	0.0175	24
miR-34a-5p	miR-769-5p	0.66 (0.48–0.91)	0.0122	52	miR-148a	miR-450b-5p	2.12 (1.14–3.96)	0.0181	26
miR-34a-5p	miR-205-5p	1.22 (1.04–1.43)	0.0126	37	miR-450b-5p	miR-769-5p	0.31 (0.12–0.84)	0.0214	26
let-7 g	miR-187	0.77 (0.62–0.95)	0.017	24	miR-34a-5p	miR-625-5p	0.71 (0.52–0.96)	0.0267	30
let-7 g	miR-625-5p	0.74 (0.58–0.95)	0.0175	31	miR-125a-3p	miR-769-5p	0.75 (0.58–0.97)	0.0283	49
miR-125a-3p	miR-205-5p	1.21 (1.03–1.43)	0.0227	36	miR-29a-5p	miR-625-5p	0.74 (0.56–0.99)	0.0408	30
let-7 g	miR-222-5p	0.80 (0.67–0.97)	0.0242	50	miR-205-5p	miR-222-5p	0.81 (0.66–0.99)	0.0436	33
miR-29a-5p	miR-187	0.74 (0.56–0.97)	0.0272	24					
miR-205-5p	miR-212-3p	0.86 (0.75–0.98)	0.0289	37					
miR-146a-5p	miR-187	0.67 (0.46–0.96)	0.0308	24					
miR-187	miR-769-5p	1.47 (1.02–2.11)	0.0367	24					
miR-450b-5p	miR-769-5p	0.59 (0.35–1.00)	0.0489	27					

### Prognostic miRNAs - PDAC

**Fig. 4:**
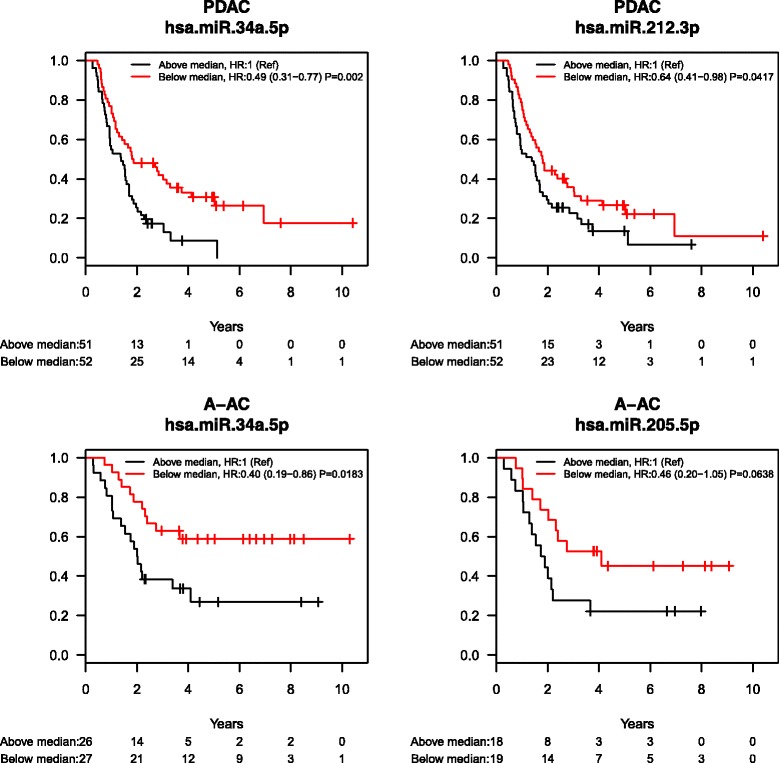
Kaplan–Meier curves for miRNAs significantly associated to survival in patients with PC and patients with A-AC

One hundred three patients with PDAC were available for the survival analysis, and 83 died during the follow-up period. In both the unadjusted and the adjusted (age, sex, tumor stage/differentiation, ASA-score) analyses, low expression of 2 miRNAs was associated with short OS prognosis:miR-34a-5p: HR = 0.72(CI: 0.56–0.93) (unadjusted) and HR = 0.70(CI: 0.52–0.93) (adjusted); and miR-212-3p HR = 0.83(CI: 0.71–0.99) (unadjusted) and HR = 0.82(CI: 0.68–0.99) (adjusted). Dividing the patients into 2 groups for each miRNA (defined as expression under or above the median level), low miR-34a-5p and miR-212-3p levels were associated with short OS. Figure 4 shows Kaplan–Meier curves for the miRNAs reaching a significance level below 0.01.

Table 5 shows 5 and 12 combinations of 2 miRNAs significantly associated with short OS in an unadjusted and an adjusted analysis in PDAC.

### Prognostic miRNAs – A-AC

Fifty-four patients with A-AC were available for the survival analysis, and 29 died during the follow-up period. In the unadjusted analysis, 4 miRNAs were significantly associated with prognosis: let-7 g: HR = 0.74(CI: 0.58–0.93), miR-34a-5p: HR = 0.66(CI: 0.46–0.94), miR-187: HR = 1.51(CI: 1.01–2.24), and miR-205-5p: HR = 0.74(CI: 0.63–0.86). In the adjusted analysis (age, sex, tumor stage/differentiation, ASA-score), low expression of miR-34a-5p: HR = 0.58(CI: 0.38–0.89) and miR-450b-5p: HR = 0.48(CI: 0.23–0.99) and high expression of miR-187: HR = 2.34(CI: 1.22–4.48) were associated with short OS. When patients were divided into 2 groups for each miRNA (defined as expression under or above the median level), low expression of miR-34a-5p was associated with short OS. Figure 4 shows Kaplan–Meier curves for the miRNAs reaching a significance level below 0.01.

Table 5 shows 21 and 16 combinations of 2 miRNAs in A-AC FFPE tissue significantly associated with short OS in both an unadjusted and an adjusted analysis.

## Discussion

In the present study, our aim was to validate previously described tissue miRNA expression profiles as diagnostic and prognostic biomarkers of PC and other periampullary cancers [20–32]. We used non-microdissected FFPE tissue from 165 patients who had undergone surgery for PDAC and from 86 patients who had undergone resection for other periampullary cancers.

Many of the diagnostic miRNAs described in the literature [20, 21, 34] could be validated. We found the following miRNAs either upregulated or downregulated in PC tissue compared to tissue from CP and/or normal pancreas, upregulated miRNAs: miR-21-5p, −23a-3p, −31-5p, −34c-5p, −93-3p, −135b-3p, −155-5p, −186-5p, −196b-5p, −203, −205-5p, −210, −222-3p, −451, −492, −614, and miR-622; and downregulated miRNAs: miR-122-5p, −130b-3p, −216b, −217, and miR-375. Furthermore, we validated the two-miRNA index “miR-196b – miR-217” [27], and suggested new diagnostic indices for separating patients with PC vs. HS and PC vs. HS and CP combined. We found that these indices were useful in discriminating other upper gastrointestinal cancers (duodenal cancer, common bile duct cancer and gastric cancer) from normal pancreas and CP.

In addition to the diagnostic miRNAs, we demonstrated the association of 10 miRNAs with prognosis and constructed several indices based on differences of 2 miRNA associated with poor prognosis.

A major limitation of the study was the high number of non-detectable miRNAs using the Fluidigm BioMark System™. Even though we purified the miRNAs from FFPE by the same method as in our previous studies [27, 31] and repeated the analysis several times, we still experienced a high number of undetectable miRNAs. At present, we have no explanation for this problem apart from possible platform sensitivity limitations.

We consider it a strength of the study that non-microdissected samples were used, since this will also be the case in a clinical setting. The tumor microenvironment is a highly dynamic component of PC, often constitutes the bulk of the tumor, and should therefore be taken into account. The extracellular stroma participates in paracrine signaling that promotes PDAC cell survival and metastasis, and the dense extracellular matrix characteristic of PDAC acts as a physical barrier to infiltrating immune cells and the diffusion of chemotherapy [35–37]. MicroRNAs are involved in the regulation of the extracellular components in different tissues [38, 39]. Since many studies regarding miRNAs in PC are performed on microdissected tissue or cell lines the miRNAs originating from the extracellular stroma are less elucidated. The following miRNAs significantly deregulated in the present study are known to be related to the extracellular compartment of PC: miR-21, −29, −130b, −210, and-451 [40–43].

Among the validated miRNAs, high expression of miR-21, miR-31, and miR-155 and low expression of miR-217 and miR-375are the most consistently described dysregulated miRNAs in PC. Several studies have found miR-155to be upregulated in PC [20–22, 28, 32, 44, 45]. miR-155 functions as an onco-miRNA in different types of cancer,e.g., breast, cervix, colon, and lung cancer, and high miR-155 expression in cancer tissue is associated with poor prognosis in PC and lung cancer [30, 46–49]. The oncogenic effect of miR-155 maybe caused by the targeting of anti-inflammatory signal pathways such as Sh2 domain-containing inositol phosphatase-1 (Ship1) or from suppression of cytokine signaling 1 (Socs1) [50, 51].

miR-21 is also an onco-miR involved in PC tumorigenesis, invasion, metastasis, and chemoresistance [20, 21, 23, 27, 32, 44, 45, 52–57]. miR-21 is primarily upregulated in the extracellular stroma, which is considered a dynamic component of PC, and high expression is associated with poor prognosis [40]. Our study was conducted on non-microdissected tissue and thus also detects miRNAs in the extracellular stroma.miR-21 targets tumor suppressors like PTEN, PDCD4, and TIMP3, components of the p53 pathway, and modulates TGF-b signaling, thus promoting cell proliferation, survival, and migration/invasion [45, 58–60].

miR-31 is upregulated in PC [21, 27, 28, 45, 61]. miR-31 targets human mutL homolog 1 (a mismatch repair protein) [62] and activates the RAS pathway by inhibiting RAS p21 GTPase activating protein 1 (RASA1) in colorectal cancer [63].

miR-217 is downregulated in PC and in pancreatic intraepithelial neoplasm (PanIN) [21, 27, 28, 32, 45, 64]. This finding has also been replicated in studies using fine needle aspirates from PC [24, 65].miR-217 acts as a tumor suppressor in PC by targeting *KRAS* [66] and is involved in epithelial-mesenchymal-transition (EMT) in PC and CP via the miR-217-SIRT1 pathway, which can be triggered by TGF-β1 in inflammatory processes [67].

miR-375 is downregulated in PC compared to normal pancreas, is associated with prognosis, and can differentiate between pancreatobiliary and intestinal subtypes in ampullary adenocarcinoma [20, 21, 27, 28, 32, 68]. miR-375 is also downregulated in esophageal, gastric, breast, lung, colorectal, and cervical cancers [69–74]. miR-375 plays a role in the development and maintenance of the α- and β-cell mass in the normal pancreas and is upregulated in patients with type 2 diabetes [75, 76].miR-375 targets 3-phosphoinositide-dependent protein kinase-1 (PDK1) in PC and inhibits PC cell proliferation in vitro [77, 78].

In the literature, the following miRNAs are described as prognostic after PC resection:Let-7 g, miR-21, miR-29a-5p, miR-34a-5p, miR-146a, miR-155, miR-196a, miR-203, miR-205, miR-210, miR-212, miR-222, miR-450b-5p, and miR-675 [23, 29–32]. We have previously described prognostic indices using combinations of high expression of miR-212 and miR-675 and low expression of miR-148a-5p (previous ID: miR-148a*), miR-187 and let-7 g-3p (previous ID: let-7 g*) in FFPE tissue from patients operated for PC [31]. Only a few of these patients received adjuvant chemotherapy after surgery. In the present study, patients with PDAC and A-AC were all treated with adjuvant gemcitabine for 6 months or until disease recurrence. In this population, we could validate let-7 g, miR-29a-5p, miR-34a-5p, miR-146a-5p,miR-205-5p, and miR-212-3pas prognostic biomarkers after radical resection for PC.

The let-7 family of miRNAs includes tumor suppressor miRNAs, the expression of which is prognostic in HCC, gastric, and ovarian cancers [79–81]. Let-7 g is involved in pathways essential for the development of cancer. It targets Fas and is involved in Fas-mediated apoptosis [82]. Silencing of let-7b/g activates AKT signaling and promotes carcinogenesis in gastric cancer [83]. Let-7 inhibits cell motility in breast cancer by regulating genes in the cytoskeleton pathway and silencing of let-7 promotes metastases [84]. Let-7 inhibits proliferation in HCC by downregulation of c-Myc and upregulation of p16(INK4A) [85].

In PC, miR-29a-5p induces EMT, stimulates pancreatic stellate cells to accumulate protein in the extracellular matrix, and increases resistance to gemcitabine through the Wnt/beta-catenin pathway [41, 86, 87]. miR-34a is upregulated in cervical and colorectal cancers and downregulated in breast, prostate, renal and lung cancer [49, 88].

The miR-34 family miRNAs are described as tumor suppressor miRNAs, and miR-34a/c suppresses breast cancer invasion and metastasis by targeting Fos-related antigen-1 [89]. PC mouse models show that miR-146a acts through EGFR signaling [90]. miR-205 is involved in EMT and acts through the anti-apoptotic protein Bcl-2 (in prostate cancer) and HER3 (in breast cancer) [91–93]. We found that low expression of miR-125a-3p was associated with short OS in patients with PC, and this is a novel observation.miR-125a-3p has been described as a tumor suppressor miRNA in several cancers [94, 95].

In the present study, miR-130b was found to be downregulated in PDAC compared to benign specimens. Interestingly, this miRNA is upregulated in the stroma compared to carcinoma cells [42].

Further information about the 46 miRNAs analyzed in the present study is given in “Additional file 1”.

## Conclusions

In conclusion, we could validate miRNAs selected from the literature as diagnostic and/or prognostic biomarkers in patients radically resected for PC. No microdissection of the tumors was done, and some of the miRNAs most likely originated from the stroma and not the cancer cells. The diagnostic ability of these miRNAs was also tested on duodenal cancer, common bile duct cancer, and gastric cancer – diagnoses that represent a considerable diagnostic challenge in separating from PC in a clinical setting. Hopefully, this study can contribute to the understanding of pancreatic and periampullary cancers and improve the diagnosis, prognosis, and ultimately treatment of patients with these conditions. For example, this could be achieved by allocating young patients with a miRNA expression profile suggestive of poor prognosis to a more aggressive chemotherapy regimen, or elderly patients with a more promising prognostic profile could be spared from adjuvant therapy.

## Additional files

Additional file 1: Background on all measured microRNA. (DOCX 1012 kb)
Additional file 2: All statistical calculations including insignificant results not presented in the manuscript. (DOC 1481 kb)

## Abbreviations

A-AC: Ampullary adenocarcinoma
CBD: Common bile duct
CP: Chronic pancreatitis
DC: Duodenal cancer
FFPE: Formalin-fixed paraffin-embedded
GC: Gastric cancer
HS: Healthy subjects
miR: microRNA
miRNA: microRNA
PC: Pancreatic cancer
PDAC: Pancreatic ductal adenocarcinoma
