# Supplementary material for: Tissue MicroRNA profiles as diagnostic and prognostic biomarkers in patients with resectable pancreatic ductal adenocarcinoma and periampullary cancers
Source: Biomark Res. 2017 Feb 21;5:8. doi: 10.1186/s40364-017-0087-6 (PMC5320745; doi:10.1186/s40364-017-0087-6)
Supplement: Additional file 1: — Background on all measured microRNA. (DOCX 1012 kb) [file 40364_2017_87_MOESM1_ESM.docx]

**microRNA Characteristics**

This is an additional file for the paper: *”Tissue MicroRNA Profiles as Diagnostic and Prognostic Biomarkers in Patients with Resectable Pancreatic Ductal Adenocarcinoma and Periampullary Cancers”* by Dan Calatayud et al.

| **microRNA** | **Present study** | **Previously described in pancreatic cancer** | **Other cancers/conditions** | **Potential target/pathway** | **Ref** |
| --- | --- | --- | --- | --- | --- |
| let-7g | Prognostic in AAC (in unadjusted analyses). | Prognostic (↓). | Let 7 family is downregulated in leukemias and lymphomas and breast, colon, liver, lung, RCC, esophagus, ovarian, prostate, gastric HCC, atypical teratoid rhabdoid tumor, and testicular cancer, liposarcoma, naso-pharyngeal carcinoma.  ↓ is a poor prognostic factor in HCC, gastric cancer and breast cancer.  Related to chemo-resistance in gastric cancer. | Tumor suppressor.  let-7g may act as a tumor suppressor gene that inhibits HCC cell proliferation by downregulating the oncogene c-Myc, and upregulating the tumor suppressor gene p16(INK4A).  Regulation of cell proliferation, RAS-ERK/MAP kinase signaling.  Regulate Fas and Fas-mediated apoptosis.  Let-7 directly inhibits IL-6.  Decreased let-7b/g contributes to aberrant AKT activation in gastric tumorigenesis.  Downregulates telomerase activity.  Downregulated in CAFs (Cancer associated fribroblasts).  Let-7g suppresses nuclear factor-kappa B1 (NFkB1).  LIN28 can directly inhibit let-7g biogenesis at the Dicer processing step. | [[1-25](#_ENREF_1)] |
| miR-21-5p | ↑ in PC vs. HS.  ↑ in PC vs. HS+CP.  Part of diagnostic index VI. | ↑ in PC.  Diagnosis and prognosis. Stage, grade and lymph node status.  Predictive.  Involved in chemoresistance.  ↑ in extracellular stroma in PC.  miR-21 acquired from cysts is associated with invasive cancer.    Stromal miR-21 levels predict response to 5-FU.  Associated with liver metastasis.  Possible therapeutic target.  ↑ in PanIN. | ↑ in almost all carcinomas and hematological malignancies and linked to poor prognosis.  Identified in plasma. | Decrease anti-tumor immunity:  Targets tumor suppressors such as PTEN/AKT, PDCD4, and p53 pathway.  Targets TIMP3 in pancreatic cancer.  Related to Notch1 an important regulator of EMT in PDAC.  Induces upregulation of Bcl-2 that is associated with apoptosis, chemoresistance and proliferation in PC.  Upregulated due to hypoxia in PC via HIF-1α.  Increase Nuclear Factor κB.  Anti-inflammatory:  Modulating TGF-β => proliferation/survival/migration.  Suppression inhibits tumor growth via TPM1 in breast cancer. | [[11](#_ENREF_11), [14](#_ENREF_14), [26-57](#_ENREF_26)] |
| miR-23a-3p | ↑ in PC vs. HS.  ↑ in PC vs. HS+CP. | ↑ in PC.  LASSO classifier. | ↑ in lung, gastric and colorectal cancer.  ↓ in oral squamous cell cancer. | Acts oncogenic via inhibiting APAF1 in PDAC and CRC.  Overexpression of miR-23a in PDAC leads to EMT like cell formation.  Down-regulating metastasis suppressor 1 (MTSS1) and thereby promotes migration in CRC.  Growth promoting via the IL6-receptor.  Regulates cell-proliferation via the Wnt pathway.  Targeting SMAD-genes.  Targets the PAK6-LIMK1 pathway in prostate cancer.  Inhibits topoisomerase 1 in HCC.  c-MYC-regulated miR-23a/24-2/27a cluster promotes mammary carcinoma cell invasion and hepatic metastasis by targeting Sprouty2.  Regulates TGF-β-induced epithelial-mesenchymal transition by targeting E-cadherin in lung cancer cells. | [[27](#_ENREF_27), [44](#_ENREF_44), [58-71](#_ENREF_58)] |
| miR-29a-5p | Prognostic in PC+ACC. | Prognostic (low) in PDAC. | ↑ in CRC and potential plasma-marker.  ↓ in gastric cancer, cervical, breast.  Prognostic in CRC. | Activator of the Wnt/β-catenin pathway.  Induces EMT in pancreatic cancer via CEACAM6 (Carcinoembryonic antigen-related cell adhesion molecule 6).  Induces resistance to gemcitabin in PDAC through the Wnt/β-Catenin.  Induces EMT in HCC via TGF-β.  Regulates expression of the MEG3 (a tumorsuppressor).  Targets AKT2 in gastric cancer.  Suppresses the growth, migration, and invasion of lung adenocarcinoma cells by targeting carcinoembryonic antigen-related cell adhesion molecule 6 (CAECAM6).  Tumorsuppressive in prostate cancer via LAMC1.  Promotes colorectal cancer  metastasis by regulating matrix metalloproteinase 2 and E-cadherin via KLF4.  Tumor suppressive in cervical cancer via targeting HSP47.  Targets B-Myb in breast cancer.  miR-29a regulates the SPARC-AKT pathway in HCC.  miR-29a cell proliferation and induces cell cycle arrest through the downregulation of p42.3 in gastric cancer. | [[1](#_ENREF_1), [72-86](#_ENREF_72)] |
| miR-31-5p | ↑ in PC vs. HS.  ↑ in PC vs. HS+CP.  Part of diagnostic index VI. | ↑ in PC.  ↑ in pancreatic stellate cells.  (One paper reports miR-31 to be downregulated).  Able to differentiate periampullary cancer subtypes. | ↑ in CRC, HCC, cholangiocarcinoma, squamous cell carcinoma of the tongue, head and neck squamous cell carcinoma and lung cancer.  ↓ in bladder cancer, prostate cancer, gastric cancer, breast cancer, HCC, ovarian cancer and CML. | Reported to be tumor suppressive and as well as oncogenic.  Regulates cell cycle by directly targeting HDAC2, CDK2 and C-MYC.  Regulates EMT via N-cadherin, E-cadherin, vimentin and fibronectin.  Both inhibition and enhanced expression of miR-31 lead to reduced migration and invasion of pancreatic cancer cells.  Modulates cell cycle by targeting human mutL homolog 1 (a mismatch repair protein).  Induces cell cycle arrest in the S-phase (demonstrated in cell lines).  Directly targets RAS p21 GTPase activating protein 1 (RASA1) (demonstrated in CRC).  Targets tumor suppressor RhoBTB1 (demonstrated in CRC cell lines)  Correlated with CA19-9 and associated with mutations in the APC gene (In CRC).  Repression of several integrins. | [[26](#_ENREF_26), [34](#_ENREF_34), [35](#_ENREF_35), [44](#_ENREF_44), [87-106](#_ENREF_87)] |
| miR-34a-5p | Prognostic in PC+AAC, PC, AAC. | ↑ in PC.  Prognostic.  Involved in chemoresistance. | ↑ in cervical cancer and CRC.  ↓ in breast cancer, NSCLC, prostate cancer and renal cell carcinoma. | Tumor suppressor miR.  Critical regulator of the pancreatic cancer stem cell.  Targets Notch1, SIRT1, and CD44 pathway in PDAC.  Associated with mitogen-activated protein kinase (MAPK) in PDAC.  miR-34a/c targets PDGF-receptor (cell surface tyrosine kinase receptors that induce proliferation, migration and invasion).  Inactivation of miR-34a/b/c by CpG methylation in colorectal, pancreatic, mammary, ovarian, urothelial, and renal cell carcinomas and soft tissue sarcomas.  miR-34a/c suppresses breast cancer invasion and metastasis by directly targeting Fos-related antigen 1.  miR-34a/c control cancer cell expression of ULBP2, a stress ligand of the NK cell receptor NKG2D.  p53 directly targets the miR-34 family, but the p53 pathway has shown to be intact in miR-34 deficient mice.  ZEB1 drives prometastatic actin cytoskeletal remodeling by downregulating miR-34a.  miR-34a downregulates Ras signaling by targeting IMPDH (inosine 5’-monophosphate dehydrogenase), which is involved in GTP synthesis.  miR-34a suppress malignant transformation in renal cell carcinoma and prostate by targeting the c-Myc-Skp2-Miz1 complex and thereby suppressing RhoA (a regulator of migration and invasion).  p53 downregulates the EMT inducing transcription factor SNAIL via miR-34a/b/c.  miR-34 targets BCL2, NOTCH, and HMGA2 in gastric cancer.  During senescence, miR-34a targets MYC and controls a  set of cell-cycle regulators in fibroblasts.  miR-34a targets the AXL receptor and is silenced by promoter methylation in lung.  miR-34 targets MET I in ovarian cancer . | [[1](#_ENREF_1), [33](#_ENREF_33), [37](#_ENREF_37), [44](#_ENREF_44), [107-124](#_ENREF_107)] |
| miR-34c-5p | ↑ in PC vs. HS+CP.  ↑ in PC vs. HS.  Part of diagnostic index I, II, III, IV. | LASSO classifier. | ↓ in most cancers including prostate, NSCLC, breast. | Tumor suppressor-miR.  Targets c-MET, a tyrosine kinase activated by hepatocyte growth factor, that is important in metastatic progression.  Inactivation of miR-34a/b/c by CpG methylation in colorectal, pancreatic, mammary, ovarian, urothelial, and renal cell carcinomas and soft tissue sarcomas.  miR-34a/c targets PDGF-receptor (cell surface tyrosine kinase receptors that induce proliferation, migration and invasion).  miR-34a/c suppresses breast cancer invasion and metastasis by directly targeting Fos-related antigen 1.  miR-34a/c control cancer cell expression of ULBP2, a stress ligand of the NK cell receptor NKG2D.  Downregulation of miR-34c promotes EMT in breast tumor-initiating cells.  Negative regulation of the oncogenes Bcl-2 and E2F3 in prostate cancer.  p53 directly targets the miR-34 family, but the p53 pathway has shown to be intact in miR-34 deficient mice.  p53 downregulates the EMT inducing transcription factor SNAIL via miR-34a/b/c.  miR-34 targets BCL2, NOTCH, and HMGA2 in gastric cancer.  During senescence, miR-34a targets MYC and controls a  set of cell-cycle regulators in fibroblasts. | [[23](#_ENREF_23), [27](#_ENREF_27), [37](#_ENREF_37), [108-111](#_ENREF_108), [113](#_ENREF_113), [118](#_ENREF_118), [123](#_ENREF_123), [125-128](#_ENREF_125)] |
| miR-93-3p | ↑ in PC vs. HS.  ↑ in PC vs. HS+CP.  Part of diagnostic index IV. | 2-miR-test | ↑ in gastric, breast and esophageal cancer, NSCLC.  ↓ in colon cancer, ovarian.  Prognostic in gastric cancer. | Regulates PTEN/Akt signaling pathway in ovarian cancer and HCC  Promotes angiogenesis and metastasis by silencing the LATS2 tumor suppressor and integrin-β8 (breast cancer).  Targets RhoC in ovarian cancer.  MicroRNA-93 suppresses colorectal cancer development via Wnt/β-catenin pathway downregulating.  Downregulation of DAB2 defines a novel oncogenic pathway in lung cancer.  Promoting metastases in breast cancer  Involved in the progression from cirrhosis to HCC. | [[27](#_ENREF_27), [32](#_ENREF_32), [129-143](#_ENREF_129)] |
| miR-122-5p | ↓ in PC vs. HS (many missing values).  ↓ in PC vs. HS+CP.  Part of diagnostic index V, VI. | ↓ in PC.  LASSO classifier.  2-miR-test. | ↑ in prostate cancer.  ↓ in cholangiocarcinoma, breast cancer.  Described as “a liver-specific microRNA implicated in regulation of fatty acid and cholesterol metabolism, hepatitis C infection, and hepatocellular carcinoma”. Reported ↑ as well as ↓ in HCC. | Targets cationic amino acid transporter 1 (CAT1) in CRC metastasis.  Tumor suppressor through targeting IGF1-receptor and PI3K/akt/mTOR/p70S6K pathway.  Acts via the Wnt/β-Catenin pathway in HCC and Glomma.  Targets hepatitis C and considered a potential therapeutic.  Breast-cancer-secreted miR-122 reprograms glucose metabolism in premetastatic niche to promote metastasis.  Overexpression of microRNA-122 re-sensitizes 5-FU-resistant colon cancer cells to 5-FU through the inhibition of PKM2 in vitro and in vivo.  Negative feedback loop tumor suppressor miR-122 and oncogene c-Myc.  The HNF4α/miR-122/RhoA axis negatively regulates EMT and the migration and invasion of HCC cells.  miR-122 targets pyruvate kinase M2 and affects metabolism of hepatocellular carcinoma.  MicroRNA-122 promotes proliferation, invasion and migration of renal cell carcinoma cells through the PI3K/Akt signaling pathway. | [[27](#_ENREF_27), [34](#_ENREF_34), [35](#_ENREF_35), [125](#_ENREF_125), [144-160](#_ENREF_144)] |
| miR-125a-3p | Prognostic in PC+AAC.  Part of diagnostic index III, VI. |  | ↓ in breast, lung and gastric cancer, NSCLC, and neuroblastoma.  ↓ in CRC (but ↑ in patients who responds the neoadjuvant radio-chemotherapy in CRC). | Tumor suppressor miR.  Inhibits cell proliferation through the Fyn/FAK-pathway.  activate the NF-dB pathway by targeting the tumor necrosis factor alpha-induced protein 3.  induces apoptosis by activating p53 in lung cancer cells.  Tumorsuppressive in lung cancer via Epidermal Growth Factor. | [[161-171](#_ENREF_161)] |
| miR-130b-3p | ↓ in PC vs. HS.  ↓ in PC vs. HS+CP.  Part of diagnostic index I, IV, V, VI. | ↓ and prognostic in PC. | ↑ in head and neck squamous cell carcinoma.  ↓ in gastric, prostate and thyroid cancer. | Promotes EMT in endometrial cancer by targeting DICER1.  p53 induces EMT via the miR-130b-ZEB1 axis.  miR-130b is a potential target of p53 in endometrial cancer.  Regulated the tumor suppressor RUNX3 I gastric cancer.  Acts oncogenic by repressing PTEN in esophageal squamous cell carcinoma.  Tumorsuppressive in prostate cancer through down-regulation of MMP2.  Tumorsuppressive in CRC through downregulation of integrin β1. | [[172-181](#_ENREF_172)] |
| miR-135b-3p | ↑ in PC vs. HS.  ↑ in PC vs. HS+CP. | LASSO classifier. | ↑ in CRC, gastric, breast, thyroid and prostate cancer.  Correlates with survival and early metastazation in breast cancer.  Correlates to stage in CRC.  Potential serum biomarker in CRC. | Oncogenic function mediated through repression of multiple components in the Hippo pathway (a kinase cascade) and the tumor suppressor LZTS1 in lung cancer cell lines.  miR-135b is upregulated by NF-κB, which is upregulated by TNF-α.  Induce Wnt signaling in CRC by suppression of APC.  Involved in PTEN/PI3K pathway in CRC.  Targets Midline1 and Mitochondrial Carrier Homolog2 in breast cancer cells in mice.  Promotes invasion/metastasis in NSCLC.  Contributes to angiogenesis in lymphoma. | [[27](#_ENREF_27), [182-192](#_ENREF_182)] |
| miR-136-3p |  | LASSO classifier. | ↑ in lung cancer.  ↓ in gliomas. | Tumor suppressor.  Targets anti-apoptotic genes AEG-1 and Bcl-2. | [[27](#_ENREF_27), [92](#_ENREF_92), [193](#_ENREF_193), [194](#_ENREF_194)] |
| miR-146a-5p | Prognostic in PC+AAC. | ↓ in PC.  Linked to stage, grade and lymph node status  2-year survival.  ↓ in PanIN.  ↑ in FNAC from PC. | ↑ in breast cancer, thyroid, and cervical cancer.  ↓ in HCC, gastric cancer and prostate cancer.  Well described in autoimmune disease.  ↑ in response to microbial infection. | Regulates EGFR in a PDAC mouse model.  miR-146 is induced through toll-like receptor and induction is NF-κB dependent.  Involved in invasive growth of PDAC.  Tumorsuppressive in PDAC mouse model by targeting kRas.  miR-146 directly targets TRAF6 and IRAK1 (key molecules in the TLR/NF-κB pathway) suggesting a negative feedback mechanism.  Tumorsuppressive in HCC via downregulating VEGF.  Tumorsuppressive in prostate cancer by targeting Rac1.  Part of the BRCA1/EGFR pathway in breast cancer.  Upregulates COX2 in lung cancer.  Tumorsuppressive in gastric cancer via targeting WASF2.  Directly targeting SMAD4 in gastric cancer. | [[1](#_ENREF_1), [11](#_ENREF_11), [30](#_ENREF_30), [34](#_ENREF_34), [35](#_ENREF_35), [49](#_ENREF_49), [107](#_ENREF_107), [195-205](#_ENREF_195)] |
| miR-148a-3p (148a)  miR-148a-5p (148a*) | Part of diagnostic index VI (miR-148a-3p). | ↓ in PC.  Prognostic in A-AC (low).  Prognostic (low).  Biomarker for PanIN.  Found in serum in a pancreatic cancer mouse model. | ↓ in breast and gastric cancer, NSCLC and HCC.  ↓ related to poor prognosis in gastric cancer.  Potential circulating biomarker in gastric cancer.  ↑ in serum in CRC relates to poor prognosis. | Targets the CCKBR and Bcl-2 in pancreatic cancer.  Regulates cell survival in PDAC by targeting CDC25B.  Silencing of miR-148 is considered an early event in pancreatic carcinogenesis.  Especially downregulated in Kris-mutated cancers.  Downregulated due to hypermethylation of the encoding DNA.  miR-148a directly targets MMP7 in gastric cancer and is related to tumor invasiveness.  Suppresses EMT and metastasis by targeting Met/Snail signaling pathway.  Targets p27 in gastric cancer  Inhibits angiogenesis by targeting ERBB3.  miR-148a in prostate cancer cells. | [[1](#_ENREF_1), [26](#_ENREF_26), [36](#_ENREF_36), [206-219](#_ENREF_206)] |
| miR-155-5p | ↑ in PC vs. HS.  ↑ in PC vs. HS+CP.  Part of diagnostic index III, VI. | ↑ in PC.  Prognostic.  ↑ in PanIN. | ↑ in cervical, breast, lung, thyroid cancer.  ↓ in gastric cancer.  Prognostic in head and neck squamous cell carcinoma, lung cancer.  Well described in the immune system and hematological conditions. | Targets tumor suppressor Sel-1-like (SEL1L) in pancreatic cancer.  Targets Mut L homologue 1 (MLH1) in pancreatic cancer and is related to prognosis.  Described as oncogenic as well as tumor suppressive depending on the tumor system.  Induces EMT via targets Smad1, Smad2, Smad5 and RhoA.  Block apoptosis via targets CASP3, FADD, RIP1, IRAK, PKA, Apaf-1 and FOXO3A.  Targets TP53INP1 in pancreatic cancer, breast cancer and esophageal squamous cell carcinoma.  May serve as a bridge between inflammation and cancer in breast cancer by targeting the SOCS1-STAT1 pathway.  Targets NF-kappa-B transcriptional factor that is involved cell proliferation and tumor development.  Inactivates mismatch repair in colon cancer by targeting hMSH2, hMSH6, hMLH1.  microRNA-155 promotes the proliferation of prostate cancer cells by targeting annexin 7.  miRNA-155 promoted the proliferation of cervical cancer cells by regulating LKB1 expression.  Targets c-Myc in gastric cancer cell lines.  miR-155 drives telomere fragility in human breast cancer by targeting TRF1.  MiR-155-mediated loss of CCAAT-enhancer binding protein beta (C/EBPβ) shifts the TGF-β response from growth inhibition to epithelial-mesenchymal transition, invasion and metastasis in breast cancer. | [[11](#_ENREF_11), [28](#_ENREF_28), [33](#_ENREF_33), [35](#_ENREF_35), [37](#_ENREF_37), [44](#_ENREF_44), [55](#_ENREF_55), [56](#_ENREF_56), [220-234](#_ENREF_220)] |
| miR-186-5p | ↑ in PC vs. HS (not significant).  ↑ in PC vs. HS+CP. | ↑ in PC.  LASSO classifier. | ↓ in esophageal and prostate cancer.  ↓ predicts poor survival in NSCLC. | Tumor suppressor: Inhibits Cyklin D, Cyklin dependent kinase 2 & 6.  Targets PTTG1 and Rho-associated protein kinase 1 (ROCK1) in NSCLC.  Targets the apoptosis inducing Curcumin in lung cancer cell lines. | [[27](#_ENREF_27), [235-241](#_ENREF_235)] |
| miR-187-3p | Prognostic in AAC. | Prognostic (↓). | ↓ in prostate cancer  Prognostic for poor survival in breast cancer (↑). | RAS/EGFR pathway | [[1](#_ENREF_1), [242](#_ENREF_242), [243](#_ENREF_243)] |
| miR-194-3p (194*) |  | Prognostic (2-year) in  A-AC. | ↑ in esophageal cancer, CRC and nephroblastoma.  Suppresses metastasis in NSCLC and gastric cancer. | Regulates expression of BMP1 and p27kip1 in NSCLC.  Inhibits EMT in endometrial cancer by targeting oncogene BMI-1. | [[1](#_ENREF_1), [27](#_ENREF_27), [32](#_ENREF_32), [244-248](#_ENREF_244)] |
| miR-196b-5p | ↑ in PC vs. HS.  ↑ in PC vs. HS+CP. | LASSO classifier.  Prognostic in A-AC  Asuragen test.  Deregulated in PanIN-lesions. | ↑ in CRC, osteosarcoma, HCC, oral cancer, gastric/esophageal cancer and ALL/AML.  ↓ in cervical cancer.  Prognostic in glioblastoma, CRC and lung cancer. | Targets c-myc and Bcl-2 expression, inhibits proliferation and induces apoptosis in endometriosis.  Regulates angiogenesis via endothelial progenitor cells.  Directly targets both HOXA9/MEIS9 oncogenes and FAS tumor suppressor in mixed lineage leukemia.  Metastasis suppressor in breast cancer.  miR-196b/HOXB7/VEGF pathway plays an important role in cervical cancer progression  Targets apoptosis annexin A1 (ANXA1). | [[1](#_ENREF_1), [27](#_ENREF_27), [28](#_ENREF_28), [203](#_ENREF_203), [249-267](#_ENREF_249)] |
| miR-198 |  | LASSO classifier.  2-miR-test. | ↑ in prostate cancer and retinoblastoma.  ↓ in HCC. | Inhibits migration and invasion in HCC by targeting the HGF/c-MET pathway. | [[27](#_ENREF_27), [125](#_ENREF_125), [268](#_ENREF_268)] |
| miR-203 | ↑ in PC vs. HS.  ↑ in PC vs. HS+CP.  Part of diagnostic index II, VI. | ↑ in PC.  ↑ in PanIN.  Prognostic.  LASSO classifier. | ↓ in esophageal, breast, lung, prostate, bladder and cervical cancer.  ↓ in cancer of unknown primary with EMT phenotype.  Involved in chronic inflammatory diseases. | Targets TLR4 and downstream cytokines (TNF-α and IL-12) and caveolin-1 in pancreatic cancer cells.  Targets transcription factor SNAI2 that promotes EMT and metastasis.  Interleukin-8 (in keratinocytes).  Targets E2F1 (involved in cell cycle), Ran, and LASP1 in esophageal cancer.  Targets Rap1A in prostate cancer.  Targets Runx2, BIRC5, and LASP in breast cancer.  Targets BANF1 in cervical cancer.  Targets ZNF217 in CRC.  Targets LIM and SH3 Protein 1 (LASP1), CKAP2, BIRC5, WASF1, ASAP1, RUNX2, and EGFR in prostate cancer.  Targets stem renewal factor Bmi-1 in esophageal cancer.  Targets proto-oncogene SRC and PKCa in lung cancer.  Suppresses growth and angiogenesis via VEGFA in cervical cancer. | [[27](#_ENREF_27), [32](#_ENREF_32), [33](#_ENREF_33), [107](#_ENREF_107), [203](#_ENREF_203), [220](#_ENREF_220), [269-293](#_ENREF_269)] |
| miR-205-5p | ↑ in PC vs. HS.  ↑ in PC vs. HS+CP.  Prognostic in PC+AAC.  Prognostic in AAC (in unadjusted analyses).  Part of diagnostic index VI. | PDAC vs. A-AC  ↑ in PC (Also reported downregulated).  Prognostic.  Elevated in pancreatic juice. | ↑ in squamous cell carcinoma of the lung, ovarian, bladder, and endometrial cancer.  ↓ in breast, gastric, esophageal and prostate cancer.  Prognostic in breast cancer. | Downregulation of miR-205 induces EMT via E-cadherin suppression and the TGF-β pathway.  Targets the anti-apoptotic Bcl2.  Downregulated in CAFs (Cancer Associated Fibroblasts).  inhibits cell apoptosis by targeting phosphatase and tensin homolog deleted on chromosome ten in endometrial cancer cells.  TMPRSS4 (membrane protein involved in metastasis) regulates levels of integrin α5 in NSCLC through miR-205 activity to promote metastasis.  Targets oncoprotein Yin Yang 1 in gastric cancer cell lines.  Promotes growth and metastasis in NSCLC via PTEN.  Regulates anti-apoptotic Bcl2 in prostate cancer.  Promotes invasion in endometrial cancer via targeting ESRRG.  Inhibits Src-mediated oncogenic pathway in renal cancer.  Regulates HER3 in breast cancer. | [[1](#_ENREF_1), [18](#_ENREF_18), [26](#_ENREF_26), [32](#_ENREF_32), [219](#_ENREF_219), [243](#_ENREF_243), [294-312](#_ENREF_294)] |
| miR-210 | ↑ in PC vs. HS.  ↑ in PC vs. HS+CP.  Part of diagnostic index VI. | ↑ in PC.  Diagnostic and prognostic.  Regulates the interaction between pancreatic cancer cells and stellate cells.  Elevated in pancreatic juice. | ↑ in lung cancer, renal, cell carcinoma, CRC, breast and HCC  Prognostic in breast cancer. | Oncogenic in bladder cancer.  Induced by hypoxia via Hypoxia-inducible factor 1-α (HIF): “hypoxamir”.  Upregulation => angiogenesis.  Inhibits cancer cell proliferation by inducing cell death and cell cycle arrest by targeting Fibroblast Growth Factor Receptor-like 1.  Facilitates metastasis in HCC by targeting Vacuole Membrane Protein 1.  Targets cell cycle genes Plk1 and Cdc25B. | [[26](#_ENREF_26), [27](#_ENREF_27), [35](#_ENREF_35), [219](#_ENREF_219), [220](#_ENREF_220), [301](#_ENREF_301), [313-322](#_ENREF_313)] |
| miR-212-3p | Prognostic in PC+AAC, PC.  Part of diagnostic index VI. | ↑ in PC.  Prognostic in PDAC and A-AC (high). | ↑ in NSCLC.  ↓ in prostate cancer.  Reported up- and ↓ in lung cancer.  Promotes metastases from CRC.  Predictive in gastric cancer.  ↑ related to poor prognosis in esophageal cancer. | Considered an oncomiR as well as a tumor-suppressor-miR.  RAS/EGFR pathway and Rb1 tumorsuppressor gene.  Directly targets manganese superoxide dismutase (MnSOD) mRNA.  Negatively regulates the anti-apoptotic protein PED and regulates TRAIL sensitivity.  Targets methyl-CpG-binding protein MeCP2 in gastric cancer.  Targets the hedgehog pathway receptor PTCH1.  Inhibits proliferation of gastric cancer by directly repressing retinoblastoma binding protein 2 | [[1](#_ENREF_1), [125](#_ENREF_125), [323-332](#_ENREF_323)] |
| miR-216b | ↓ in PC vs. HS.  ↓ in PC vs. HS+CP.  Part of diagnostic index VI. | ↓ in PC.  Diagnostic.  Primarily expressed in the acinar cells. | ↓ in nasopharyngeal carcinoma and breast cancer cell lines. | Tumor suppression by targeting kRAS in PDAC.  Promotes cellular senescence through the p53-p21 pathway. | [[27](#_ENREF_27), [49](#_ENREF_49), [219](#_ENREF_219), [333-336](#_ENREF_333)] |
| miR-217 | ↓ in PC vs. HS.  ↓ in PC vs. HS+CP. | ↓ in PDAC and PanIN.  ↓ in FNAC from PDAC.  Asuragen test. | ↓ in clear cell renal carcinoma.  ↑ in HCC.  Correlates with estrogen receptor status in breast cancer. | Involved in EMT in pancreatic cancer via the SIRT1 pathway.  Targets PTEN and kRAS.  Negatively correlated to KRAS. | [[27](#_ENREF_27), [36](#_ENREF_36), [49](#_ENREF_49), [183](#_ENREF_183), [196](#_ENREF_196), [203](#_ENREF_203), [219](#_ENREF_219), [263](#_ENREF_263), [337-340](#_ENREF_337)] |
| miR-222-3p | ↑ in PC vs. HS.  ↑ in PC vs. HS+CP.  Part of diagnostic index II, V. | ↑ in PC.  Prognostic in PDAC and A-AC.  Related to Ki67.  LASSO classifier.  ↑ in PanIN. | ↓ in prostate and thyroid cancer and cholangiocarcinoma.  ↑ in HCC, breast cancer and thyroid cancer.  Prognostic in breast cancer.  Associated with tumor-stage and prognosis in gastric cancer. | NF-κB induces miR-222  Targets Cyklin dependent kinase 4 (CDK4).  kRAS regulates miR-222 in vitro  Targets Dickkopf-2 gene (DDK) and thereby activates the Wnt/β-catenin signaling pathway => promoting tumorigenesis (in glioma)  Upregulation of miR-222 => increase of metalloproteases and repression of cell cycle inhibitors  Modulates multi-drug resistance in CRC by downregulating ADAM-17 (metalloprotease/desintegrin)  inhibits the pro-apoptotic PUMA gene in gliomas.  Directly targets p27 and p57 mRNAs.  Targets PTEN and TIMP3, induce TRAIL resistance, and  enhance cellular migration; MET oncogene activates  miR-222/221 through the c-Jun transcription  Repress DICER in ERα-negative breast cancers  Directly regulate apoptosis by targeting PUMA in Glioblastoma  miR-222 targets c-Kit, controlling the ability of endothelial  cells to form new capillaries factor  Promotes EMT in breast cancer via targeting TRPS1  Regulates the IFNα-STAT pathway | [[1](#_ENREF_1), [27](#_ENREF_27), [34](#_ENREF_34), [35](#_ENREF_35), [37](#_ENREF_37), [143](#_ENREF_143), [187](#_ENREF_187), [203](#_ENREF_203), [220](#_ENREF_220), [236](#_ENREF_236), [243](#_ENREF_243), [341-355](#_ENREF_341)] |
| miR-222-5p (222*) |  | ↑ in PC  Related to poor prognosis in PDAC.  LASSO classifier.  Related to Ki67.  ↑ in PanIN. | ↑ in thyroid cancer.  ↓ in prostate cancer and thyroid cancer.  Associated with tumor-stage and prognosis in gastric cancer. | NF-κB induces miR-222  Targets Cyklin dependent kinase 4 (CDK4)  kRAS regulates miR-222 in vitro  Targets Dickkopf-2 gene (DDK) and thereby activates the Wnt/β-catenin signaling pathway => promoting tumorigenesis (in glioma)  Upregulation of miR-222 => increase of metalloproteases and repression of cell cycle inhibitors  Modulates multi-drug resistance in CRC by downregulating ADAM-17 (metalloprotease/desintegrin)  Inhibits the pro-apoptotic PUMA gene in gliomas.  Directly targets p27 and p57 mRNAs.  Targets PTEN and TIMP3, induce TRAIL resistance, and  enhance cellular migration  MET oncogene activates  miR-222/221 through the c-Jun transcription factor.  Promotes EMT in breast cancer via targeting TRPS 1  Repress DICER in ERα-negative breast cancers  Directly regulate apoptosis by targeting PUMA in Glioblastoma  miR-222 targets c-Kit, controlling the ability of endothelial  cells to form new capillaries  Regulates the IFNα-STAT pathway | [[1](#_ENREF_1), [27](#_ENREF_27), [34](#_ENREF_34), [35](#_ENREF_35), [37](#_ENREF_37), [143](#_ENREF_143), [187](#_ENREF_187), [203](#_ENREF_203), [220](#_ENREF_220), [236](#_ENREF_236), [243](#_ENREF_243), [341-353](#_ENREF_341), [355](#_ENREF_355)] |
| miR-375 | ↓ in PC vs. HS.  ↓ in PC vs. HS+CP.  Part of diagnostic index I, IV. | Diagnostic. ↓ in PC.  Prognostic.  Able to differentiate between pancreatobiliary and intestinal subtypes in periampullary cancers. | ↑ in prostate cancer.  ↓ in esophageal, gastric and breast, lung, colorectal, HCC, cervical, and head and neck cancer.  Prognostic in lung cancer, esophageal, head and neck carcinoma.  Maintains normal pancreatic alpha- and beta cell mass and is upregulated in pancreas from patients with type 2 diabetes. | Disco-interacting protein 2 homolog c; KIAA1199 (colon cancer secreted protein 1), lactate dehydrogenase B, QKI, SEC23A, SLC7A11  Targets YAP oncogene in HCC  Inhibits cell proliferation by targeting JAK2  Targets SEC23A in prostate cancer  Inhibits oncogene AEG-1/MTDH in head and neck carcinoma.  Targets 3-phosphoinositide-dependent protein kinase-1 (PDK1) in pancreatic and esophageal cancer  Targets IGF1R in esophageal cancer.  Inhibits pancreatic cancer cell proliferation in vitro.  Suppresses cell proliferation in colorectal cancer by targeting the PI3K/Akt pathway  Tumorsuppressive in gastric cancer by targeting ERBB2 | [[26](#_ENREF_26), [27](#_ENREF_27), [32](#_ENREF_32), [103](#_ENREF_103), [107](#_ENREF_107), [215](#_ENREF_215), [219](#_ENREF_219), [356-373](#_ENREF_356)] |
| miR-411-5p | Not significant in PC vs. HS. | 2-miR-test. | ↓ in breast cancer, glioblastoma, ovarian cancer.  Prognostic in lung cancer. |  | [[27](#_ENREF_27), [374-377](#_ENREF_374)] |
| miR-431-5p (431) |  | Prognostic. | ↑ in benign Schwannomas.  Deregulated in serum in colorectal cancer. | Interferon-β decreases miR-431 expression and upregulates SOCS6 expression, and consequently inhibit cell proliferation by suppressing the JAK-STAT signaling pathway in glioblastoma | [[1](#_ENREF_1), [378-380](#_ENREF_378)] |
| miR-450b-5p | Prognostic in AAC.  Part of index VI. | Prognostic (2-year follow up). | ↑ in lung after cigarette smoking. | Suppressed by Smad3/4 in rhabdomyosarcoma. | [[1](#_ENREF_1), [381](#_ENREF_381), [382](#_ENREF_382)] |
| miR-451a | ↑ in PC vs. HS.  ↑ in PC vs. HS+CP.  Part of diagnostic index I, II, III, IV, V, VI. | ↑ in PC.  LASSO classifier.  Detected in FNAC from PC.  ↓ in pancreatic carcinoma cyst fluid | Widely dysregulated (mostly downregulated) in human malignancies including lung, gastric, head and neck, colorectal, and breast cancers, gliomas and leukemias.  Potential serum biomarker in breast and renal cell cancer. | Considered a tumor suppressor.  Targets (inhibits) Ras-related protein 14 (RAB14), PI3K/AKT-pathway and C-MYC.  Induces EMT via c-Myc in lung cancer  Targets the oncogene macrophage migration inhibitory factor (MIF) in several GI cancers.  Inhibit AMPK to activate mTORC1, which mediates FSCN1 expression and cancer cell progression in CRC  Directly targets COX2 (in vitro) | [[27](#_ENREF_27), [383-392](#_ENREF_383)] |
| miR-490-3p |  | LASSO classifier. | ↓ in bladder cancer and lung cancer (cell lines), colorectal cancer, HCC, gastric cancer. | Modulates cell growth and EMT of hepatocellular carcinoma cells by targeting endoplasmic reticulum-Golgi intermediate compartment protein 3 (ERGIC3).  Tumor suppressor in bladder cancer through targeting c-Fos  Tumor suppressor in lung cancer (cell lines) via targeting CCND1 (Involved in G1 to S phase transition).  Tumor suppressor in gastric cancer via targeting SMARCD1. | [[27](#_ENREF_27), [393-398](#_ENREF_393)] |
| miR-492 | ↑ in PC vs. HS+CP. | LASSO classifier.  Downregulated in serum from patients with PC.  Elevated in pancreatic juice. | Dysregulated in CRC, hepatoblastoma, retinoblastoma and HCC. | Anti-angiogenic activity in endothelial cells.  implicated in the regulation of HCC progression through PTEN and AKT pathway | [[27](#_ENREF_27), [268](#_ENREF_268), [301](#_ENREF_301), [399-403](#_ENREF_399)] |
| miR-509-5p |  | LASSO classifier. | ↑ in breast cancer. | miR-509 suppresses brain metastasis of breast cancer cells by modulating RhoC and TNF-α | [[27](#_ENREF_27), [404](#_ENREF_404)] |
| miR-571 |  | LASSO classifier. |  |  | [[27](#_ENREF_27)] |
| miR-614 | Not significant in PC vs. HS.  ↑ in PC vs. HS+CP | LASSO classifier.  2-miR-test. |  |  | [[27](#_ENREF_27)] |
| miR-622 | ↑ in PC vs. HS.  ↑ in PC vs. HS+CP.  Part of diagnostic index II, V, VI. | LASSO classifier. | ↓ promotes metastasis in gastric cancer. | Tumor suppression by targeting kRAS (cell lines)  Targets ING1 gene in gastric cancer.  miR-622 suppresses proliferation, invasion and migration by directly targeting activating transcription factor 2 in glioma cells | [[27](#_ENREF_27), [405-407](#_ENREF_405)] |
| miR-625-5p |  | Prognostic in A-AC (low).  ↓ in PanIN. | ↓ in HCC.  ↓ and negatively correlated with lymph node metastasis in gastric cancer and promotes invasion.  ↓ in serum from lung cancer patients (miR-625*).  ↓ in response to hypoxia in sarcoma cell lines.  High miR-625-3p is associated with poor response to first-line oxaliplatin based chemotherapy of metastatic CRC. In contrast low expression has been described as a negative prognostic marker independently of treatment in CRC. | Targets ILK and Sox2 in gastric cancer.  miR-625 suppresses tumor migration and invasion by targeting IGF2BP1/PTEN in HCC. | [[1](#_ENREF_1), [203](#_ENREF_203), [408-415](#_ENREF_408)] |
| miR-675-5p |  | Prognostic (high). | ↑ in gastric cancer.  ↓ in metastatic prostate cancer compared to non-metastatic (cell lines).  Involved in cartilage development and homeostasis. | Derived from the oncofetal H19 lncRNA.  Directly targets the tumor suppressor Rb in CRC.  Targets the IGF1 receptor gene.  miR-675 mediates downregulation of Twist1 and Rb in AFP-secreting hepatocellular carcinoma.  Directly targets the tumor suppressor Runt Domain Transcription Factor1 (RUNX1) in Gastric Cancer.  Targets transforming growth factor β induced protein (TGFBI) in prostate cancer and thereby act as a tumor suppressor.  Antagonizes the let-7 family.  Promotes tumor metastasis via a positive feedback loop between Slug and H19/miR-675 that regulates E-cadherin expression. | [[1](#_ENREF_1), [416-423](#_ENREF_416)] |
| miR-769-5p | Part of diagnostic index VI. | Prognostic. |  | MicroRNA-769-3p down-regulates NDRG1 and enhances apoptosis in MCF-7 cells during reoxygenation. | [[1](#_ENREF_1), [424](#_ENREF_424)] |
| miR-939 |  | LASSO classifier. | ↓ in lung cancer. |  | [[27](#_ENREF_27), [425](#_ENREF_425)] |
| miR-944 | ↑ in PC vs. HS+CP. | Diagnostic.  Prognostic. | ↑ in cervical cancer.  High expression in primary CRC tumor is negatively related to recurrence. | miR-944 is located in the intron of the Tumor protein p63 gene.  Targets the tumor suppressor SOCS4 in NSCLC and related to growth, proliferation and invasion.  Targets HECT domain ligase W2 and S100P binding protein in cervical cancer cells. | [[1](#_ENREF_1), [426-428](#_ENREF_426)] |

References

1. Schultz NA, Andersen KK, Roslind A, Willenbrock H, Wojdemann M, Johansen JS. Prognostic MicroRNAs in Cancer Tissue from Patients Operated for Pancreatic Cancer-Five MicroRNAs in a Prognostic Index. World J Surg 2012.

2. West JA, Viswanathan SR, Yabuuchi A, Cunniff K, Takeuchi A, Park IH, Sero JE, Zhu H, Perez-Atayde A, Frazier AL *et al*. A role for Lin28 in primordial germ-cell development and germ-cell malignancy. Nature 2009, 460(7257):909-913.

3. Nadiminty N, Tummala R, Lou W, Zhu Y, Shi XB, Zou JX, Chen H, Zhang J, Chen X, Luo J *et al*. MicroRNA let-7c is downregulated in prostate cancer and suppresses prostate cancer growth. PLoS One 2012, 7(3):e32832.

4. Lu L, Katsaros D, Shaverdashvili K, Qian B, Wu Y, de la Longrais IA, Preti M, Menato G, Yu H. Pluripotent factor lin-28 and its homologue lin-28b in epithelial ovarian cancer and their associations with disease outcomes and expression of let-7a and IGF-II. Eur J Cancer 2009, 45(12):2212-2218.

5. Hamano R, Miyata H, Yamasaki M, Sugimura K, Tanaka K, Kurokawa Y, Nakajima K, Takiguchi S, Fujiwara Y, Mori M *et al*. High expression of Lin28 is associated with tumour aggressiveness and poor prognosis of patients in oesophagus cancer. Br J Cancer 2012, 106(8):1415-1423.

6. Pan L, Gong Z, Zhong Z, Dong Z, Liu Q, Le Y, Guo J. Lin-28 reactivation is required for let-7 repression and proliferation in human small cell lung cancer cells. Mol Cell Biochem 2011, 355(1-2):257-263.

7. Wang S, Tang Y, Cui H, Zhao X, Luo X, Pan W, Huang X, Shen N. Let-7/miR-98 regulate Fas and Fas-mediated apoptosis. Genes Immun 2011, 12(2):149-154.

8. Heo I, Joo C, Cho J, Ha M, Han J, Kim VN. Lin28 mediates the terminal uridylation of let-7 precursor MicroRNA. Mol Cell 2008, 32(2):276-284.

9. Piskounova E, Polytarchou C, Thornton JE, LaPierre RJ, Pothoulakis C, Hagan JP, Iliopoulos D, Gregory RI. Lin28A and Lin28B inhibit let-7 microRNA biogenesis by distinct mechanisms. Cell 2011, 147(5):1066-1079.

10. Whelan JT, Hollis SE, Cha DS, Asch AS, Lee MH. Post-transcriptional regulation of the Ras-ERK/MAPK signaling pathway. J Cell Physiol 2012, 227(3):1235-1241.

11. Ma X, Becker Buscaglia LE, Barker JR, Li Y. MicroRNAs in NF-kappaB signaling. J Mol Cell Biol 2011, 3(3):159-166.

12. Weingart MF, Roth JJ, Hutt-Cabezas M, Busse TM, Kaur H, Price A, Maynard R, Rubens J, Taylor I, Mao XG *et al*. Disrupting LIN28 in atypical teratoid rhabdoid tumors reveals the importance of the mitogen activated protein kinase pathway as a therapeutic target. Oncotarget 2014.

13. Kang W, Tong JH, Lung RW, Dong Y, Yang W, Pan Y, Lau KM, Yu J, Cheng AS, To KF. let-7b/g silencing activates AKT signaling to promote gastric carcinogenesis. J Transl Med 2014, 12:281.

14. Gowrishankar B, Ibragimova I, Zhou Y, Slifker MJ, Devarajan K, Al-Saleem T, Uzzo RG, Cairns P. MicroRNA expression signatures of stage, grade, and progression in clear cell RCC. Cancer Biol Ther 2014, 15(3):329-341.

15. Chen KJ, Hou Y, Wang K, Li J, Xia Y, Yang XY, Lv G, Xing XL, Shen F. Reexpression of Let-7g microRNA inhibits the proliferation and migration via K-Ras/HMGA2/snail axis in hepatocellular carcinoma. Biomed Res Int 2014, 2014:742417.

16. Hrdlickova R, Nehyba J, Bargmann W, Bose HR, Jr. Multiple tumor suppressor microRNAs regulate telomerase and TCF7, an important transcriptional regulator of the Wnt pathway. PLoS One 2014, 9(2):e86990.

17. Hu X, Guo J, Zheng L, Li C, Zheng TM, Tanyi JL, Liang S, Benedetto C, Mitidieri M, Katsaros D *et al*. The heterochronic microRNA let-7 inhibits cell motility by regulating the genes in the actin cytoskeleton pathway in breast cancer. Mol Cancer Res 2013, 11(3):240-250.

18. Zhao L, Sun Y, Hou Y, Peng Q, Wang L, Luo H, Tang X, Zeng Z, Liu M. MiRNA expression analysis of cancer-associated fibroblasts and normal fibroblasts in breast cancer. Int J Biochem Cell Biol 2012, 44(11):2051-2059.

19. Kim CH, Kim HK, Rettig RL, Kim J, Lee ET, Aprelikova O, Choi IJ, Munroe DJ, Green JE. miRNA signature associated with outcome of gastric cancer patients following chemotherapy. BMC Med Genomics 2011, 4:79.

20. Lightfoot HL, Bugaut A, Armisen J, Lehrbach NJ, Miska EA, Balasubramanian S. A LIN28-dependent structural change in pre-let-7g directly inhibits dicer processing. Biochemistry 2011, 50(35):7514-7521.

21. Bianchini L, Saada E, Gjernes E, Marty M, Haudebourg J, Birtwisle-Peyrottes I, Keslair F, Chignon-Sicard B, Chamorey E, Pedeutour F. Let-7 microRNA and HMGA2 levels of expression are not inversely linked in adipocytic tumors: analysis of 56 lipomas and liposarcomas with molecular cytogenetic data. Genes Chromosomes Cancer 2011, 50(6):442-455.

22. Arora H, Qureshi R, Jin S, Park AK, Park WY. miR-9 and let-7g enhance the sensitivity to ionizing radiation by suppression of NFkappaB1. Exp Mol Med 2011, 43(5):298-304.

23. Li T, Chen JX, Fu XP, Yang S, Zhang Z, Chen Kh H, Li Y. microRNA expression profiling of nasopharyngeal carcinoma. Oncol Rep 2011, 25(5):1353-1363.

24. Lan FF, Wang H, Chen YC, Chan CY, Ng SS, Li K, Xie D, He ML, Lin MC, Kung HF. Hsa-let-7g inhibits proliferation of hepatocellular carcinoma cells by downregulation of c-Myc and upregulation of p16(INK4A). Int J Cancer 2011, 128(2):319-331.

25. Ueda T, Volinia S, Okumura H, Shimizu M, Taccioli C, Rossi S, Alder H, Liu CG, Oue N, Yasui W *et al*. Relation between microRNA expression and progression and prognosis of gastric cancer: a microRNA expression analysis. Lancet Oncol 2010, 11(2):136-146.

26. Bloomston M, Frankel WL, Petrocca F, Volinia S, Alder H, Hagan JP, Liu CG, Bhatt D, Taccioli C, Croce CM. MicroRNA expression patterns to differentiate pancreatic adenocarcinoma from normal pancreas and chronic pancreatitis. JAMA 2007, 297(17):1901-1908.

27. Schultz NA, Werner J, Willenbrock H, Roslind A, Giese N, Horn T, Wojdemann M, Johansen JS. MicroRNA expression profiles associated with pancreatic adenocarcinoma and ampullary adenocarcinoma. Mod Pathol 2012.

28. Tili E, Michaille JJ, Croce CM. MicroRNAs play a central role in molecular dysfunctions linking inflammation with cancer. Immunol Rev 2013, 253(1):167-184.

29. Dillhoff M, Liu J, Frankel W, Croce C, Bloomston M. MicroRNA-21 is overexpressed in pancreatic cancer and a potential predictor of survival. J Gastrointest Surg 2008, 12(12):2171-2176.

30. Jamieson NB, Morran DC, Morton JP, Ali A, Dickson EJ, Carter CR, Sansom OJ, Evans TR, McKay CJ, Oien KA. MicroRNA molecular profiles associated with diagnosis, clinicopathologic criteria, and overall survival in patients with resectable pancreatic ductal adenocarcinoma. Clin Cancer Res 2012, 18(2):534-545.

31. Tavano F, di Mola FF, Piepoli A, Panza A, Copetti M, Burbaci FP, Latiano T, Pellegrini F, Maiello E, Andriulli A *et al*. Changes in miR-143 and miR-21 expression and clinicopathological correlations in pancreatic cancers. Pancreas 2012, 41(8):1280-1284.

32. Gu J, Wang Y, Wu X. MicroRNA in the pathogenesis and prognosis of esophageal cancer. Curr Pharm Des 2013, 19(7):1292-1300.

33. Gocze K, Gombos K, Juhasz K, Kovacs K, Kajtar B, Benczik M, Gocze P, Patczai B, Arany I, Ember I. Unique MicroRNA Expression Profiles in Cervical Cancer. Anticancer Res 2013, 33(6):2561-2567.

34. Karakatsanis A, Papaconstantinou I, Gazouli M, Lyberopoulou A, Polymeneas G, Voros D. Expression of microRNAs, miR-21, miR-31, miR-122, miR-145, miR-146a, miR-200c, miR-221, miR-222, and miR-223 in patients with hepatocellular carcinoma or intrahepatic cholangiocarcinoma and its prognostic significance. Mol Carcinog 2013, 52(4):297-303.

35. Papaconstantinou IG, Manta A, Gazouli M, Lyberopoulou A, Lykoudis PM, Polymeneas G, Voros D. Expression of microRNAs in patients with pancreatic cancer and its prognostic significance. Pancreas 2013, 42(1):67-71.

36. Xue Y, Abou Tayoun AN, Abo KM, Pipas JM, Gordon SR, Gardner TB, Barth RJ, Jr., Suriawinata AA, Tsongalis GJ. MicroRNAs as diagnostic markers for pancreatic ductal adenocarcinoma and its precursor, pancreatic intraepithelial neoplasm. Cancer Genet 2013, 206(6):217-221.

37. Di Leva G, Garofalo M, Croce CM. MicroRNAs in cancer. Annu Rev Pathol 2014, 9:287-314.

38. Moriyama T, Ohuchida K, Mizumoto K, Yu J, Sato N, Nabae T, Takahata S, Toma H, Nagai E, Tanaka M. MicroRNA-21 modulates biological functions of pancreatic cancer cells including their proliferation, invasion, and chemoresistance. Mol Cancer Ther 2009, 8(5):1067-1074.

39. Kadera BE, Li L, Toste PA, Wu N, Adams C, Dawson DW, Donahue TR. MicroRNA-21 in pancreatic ductal adenocarcinoma tumor-associated fibroblasts promotes metastasis. PLoS One 2013, 8(8):e71978.

40. Nagao Y, Hisaoka M, Matsuyama A, Kanemitsu S, Hamada T, Fukuyama T, Nakano R, Uchiyama A, Kawamoto M, Yamaguchi K *et al*. Association of microRNA-21 expression with its targets, PDCD4 and TIMP3, in pancreatic ductal adenocarcinoma. Mod Pathol 2012, 25(1):112-121.

41. Bera A, VenkataSubbaRao K, Manoharan MS, Hill P, Freeman JW. A miRNA signature of chemoresistant mesenchymal phenotype identifies novel molecular targets associated with advanced pancreatic cancer. PLoS One 2014, 9(9):e106343.

42. Donahue TR, Nguyen AH, Moughan J, Li L, Tatishchev S, Toste P, Farrell JJ. Stromal microRNA-21 levels predict response to 5-fluorouracil in patients with pancreatic cancer. J Surg Oncol 2014, 110(8):952-959.

43. Passadouro M, Pedroso de Lima MC, Faneca H. MicroRNA modulation combined with sunitinib as a novel therapeutic strategy for pancreatic cancer. Int J Nanomedicine 2014, 9:3203-3217.

44. Frampton AE, Giovannetti E, Jamieson NB, Krell J, Gall TM, Stebbing J, Jiao LR, Castellano L. A microRNA meta-signature for pancreatic ductal adenocarcinoma. Expert Rev Mol Diagn 2014, 14(3):267-271.

45. Farrell JJ, Toste P, Wu N, Li L, Wong J, Malkhassian D, Tran LM, Wu X, Li X, Dawson D *et al*. Endoscopically acquired pancreatic cyst fluid microRNA 21 and 221 are associated with invasive cancer. Am J Gastroenterol 2013, 108(8):1352-1359.

46. Mace TA, Collins AL, Wojcik SE, Croce CM, Lesinski GB, Bloomston M. Hypoxia induces the overexpression of microRNA-21 in pancreatic cancer cells. J Surg Res 2013, 184(2):855-860.

47. Panarelli NC, Chen YT, Zhou XK, Kitabayashi N, Yantiss RK. MicroRNA expression aids the preoperative diagnosis of pancreatic ductal adenocarcinoma. Pancreas 2012, 41(5):685-690.

48. Jiao LR, Frampton AE, Jacob J, Pellegrino L, Krell J, Giamas G, Tsim N, Vlavianos P, Cohen P, Ahmad R *et al*. MicroRNAs targeting oncogenes are down-regulated in pancreatic malignant transformation from benign tumors. PLoS One 2012, 7(2):e32068.

49. Ali S, Banerjee S, Logna F, Bao B, Philip PA, Korc M, Sarkar FH. Inactivation of Ink4a/Arf leads to deregulated expression of miRNAs in K-Ras transgenic mouse model of pancreatic cancer. J Cell Physiol 2012, 227(10):3373-3380.

50. Bao B, Wang Z, Ali S, Kong D, Li Y, Ahmad A, Banerjee S, Azmi AS, Miele L, Sarkar FH. Notch-1 induces epithelial-mesenchymal transition consistent with cancer stem cell phenotype in pancreatic cancer cells. Cancer Lett 2011, 307(1):26-36.

51. Dong J, Zhao YP, Zhou L, Zhang TP, Chen G. Bcl-2 upregulation induced by miR-21 via a direct interaction is associated with apoptosis and chemoresistance in MIA PaCa-2 pancreatic cancer cells. Arch Med Res 2011, 42(1):8-14.

52. Bhatti I, Lee A, James V, Hall RI, Lund JN, Tufarelli C, Lobo DN, Larvin M. Knockdown of microRNA-21 inhibits proliferation and increases cell death by targeting programmed cell death 4 (PDCD4) in pancreatic ductal adenocarcinoma. J Gastrointest Surg 2011, 15(1):199-208.

53. Hwang JH, Voortman J, Giovannetti E, Steinberg SM, Leon LG, Kim YT, Funel N, Park JK, Kim MA, Kang GH *et al*. Identification of microRNA-21 as a biomarker for chemoresistance and clinical outcome following adjuvant therapy in resectable pancreatic cancer. PLoS One 2010, 5(5):e10630.

54. Giovannetti E, Funel N, Peters GJ, Del Chiaro M, Erozenci LA, Vasile E, Leon LG, Pollina LE, Groen A, Falcone A *et al*. MicroRNA-21 in pancreatic cancer: correlation with clinical outcome and pharmacologic aspects underlying its role in the modulation of gemcitabine activity. Cancer Res 2010, 70(11):4528-4538.

55. Ryu JK, Hong SM, Karikari CA, Hruban RH, Goggins MG, Maitra A. Aberrant MicroRNA-155 expression is an early event in the multistep progression of pancreatic adenocarcinoma. Pancreatology 2010, 10(1):66-73.

56. Lee EJ, Gusev Y, Jiang J, Nuovo GJ, Lerner MR, Frankel WL, Morgan DL, Postier RG, Brackett DJ, Schmittgen TD. Expression profiling identifies microRNA signature in pancreatic cancer. Int J Cancer 2007, 120(5):1046-1054.

57. Roldo C, Missiaglia E, Hagan JP, Falconi M, Capelli P, Bersani S, Calin GA, Volinia S, Liu CG, Scarpa A *et al*. MicroRNA expression abnormalities in pancreatic endocrine and acinar tumors are associated with distinctive pathologic features and clinical behavior. J Clin Oncol 2006, 24(29):4677-4684.

58. Jahid S, Sun J, Edwards RA, Dizon D, Panarelli NC, Milsom JW, Sikandar SS, Gumus ZH, Lipkin SM. miR-23a promotes the transition from indolent to invasive colorectal cancer. Cancer Discov 2012, 2(6):540-553.

59. Chen X, Ba Y, Ma L, Cai X, Yin Y, Wang K, Guo J, Zhang Y, Chen J, Guo X *et al*. Characterization of microRNAs in serum: a novel class of biomarkers for diagnosis of cancer and other diseases. Cell Res 2008, 18(10):997-1006.

60. Chhabra R, Dubey R, Saini N. Cooperative and individualistic functions of the microRNAs in the miR-23a~27a~24-2 cluster and its implication in human diseases. Mol Cancer 2010, 9:232.

61. Kozaki K, Imoto I, Mogi S, Omura K, Inazawa J. Exploration of tumor-suppressive microRNAs silenced by DNA hypermethylation in oral cancer. Cancer Res 2008, 68(7):2094-2105.

62. Rogler CE, Levoci L, Ader T, Massimi A, Tchaikovskaya T, Norel R, Rogler LE. MicroRNA-23b cluster microRNAs regulate transforming growth factor-beta/bone morphogenetic protein signaling and liver stem cell differentiation by targeting Smads. Hepatology 2009, 50(2):575-584.

63. Willert K, Jones KA. Wnt signaling: is the party in the nucleus? Genes Dev 2006, 20(11):1394-1404.

64. Zhu LH, Liu T, Tang H, Tian RQ, Su C, Liu M, Li X. MicroRNA-23a promotes the growth of gastric adenocarcinoma cell line MGC803 and downregulates interleukin-6 receptor. FEBS J 2010, 277(18):3726-3734.

65. Liu N, Sun YY, Zhang XW, Chen S, Wang Y, Zhang ZX, Song SW, Qiu GB, Fu WN. Oncogenic miR-23a in Pancreatic Ductal Adenocarcinogenesis Via Inhibiting APAF1. Dig Dis Sci 2015.

66. Cai S, Chen R, Li X, Cai Y, Ye Z, Li S, Li J, Huang H, Peng S, Wang J *et al*. Downregulation of microRNA-23a suppresses prostate cancer metastasis by targeting the PAK6-LIMK1 signaling pathway. Oncotarget 2015.

67. Listing H, Mardin WA, Wohlfromm S, Mees ST, Haier J. MiR-23a/-24-induced gene silencing results in mesothelial cell integration of pancreatic cancer. Br J Cancer 2015, 112(1):131-139.

68. Yong FL, Wang CW, Roslani AC, Law CW. The involvement of miR-23a/APAF1 regulation axis in colorectal cancer. Int J Mol Sci 2014, 15(7):11713-11729.

69. Wang N, Zhu M, Tsao SW, Man K, Zhang Z, Feng Y. MiR-23a-mediated inhibition of topoisomerase 1 expression potentiates cell response to etoposide in human hepatocellular carcinoma. Mol Cancer 2013, 12(1):119.

70. Li X, Liu X, Xu W, Zhou P, Gao P, Jiang S, Lobie PE, Zhu T. c-MYC-regulated miR-23a/24-2/27a cluster promotes mammary carcinoma cell invasion and hepatic metastasis by targeting Sprouty2. J Biol Chem 2013, 288(25):18121-18133.

71. Cao M, Seike M, Soeno C, Mizutani H, Kitamura K, Minegishi Y, Noro R, Yoshimura A, Cai L, Gemma A. MiR-23a regulates TGF-beta-induced epithelial-mesenchymal transition by targeting E-cadherin in lung cancer cells. Int J Oncol 2012, 41(3):869-875.

72. Vega AB, Pericay C, Moya I, Ferrer A, Dotor E, Pisa A, Casalots A, Serra-Aracil X, Oliva JC, Ruiz A *et al*. microRNA expression profile in stage III colorectal cancer: circulating miR-18a and miR-29a as promising biomarkers. Oncol Rep 2013, 30(1):320-326.

73. Nagano H, Tomimaru Y, Eguchi H, Hama N, Wada H, Kawamoto K, Kobayashi S, Mori M, Doki Y. MicroRNA-29a induces resistance to gemcitabine through the Wnt/beta-catenin signaling pathway in pancreatic cancer cells. Int J Oncol 2013, 43(4):1066-1072.

74. Kogure T, Kondo Y, Kakazu E, Ninomiya M, Kimura O, Shimosegawa T. Involvement of miRNA-29a in epigenetic regulation of transforming growth factor-beta-induced epithelial-mesenchymal transition in hepatocellular carcinoma. Hepatol Res 2013.

75. Luo X, Stock C, Burwinkel B, Brenner H. Identification and evaluation of plasma MicroRNAs for early detection of colorectal cancer. PLoS One 2013, 8(5):e62880.

76. Kuo TY, Hsi E, Yang IP, Tsai PC, Wang JY, Juo SH. Computational analysis of mRNA expression profiles identifies microRNA-29a/c as predictor of colorectal cancer early recurrence. PLoS One 2012, 7(2):e31587.

77. Braconi C, Kogure T, Valeri N, Huang N, Nuovo G, Costinean S, Negrini M, Miotto E, Croce CM, Patel T. microRNA-29 can regulate expression of the long non-coding RNA gene MEG3 in hepatocellular cancer. Oncogene 2011, 30(47):4750-4756.

78. Zhang H, Cheng Y, Jia C, Yu S, Xiao Y, Chen J. MicroRNA-29s could target AKT2 to inhibit gastric cancer cells invasion ability. Med Oncol 2015, 32(1):342.

79. Han HS, Son SM, Yun J, Jo YN, Lee OJ. MicroRNA-29a suppresses the growth, migration, and invasion of lung adenocarcinoma cells by targeting carcinoembryonic antigen-related cell adhesion molecule 6. FEBS Lett 2014, 588(20):3744-3750.

80. Nishikawa R, Goto Y, Kojima S, Enokida H, Chiyomaru T, Kinoshita T, Sakamoto S, Fuse M, Nakagawa M, Naya Y *et al*. Tumor-suppressive microRNA-29s inhibit cancer cell migration and invasion via targeting LAMC1 in prostate cancer. Int J Oncol 2014, 45(1):401-410.

81. Tang W, Zhu Y, Gao J, Fu J, Liu C, Liu Y, Song C, Zhu S, Leng Y, Wang G *et al*. MicroRNA-29a promotes colorectal cancer metastasis by regulating matrix metalloproteinase 2 and E-cadherin via KLF4. Br J Cancer 2014, 110(2):450-458.

82. Wu Z, Huang X, Huang X, Zou Q, Guo Y. The inhibitory role of Mir-29 in growth of breast cancer cells. J Exp Clin Cancer Res 2013, 32:98.

83. Yamamoto N, Kinoshita T, Nohata N, Yoshino H, Itesako T, Fujimura L, Mitsuhashi A, Usui H, Enokida H, Nakagawa M *et al*. Tumor-suppressive microRNA-29a inhibits cancer cell migration and invasion via targeting HSP47 in cervical squamous cell carcinoma. Int J Oncol 2013, 43(6):1855-1863.

84. Zhu XC, Dong QZ, Zhang XF, Deng B, Jia HL, Ye QH, Qin LX, Wu XZ. microRNA-29a suppresses cell proliferation by targeting SPARC in hepatocellular carcinoma. Int J Mol Med 2012, 30(6):1321-1326.

85. Cui Y, Su WY, Xing J, Wang YC, Wang P, Chen XY, Shen ZY, Cao H, Lu YY, Fang JY. MiR-29a inhibits cell proliferation and induces cell cycle arrest through the downregulation of p42.3 in human gastric cancer. PLoS One 2011, 6(10):e25872.

86. Chen J, Li Q, An Y, Lv N, Xue X, Wei J, Jiang K, Wu J, Gao W, Qian Z *et al*. CEACAM6 induces epithelial-mesenchymal transition and mediates invasion and metastasis in pancreatic cancer. Int J Oncol 2013, 43(3):877-885.

87. Zhong Z, Dong Z, Yang L, Chen X, Gong Z. MicroRNA-31-5p modulates cell cycle by targeting human mutL homolog 1 in human cancer cells. Tumour Biol 2013, 34(3):1959-1965.

88. Motoyama K, Inoue H, Takatsuno Y, Tanaka F, Mimori K, Uetake H, Sugihara K, Mori M. Over- and under-expressed microRNAs in human colorectal cancer. Int J Oncol 2009, 34(4):1069-1075.

89. Wong QW, Lung RW, Law PT, Lai PB, Chan KY, To KF, Wong N. MicroRNA-223 is commonly repressed in hepatocellular carcinoma and potentiates expression of Stathmin1. Gastroenterology 2008, 135(1):257-269.

90. Liu CJ, Kao SY, Tu HF, Tsai MM, Chang KW, Lin SC. Increase of microRNA miR-31 level in plasma could be a potential marker of oral cancer. Oral Dis 2010, 16(4):360-364.

91. Liu CJ, Tsai MM, Hung PS, Kao SY, Liu TY, Wu KJ, Chiou SH, Lin SC, Chang KW. miR-31 ablates expression of the HIF regulatory factor FIH to activate the HIF pathway in head and neck carcinoma. Cancer Res 2010, 70(4):1635-1644.

92. Liu X, Sempere LF, Ouyang H, Memoli VA, Andrew AS, Luo Y, Demidenko E, Korc M, Shi W, Preis M *et al*. MicroRNA-31 functions as an oncogenic microRNA in mouse and human lung cancer cells by repressing specific tumor suppressors. J Clin Invest 2010, 120(4):1298-1309.

93. Schaefer A, Jung M, Mollenkopf HJ, Wagner I, Stephan C, Jentzmik F, Miller K, Lein M, Kristiansen G, Jung K. Diagnostic and prognostic implications of microRNA profiling in prostate carcinoma. Int J Cancer 2010, 126(5):1166-1176.

94. Zhang Y, Guo J, Li D, Xiao B, Miao Y, Jiang Z, Zhuo H. Down-regulation of miR-31 expression in gastric cancer tissues and its clinical significance. Med Oncol 2010, 27(3):685-689.

95. Valastyan S, Reinhardt F, Benaich N, Calogrias D, Szasz AM, Wang ZC, Brock JE, Richardson AL, Weinberg RA. A pleiotropically acting microRNA, miR-31, inhibits breast cancer metastasis. Cell 2009, 137(6):1032-1046.

96. Creighton CJ, Fountain MD, Yu Z, Nagaraja AK, Zhu H, Khan M, Olokpa E, Zariff A, Gunaratne PH, Matzuk MM *et al*. Molecular profiling uncovers a p53-associated role for microRNA-31 in inhibiting the proliferation of serous ovarian carcinomas and other cancers. Cancer Res 2010, 70(5):1906-1915.

97. Sun D, Yu F, Ma Y, Zhao R, Chen X, Zhu J, Zhang CY, Chen J, Zhang J. MicroRNA-31 activates the RAS pathway and functions as an oncogenic MicroRNA in human colorectal cancer by repressing RAS p21 GTPase activating protein 1 (RASA1). J Biol Chem 2013, 288(13):9508-9518.

98. Xu RS, Wu XD, Zhang SQ, Li CF, Yang L, Li DD, Zhang BG, Zhang Y, Jin JP, Zhang B. The tumor suppressor gene RhoBTB1 is a novel target of miR-31 in human colon cancer. Int J Oncol 2013, 42(2):676-682.

99. Rokah OH, Granot G, Ovcharenko A, Modai S, Pasmanik-Chor M, Toren A, Shomron N, Shpilberg O. Downregulation of miR-31, miR-155, and miR-564 in chronic myeloid leukemia cells. PLoS One 2012, 7(4):e35501.

100. Reid JF, Sokolova V, Zoni E, Lampis A, Pizzamiglio S, Bertan C, Zanutto S, Perrone F, Camerini T, Gallino G *et al*. miRNA profiling in colorectal cancer highlights miR-1 involvement in MET-dependent proliferation. Mol Cancer Res 2012, 10(4):504-515.

101. Augoff K, Das M, Bialkowska K, McCue B, Plow EF, Sossey-Alaoui K. miR-31 is a broad regulator of beta1-integrin expression and function in cancer cells. Mol Cancer Res 2011, 9(11):1500-1508.

102. Masamune A, Nakano E, Hamada S, Takikawa T, Yoshida N, Shimosegawa T. Alteration of the microRNA expression profile during the activation of pancreatic stellate cells. Scand J Gastroenterol 2014, 49(3):323-331.

103. Kalluri Sai Shiva UM, Kuruva MM, Mitnala S, Rupjyoti T, Guduru Venkat R, Botlagunta S, Kandagaddala R, Siddapuram SP, Sekaran A, Chemalakonda R *et al*. MicroRNA profiling in periampullary carcinoma. Pancreatology 2014, 14(1):36-47.

104. Laurila EM, Sandstrom S, Rantanen LM, Autio R, Kallioniemi A. Both inhibition and enhanced expression of miR-31 lead to reduced migration and invasion of pancreatic cancer cells. Genes Chromosomes Cancer 2012, 51(6):557-568.

105. Kim HS, Lee KS, Bae HJ, Eun JW, Shen Q, Park SJ, Shin WC, Yang HD, Park M, Park WS *et al*. MicroRNA-31 functions as a tumor suppressor by regulating cell cycle and epithelial-mesenchymal transition regulatory proteins in liver cancer. Oncotarget 2015.

106. Li T, Luo W, Liu K, Lv X, Xi T. miR-31 promotes proliferation of colon cancer cells by targeting E2F2. Biotechnol Lett 2015, 37(3):523-532.

107. Luo D, Wilson JM, Harvel N, Liu J, Pei L, Huang S, Hawthorn L, Shi H. A systematic evaluation of miRNA:mRNA interactions involved in the migration and invasion of breast cancer cells. J Transl Med 2013, 11:57.

108. Garofalo M, Jeon YJ, Nuovo GJ, Middleton J, Secchiero P, Joshi P, Alder H, Nazaryan N, Di Leva G, Romano G *et al*. MiR-34a/c-Dependent PDGFR-alpha/beta Downregulation Inhibits Tumorigenesis and Enhances TRAIL-Induced Apoptosis in Lung Cancer. PLoS One 2013, 8(6):e67581.

109. Yang S, Li Y, Gao J, Zhang T, Li S, Luo A, Chen H, Ding F, Wang X, Liu Z. MicroRNA-34 suppresses breast cancer invasion and metastasis by directly targeting Fra-1. Oncogene 2012.

110. Heinemann A, Zhao F, Pechlivanis S, Eberle J, Steinle A, Diederichs S, Schadendorf D, Paschen A. Tumor suppressive microRNAs miR-34a/c control cancer cell expression of ULBP2, a stress-induced ligand of the natural killer cell receptor NKG2D. Cancer Res 2012, 72(2):460-471.

111. Hermeking H. The miR-34 family in cancer and apoptosis. Cell Death Differ 2010, 17(2):193-199.

112. Ahn YH, Gibbons DL, Chakravarti D, Creighton CJ, Rizvi ZH, Adams HP, Pertsemlidis A, Gregory PA, Wright JA, Goodall GJ *et al*. ZEB1 drives prometastatic actin cytoskeletal remodeling by downregulating miR-34a expression. J Clin Invest 2012, 122(9):3170-3183.

113. Concepcion CP, Han YC, Mu P, Bonetti C, Yao E, D'Andrea A, Vidigal JA, Maughan WP, Ogrodowski P, Ventura A. Intact p53-dependent responses in miR-34-deficient mice. PLoS Genet 2012, 8(7):e1002797.

114. Wang M, Zhang P, Li Y, Liu G, Zhou B, Zhan L, Zhou Z, Sun X. The quantitative analysis by stem-loop real-time PCR revealed the microRNA-34a, microRNA-155 and microRNA-200c overexpression in human colorectal cancer. Med Oncol 2012, 29(5):3113-3118.

115. Kim HR, Roe JS, Lee JE, Hwang IY, Cho EJ, Youn HD. A p53-inducible microRNA-34a downregulates Ras signaling by targeting IMPDH. Biochem Biophys Res Commun 2012, 418(4):682-688.

116. Yamamura S, Saini S, Majid S, Hirata H, Ueno K, Chang I, Tanaka Y, Gupta A, Dahiya R. MicroRNA-34a suppresses malignant transformation by targeting c-Myc transcriptional complexes in human renal cell carcinoma. Carcinogenesis 2012, 33(2):294-300.

117. Yamamura S, Saini S, Majid S, Hirata H, Ueno K, Deng G, Dahiya R. MicroRNA-34a modulates c-Myc transcriptional complexes to suppress malignancy in human prostate cancer cells. PLoS One 2012, 7(1):e29722.

118. Siemens H, Jackstadt R, Hunten S, Kaller M, Menssen A, Gotz U, Hermeking H. miR-34 and SNAIL form a double-negative feedback loop to regulate epithelial-mesenchymal transitions. Cell Cycle 2011, 10(24):4256-4271.

119. Garajova I, Le Large TY, Frampton AE, Rolfo C, Voortman J, Giovannetti E. Molecular mechanisms underlying the role of microRNAs in the chemoresistance of pancreatic cancer. Biomed Res Int 2014, 2014:678401.

120. Xia J, Duan Q, Ahmad A, Bao B, Banerjee S, Shi Y, Ma J, Geng J, Chen Z, Rahman KM *et al*. Genistein inhibits cell growth and induces apoptosis through up-regulation of miR-34a in pancreatic cancer cells. Curr Drug Targets 2012, 13(14):1750-1756.

121. Ikeda Y, Tanji E, Makino N, Kawata S, Furukawa T. MicroRNAs associated with mitogen-activated protein kinase in human pancreatic cancer. Mol Cancer Res 2012, 10(2):259-269.

122. Pramanik D, Campbell NR, Karikari C, Chivukula R, Kent OA, Mendell JT, Maitra A. Restitution of tumor suppressor microRNAs using a systemic nanovector inhibits pancreatic cancer growth in mice. Mol Cancer Ther 2011, 10(8):1470-1480.

123. Vogt M, Munding J, Gruner M, Liffers ST, Verdoodt B, Hauk J, Steinstraesser L, Tannapfel A, Hermeking H. Frequent concomitant inactivation of miR-34a and miR-34b/c by CpG methylation in colorectal, pancreatic, mammary, ovarian, urothelial, and renal cell carcinomas and soft tissue sarcomas. Virchows Arch 2011, 458(3):313-322.

124. Nalls D, Tang SN, Rodova M, Srivastava RK, Shankar S. Targeting epigenetic regulation of miR-34a for treatment of pancreatic cancer by inhibition of pancreatic cancer stem cells. PLoS One 2011, 6(8):e24099.

125. Walter BA, Valera VA, Pinto PA, Merino MJ. Comprehensive microRNA Profiling of Prostate Cancer. J Cancer 2013, 4(5):350-357.

126. Hagman Z, Haflidadottir BS, Ansari M, Persson M, Bjartell A, Edsjo A, Ceder Y. The tumour suppressor miR-34c targets MET in prostate cancer cells. Br J Cancer 2013.

127. Yu F, Jiao Y, Zhu Y, Wang Y, Zhu J, Cui X, Liu Y, He Y, Park EY, Zhang H *et al*. MicroRNA 34c gene down-regulation via DNA methylation promotes self-renewal and epithelial-mesenchymal transition in breast tumor-initiating cells. J Biol Chem 2012, 287(1):465-473.

128. Hagman Z, Larne O, Edsjo A, Bjartell A, Ehrnstrom RA, Ulmert D, Lilja H, Ceder Y. miR-34c is downregulated in prostate cancer and exerts tumor suppressive functions. Int J Cancer 2010, 127(12):2768-2776.

129. Fang L, Du WW, Yang W, Rutnam ZJ, Peng C, Li H, O'Malley YQ, Askeland RW, Sugg S, Liu M *et al*. MiR-93 enhances angiogenesis and metastasis by targeting LATS2. Cell Cycle 2012, 11(23):4352-4365.

130. Pineau P, Volinia S, McJunkin K, Marchio A, Battiston C, Terris B, Mazzaferro V, Lowe SW, Croce CM, Dejean A. miR-221 overexpression contributes to liver tumorigenesis. Proc Natl Acad Sci U S A 2010, 107(1):264-269.

131. Fang L, Deng Z, Shatseva T, Yang J, Peng C, Du WW, Yee AJ, Ang LC, He C, Shan SW *et al*. MicroRNA miR-93 promotes tumor growth and angiogenesis by targeting integrin-beta8. Oncogene 2011, 30(7):806-821.

132. Yu XF, Zou J, Bao ZJ, Dong J. miR-93 suppresses proliferation and colony formation of human colon cancer stem cells. World J Gastroenterol 2011, 17(42):4711-4717.

133. Fu X, Tian J, Zhang L, Chen Y, Hao Q. Involvement of microRNA-93, a new regulator of PTEN/Akt signaling pathway, in regulation of chemotherapeutic drug cisplatin chemosensitivity in ovarian cancer cells. FEBS Lett 2012, 586(9):1279-1286.

134. Song J, Bai Z, Han W, Zhang J, Meng H, Bi J, Ma X, Han S, Zhang Z. Identification of suitable reference genes for qPCR analysis of serum microRNA in gastric cancer patients. Dig Dis Sci 2012, 57(4):897-904.

135. Chen L, Jiang M, Yuan W, Tang H. Prognostic value of miR-93 overexpression in resectable gastric adenocarcinomas. Acta Gastroenterol Belg 2012, 75(1):22-27.

136. Kim YK, Yu J, Han TS, Park SY, Namkoong B, Kim DH, Hur K, Yoo MW, Lee HJ, Yang HK *et al*. Functional links between clustered microRNAs: suppression of cell-cycle inhibitors by microRNA clusters in gastric cancer. Nucleic Acids Res 2009, 37(5):1672-1681.

137. Chen X, Chen S, Xiu YL, Sun KX, Zong ZH, Zhao Y. RhoC is a major target of microRNA-93-5P in epithelial ovarian carcinoma tumorigenesis and progression. Mol Cancer 2015, 14(1):31.

138. Ohta K, Hoshino H, Wang J, Ono S, Iida Y, Hata K, Huang SK, Colquhoun S, Hoon DS. MicroRNA-93 activates c-Met/PI3K/Akt pathway activity in hepatocellular carcinoma by directly inhibiting PTEN and CDKN1A. Oncotarget 2014.

139. Tang Q, Zou Z, Zou C, Zhang Q, Huang R, Guan X, Li Q, Han Z, Wang D, Wei H *et al*. MicroRNA-93 suppress colorectal cancer development via Wnt/beta-catenin pathway downregulating. Tumour Biol 2014.

140. Du L, Zhao Z, Ma X, Hsiao TH, Chen Y, Young E, Suraokar M, Wistuba I, Minna JD, Pertsemlidis A. miR-93-directed downregulation of DAB2 defines a novel oncogenic pathway in lung cancer. Oncogene 2014, 33(34):4307-4315.

141. Deng ZQ, Qian J, Liu FQ, Lin J, Shao R, Yin JY, Tang Q, Zhang M, He L. Expression level of miR-93 in formalin-fixed paraffin-embedded tissues of breast cancer patients. Genet Test Mol Biomarkers 2014, 18(5):366-370.

142. Zhu W, He J, Chen D, Zhang B, Xu L, Ma H, Liu X, Zhang Y, Le H. Expression of miR-29c, miR-93, and miR-429 as potential biomarkers for detection of early stage non-small lung cancer. PLoS One 2014, 9(2):e87780.

143. Kim BH, Hong SW, Kim A, Choi SH, Yoon SO. Prognostic implications for high expression of oncogenic microRNAs in advanced gastric carcinoma. J Surg Oncol 2013, 107(5):505-510.

144. Koberle V, Kronenberger B, Pleli T, Trojan J, Imelmann E, Peveling-Oberhag J, Welker MW, Elhendawy M, Zeuzem S, Piiper A *et al*. Serum microRNA-1 and microRNA-122 are prognostic markers in patients with hepatocellular carcinoma. Eur J Cancer 2013.

145. Iino I, Kikuchi H, Miyazaki S, Hiramatsu Y, Ohta M, Kamiya K, Kusama Y, Baba S, Setou M, Konno H. Effect of miR-122 and its target gene cationic amino acid transporter 1 on colorectal liver metastasis. Cancer Sci 2013, 104(5):624-630.

146. Nana-Sinkam SP, Croce CM. Clinical applications for microRNAs in cancer. Clin Pharmacol Ther 2013, 93(1):98-104.

147. Wang B, Wang H, Yang Z. MiR-122 inhibits cell proliferation and tumorigenesis of breast cancer by targeting IGF1R. PLoS One 2012, 7(10):e47053.

148. Xu J, Zhu X, Wu L, Yang R, Yang Z, Wang Q, Wu F. MicroRNA-122 suppresses cell proliferation and induces cell apoptosis in hepatocellular carcinoma by directly targeting Wnt/beta-catenin pathway. Liver Int 2012, 32(5):752-760.

149. Lewis AP, Jopling CL. Regulation and biological function of the liver-specific miR-122. Biochem Soc Trans 2010, 38(6):1553-1557.

150. Fong MY, Zhou W, Liu L, Alontaga AY, Chandra M, Ashby J, Chow A, O'Connor ST, Li S, Chin AR *et al*. Breast-cancer-secreted miR-122 reprograms glucose metabolism in premetastatic niche to promote metastasis. Nat Cell Biol 2015, 17(2):183-194.

151. He J, Xie G, Tong J, Peng Y, Huang H, Li J, Wang N, Liang H. Overexpression of microRNA-122 re-sensitizes 5-FU-resistant colon cancer cells to 5-FU through the inhibition of PKM2 in vitro and in vivo. Cell Biochem Biophys 2014, 70(2):1343-1350.

152. Wang G, Zhao Y, Zheng Y. MiR-122/Wnt/beta-catenin regulatory circuitry sustains glioma progression. Tumour Biol 2014, 35(9):8565-8572.

153. Wang B, Hsu SH, Wang X, Kutay H, Bid HK, Yu J, Ganju RK, Jacob ST, Yuneva M, Ghoshal K. Reciprocal regulation of microRNA-122 and c-Myc in hepatocellular cancer: role of E2F1 and transcription factor dimerization partner 2. Hepatology 2014, 59(2):555-566.

154. Schultz NA, Dehlendorff C, Jensen BV, Bjerregaard JK, Nielsen KR, Bojesen SE, Calatayud D, Nielsen SE, Yilmaz M, Hollander NH *et al*. MicroRNA biomarkers in whole blood for detection of pancreatic cancer. JAMA 2014, 311(4):392-404.

155. Wang SC, Lin XL, Li J, Zhang TT, Wang HY, Shi JW, Yang S, Zhao WT, Xie RY, Wei F *et al*. MicroRNA-122 triggers mesenchymal-epithelial transition and suppresses hepatocellular carcinoma cell motility and invasion by targeting RhoA. PLoS One 2014, 9(7):e101330.

156. Liu AM, Xu Z, Shek FH, Wong KF, Lee NP, Poon RT, Chen J, Luk JM. miR-122 targets pyruvate kinase M2 and affects metabolism of hepatocellular carcinoma. PLoS One 2014, 9(1):e86872.

157. Lian JH, Wang WH, Wang JQ, Zhang YH, Li Y. MicroRNA-122 promotes proliferation, invasion and migration of renal cell carcinoma cells through the PI3K/Akt signaling pathway. Asian Pac J Cancer Prev 2013, 14(9):5017-5021.

158. Nassirpour R, Mehta PP, Yin MJ. miR-122 regulates tumorigenesis in hepatocellular carcinoma by targeting AKT3. PLoS One 2013, 8(11):e79655.

159. Boutz DR, Collins PJ, Suresh U, Lu M, Ramirez CM, Fernandez-Hernando C, Huang Y, Abreu Rde S, Le SY, Shapiro BA *et al*. Two-tiered approach identifies a network of cancer and liver disease-related genes regulated by miR-122. J Biol Chem 2011, 286(20):18066-18078.

160. Zhang Y, Jia Y, Zheng R, Guo Y, Wang Y, Guo H, Fei M, Sun S. Plasma microRNA-122 as a biomarker for viral-, alcohol-, and chemical-related hepatic diseases. Clin Chem 2010, 56(12):1830-1838.

161. O'Day E, Lal A. MicroRNAs and their target gene networks in breast cancer. Breast Cancer Res 2010, 12(2):201.

162. Laneve P, Di Marcotullio L, Gioia U, Fiori ME, Ferretti E, Gulino A, Bozzoni I, Caffarelli E. The interplay between microRNAs and the neurotrophin receptor tropomyosin-related kinase C controls proliferation of human neuroblastoma cells. Proc Natl Acad Sci U S A 2007, 104(19):7957-7962.

163. Ninio-Many L, Grossman H, Shomron N, Chuderland D, Shalgi R. microRNA-125a-3p reduces cells proliferation and migration by targeting Fyn. J Cell Sci 2013.

164. Hashiguchi Y, Nishida N, Mimori K, Sudo T, Tanaka F, Shibata K, Ishii H, Mochizuki H, Hase K, Doki Y *et al*. Down-regulation of miR-125a-3p in human gastric cancer and its clinicopathological significance. Int J Oncol 2012, 40(5):1477-1482.

165. Della Vittoria Scarpati G, Falcetta F, Carlomagno C, Ubezio P, Marchini S, De Stefano A, Singh VK, D'Incalci M, De Placido S, Pepe S. A specific miRNA signature correlates with complete pathological response to neoadjuvant chemoradiotherapy in locally advanced rectal cancer. Int J Radiat Oncol Biol Phys 2012, 83(4):1113-1119.

166. Ma Y, Zhang P, Yang J, Liu Z, Yang Z, Qin H. Candidate microRNA biomarkers in human colorectal cancer: systematic review profiling studies and experimental validation. Int J Cancer 2012, 130(9):2077-2087.

167. Jiang L, Huang Q, Zhang S, Zhang Q, Chang J, Qiu X, Wang E. Hsa-miR-125a-3p and hsa-miR-125a-5p are downregulated in non-small cell lung cancer and have inverse effects on invasion and migration of lung cancer cells. BMC Cancer 2010, 10:318.

168. Zhu WY, Luo B, An JY, He JY, Chen DD, Xu LY, Huang YY, Liu XG, Le HB, Zhang YK. Differential expression of miR-125a-5p and let-7e predicts the progression and prognosis of non-small cell lung cancer. Cancer Invest 2014, 32(8):394-401.

169. Kim SW, Ramasamy K, Bouamar H, Lin AP, Jiang D, Aguiar RC. MicroRNAs miR-125a and miR-125b constitutively activate the NF-kappaB pathway by targeting the tumor necrosis factor alpha-induced protein 3 (TNFAIP3, A20). Proc Natl Acad Sci U S A 2012, 109(20):7865-7870.

170. Jiang L, Huang Q, Chang J, Wang E, Qiu X. MicroRNA HSA-miR-125a-5p induces apoptosis by activating p53 in lung cancer cells. Exp Lung Res 2011, 37(7):387-398.

171. Wang G, Mao W, Zheng S, Ye J. Epidermal growth factor receptor-regulated miR-125a-5p--a metastatic inhibitor of lung cancer. FEBS J 2009, 276(19):5571-5578.

172. Li BL, Lu C, Lu W, Yang TT, Qu J, Hong X, Wan XP. miR-130b is an EMT-related microRNA that targets DICER1 for aggression in endometrial cancer. Med Oncol 2013, 30(1):484.

173. Zhou H, Wang K, Hu Z, Wen J. TGF-beta1 alters microRNA profile in human gastric cancer cells. Chin J Cancer Res 2013, 25(1):102-111.

174. Dong P, Karaayvaz M, Jia N, Kaneuchi M, Hamada J, Watari H, Sudo S, Ju J, Sakuragi N. Mutant p53 gain-of-function induces epithelial-mesenchymal transition through modulation of the miR-130b-ZEB1 axis. Oncogene 2013, 32(27):3286-3295.

175. Chen Z, Jin Y, Yu D, Wang A, Mahjabeen I, Wang C, Liu X, Zhou X. Down-regulation of the microRNA-99 family members in head and neck squamous cell carcinoma. Oral Oncol 2012, 48(8):686-691.

176. Yip L, Kelly L, Shuai Y, Armstrong MJ, Nikiforov YE, Carty SE, Nikiforova MN. MicroRNA signature distinguishes the degree of aggressiveness of papillary thyroid carcinoma. Ann Surg Oncol 2011, 18(7):2035-2041.

177. Lai KW, Koh KX, Loh M, Tada K, Subramaniam MM, Lim XY, Vaithilingam A, Salto-Tellez M, Iacopetta B, Ito Y *et al*. MicroRNA-130b regulates the tumour suppressor RUNX3 in gastric cancer. Eur J Cancer 2010, 46(8):1456-1463.

178. Yu T, Cao R, Li S, Fu M, Ren L, Chen W, Zhu H, Zhan Q, Shi R. MiR-130b plays an oncogenic role by repressing PTEN expression in esophageal squamous cell carcinoma cells. BMC Cancer 2015, 15(1):29.

179. Chen Q, Zhao X, Zhang H, Yuan H, Zhu M, Sun Q, Lai X, Wang Y, Huang J, Yan J *et al*. MiR-130b suppresses prostate cancer metastasis through down-regulation of MMP2. Mol Carcinog 2014.

180. Zhao Y, Miao G, Li Y, Isaji T, Gu J, Li J, Qi R. MicroRNA- 130b suppresses migration and invasion of colorectal cancer cells through downregulation of integrin beta1 [corrected]. PLoS One 2014, 9(2):e87938.

181. Zhao G, Zhang JG, Shi Y, Qin Q, Liu Y, Wang B, Tian K, Deng SC, Li X, Zhu S *et al*. MiR-130b is a prognostic marker and inhibits cell proliferation and invasion in pancreatic cancer through targeting STAT3. PLoS One 2013, 8(9):e73803.

182. Lin CW, Chang YL, Chang YC, Lin JC, Chen CC, Pan SH, Wu CT, Chen HY, Yang SC, Hong TM *et al*. MicroRNA-135b promotes lung cancer metastasis by regulating multiple targets in the Hippo pathway and LZTS1. Nat Commun 2013, 4:1877.

183. Lowery AJ, Miller N, Devaney A, McNeill RE, Davoren PA, Lemetre C, Benes V, Schmidt S, Blake J, Ball G *et al*. MicroRNA signatures predict oestrogen receptor, progesterone receptor and HER2/neu receptor status in breast cancer. Breast Cancer Res 2009, 11(3):R27.

184. Bandres E, Cubedo E, Agirre X, Malumbres R, Zarate R, Ramirez N, Abajo A, Navarro A, Moreno I, Monzo M *et al*. Identification by Real-time PCR of 13 mature microRNAs differentially expressed in colorectal cancer and non-tumoral tissues. Mol Cancer 2006, 5:29.

185. Wang G, Wang Y, Feng W, Wang X, Yang JY, Zhao Y, Wang Y, Liu Y. Transcription factor and microRNA regulation in androgen-dependent and -independent prostate cancer cells. BMC Genomics 2008, 9 Suppl 2:S22.

186. Matsuyama H, Suzuki HI, Nishimori H, Noguchi M, Yao T, Komatsu N, Mano H, Sugimoto K, Miyazono K. miR-135b mediates NPM-ALK-driven oncogenicity and renders IL-17-producing immunophenotype to anaplastic large cell lymphoma. Blood 2011, 118(26):6881-6892.

187. Wang Z, Zhang H, He L, Dong W, Li J, Shan Z, Teng W. Association between the expression of four upregulated miRNAs and extrathyroidal invasion in papillary thyroid carcinoma. Onco Targets Ther 2013, 6:281-287.

188. Arigoni M, Barutello G, Riccardo F, Ercole E, Cantarella D, Orso F, Conti L, Lanzardo S, Taverna D, Merighi I *et al*. miR-135b Coordinates Progression of ErbB2-Driven Mammary Carcinomas through Suppression of MID1 and MTCH2. Am J Pathol 2013, 182(6):2058-2070.

189. Xu XM, Qian JC, Deng ZL, Cai Z, Tang T, Wang P, Zhang KH, Cai JP. Expression of miR-21, miR-31, miR-96 and miR-135b is correlated with the clinical parameters of colorectal cancer. Oncol Lett 2012, 4(2):339-345.

190. Faltejskova P, Bocanek O, Sachlova M, Svoboda M, Kiss I, Vyzula R, Slaby O. Circulating miR-17-3p, miR-29a, miR-92a and miR-135b in serum: Evidence against their usage as biomarkers in colorectal cancer. Cancer Biomark 2012, 12(4):199-204.

191. Munding JB, Adai AT, Maghnouj A, Urbanik A, Zollner H, Liffers ST, Chromik AM, Uhl W, Szafranska-Schwarzbach AE, Tannapfel A *et al*. Global microRNA expression profiling of microdissected tissues identifies miR-135b as a novel biomarker for pancreatic ductal adenocarcinoma. Int J Cancer 2012, 131(2):E86-95.

192. Valeri N, Braconi C, Gasparini P, Murgia C, Lampis A, Paulus-Hock V, Hart JR, Ueno L, Grivennikov SI, Lovat F *et al*. MicroRNA-135b promotes cancer progression by acting as a downstream effector of oncogenic pathways in colon cancer. Cancer Cell 2014, 25(4):469-483.

193. Haapa-Paananen S, Chen P, Hellstrom K, Kohonen P, Hautaniemi S, Kallioniemi O, Perala M. Functional profiling of precursor MicroRNAs identifies MicroRNAs essential for glioma proliferation. PLoS One 2013, 8(4):e60930.

194. Yang Y, Wu J, Guan H, Cai J, Fang L, Li J, Li M. MiR-136 promotes apoptosis of glioma cells by targeting AEG-1 and Bcl-2. FEBS Lett 2012, 586(20):3608-3612.

195. Zhang Z, Zhang Y, Sun XX, Ma X, Chen ZN. microRNA-146a inhibits cancer metastasis by downregulating VEGF through dual pathways in hepatocellular carcinoma. Mol Cancer 2015, 14(1):5.

196. Hong TH, Park IY. MicroRNA expression profiling of diagnostic needle aspirates from surgical pancreatic cancer specimens. Ann Surg Treat Res 2014, 87(6):290-297.

197. Sun Q, Zhao X, Liu X, Wang Y, Huang J, Jiang B, Chen Q, Yu J. miR-146a functions as a tumor suppressor in prostate cancer by targeting Rac1. Prostate 2014, 74(16):1613-1621.

198. Kumaraswamy E, Wendt KL, Augustine LA, Stecklein SR, Sibala EC, Li D, Gunewardena S, Jensen RA. BRCA1 regulation of epidermal growth factor receptor (EGFR) expression in human breast cancer cells involves microRNA-146a and is critical for its tumor suppressor function. Oncogene 2014.

199. Cornett AL, Lutz CS. Regulation of COX-2 expression by miR-146a in lung cancer cells. RNA 2014, 20(9):1419-1430.

200. Ali S, Ahmad A, Aboukameel A, Ahmed A, Bao B, Banerjee S, Philip PA, Sarkar FH. Deregulation of miR-146a expression in a mouse model of pancreatic cancer affecting EGFR signaling. Cancer Lett 2014, 351(1):134-142.

201. Li H, Xie S, Liu M, Chen Z, Liu X, Wang L, Li D, Zhou Y. The clinical significance of downregulation of mir-124-3p, mir-146a-5p, mir-155-5p and mir-335-5p in gastric cancer tumorigenesis. Int J Oncol 2014, 45(1):197-208.

202. Yao Q, Cao Z, Tu C, Zhao Y, Liu H, Zhang S. MicroRNA-146a acts as a metastasis suppressor in gastric cancer by targeting WASF2. Cancer Lett 2013, 335(1):219-224.

203. Yu J, Li A, Hong SM, Hruban RH, Goggins M. MicroRNA alterations of pancreatic intraepithelial neoplasias. Clin Cancer Res 2012, 18(4):981-992.

204. Xiao B, Zhu ED, Li N, Lu DS, Li W, Li BS, Zhao YL, Mao XH, Guo G, Yu PW *et al*. Increased miR-146a in gastric cancer directly targets SMAD4 and is involved in modulating cell proliferation and apoptosis. Oncol Rep 2012, 27(2):559-566.

205. Li Y, VandenBoom TG, 2nd, Wang Z, Kong D, Ali S, Philip PA, Sarkar FH. Up-regulation of miR-146a contributes to the inhibition of invasion of pancreatic cancer cells. Cancer Res 2010, 70(8 Suppl):5703.

206. Hanoun N, Delpu Y, Suriawinata AA, Bournet B, Bureau C, Selves J, Tsongalis GJ, Dufresne M, Buscail L, Cordelier P *et al*. The silencing of microRNA 148a production by DNA hypermethylation is an early event in pancreatic carcinogenesis. Clin Chem 2010, 56(7):1107-1118.

207. Sandhu R, Rivenbark AG, Mackler RM, Livasy CA, Coleman WB. Dysregulation of microRNA expression drives aberrant DNA hypermethylation in basal-like breast cancer. Int J Oncol 2013.

208. Sakamoto N, Naito Y, Oue N, Sentani K, Uraoka N, Oo HZ, Yanagihara K, Aoyagi K, Sasaki H, Yasui W. MiR-148a is down-regulated in gastric cancer, targets MMP7 and indicates tumor invasiveness and poor prognosis. Cancer Sci 2013.

209. Kjersem JB, Ikdahl T, Lingjaerde OC, Guren T, Tveit KM, Kure EH. Plasma microRNAs predicting clinical outcome in metastatic colorectal cancer patients receiving first-line oxaliplatin-based treatment. Mol Oncol 2013.

210. Zhang JP, Zeng C, Xu L, Gong J, Fang JH, Zhuang SM. MicroRNA-148a suppresses the epithelial-mesenchymal transition and metastasis of hepatoma cells by targeting Met/Snail signaling. Oncogene 2013.

211. Zhang R, Li M, Zang W, Chen X, Wang Y, Li P, Du Y, Zhao G, Li L. MiR-148a regulates the growth and apoptosis in pancreatic cancer by targeting CCKBR and Bcl-2. Tumour Biol 2013.

212. Kim SY, Jeon TY, Choi CI, Kim DH, Kim DH, Kim GH, Ryu DY, Lee BE, Kim HH. Validation of circulating miRNA biomarkers for predicting lymph node metastasis in gastric cancer. J Mol Diagn 2013, 15(5):661-669.

213. Li J, Song Y, Wang Y, Luo J, Yu W. MicroRNA-148a suppresses epithelial-to-mesenchymal transition by targeting ROCK1 in non-small cell lung cancer cells. Mol Cell Biochem 2013, 380(1-2):277-282.

214. Liffers ST, Munding JB, Vogt M, Kuhlmann JD, Verdoodt B, Nambiar S, Maghnouj A, Mirmohammadsadegh A, Hahn SA, Tannapfel A. MicroRNA-148a is down-regulated in human pancreatic ductal adenocarcinomas and regulates cell survival by targeting CDC25B. Lab Invest 2011, 91(10):1472-1479.

215. LaConti JJ, Shivapurkar N, Preet A, Deslattes Mays A, Peran I, Kim SE, Marshall JL, Riegel AT, Wellstein A. Tissue and serum microRNAs in the Kras(G12D) transgenic animal model and in patients with pancreatic cancer. PLoS One 2011, 6(6):e20687.

216. Guo SL, Peng Z, Yang X, Fan KJ, Ye H, Li ZH, Wang Y, Xu XL, Li J, Wang YL *et al*. miR-148a promoted cell proliferation by targeting p27 in gastric cancer cells. Int J Biol Sci 2011, 7(5):567-574.

217. Yu J, Li Q, Xu Q, Liu L, Jiang B. MiR-148a inhibits angiogenesis by targeting ERBB3. J Biomed Res 2011, 25(3):170-177.

218. Murata T, Takayama K, Katayama S, Urano T, Horie-Inoue K, Ikeda K, Takahashi S, Kawazu C, Hasegawa A, Ouchi Y *et al*. miR-148a is an androgen-responsive microRNA that promotes LNCaP prostate cell growth by repressing its target CAND1 expression. Prostate Cancer Prostatic Dis 2010, 13(4):356-361.

219. Szafranska AE, Davison TS, John J, Cannon T, Sipos B, Maghnouj A, Labourier E, Hahn SA. MicroRNA expression alterations are linked to tumorigenesis and non-neoplastic processes in pancreatic ductal adenocarcinoma. Oncogene 2007, 26(30):4442-4452.

220. Greither T, Grochola LF, Udelnow A, Lautenschlager C, Wurl P, Taubert H. Elevated expression of microRNAs 155, 203, 210 and 222 in pancreatic tumors is associated with poorer survival. Int J Cancer 2010, 126(1):73-80.

221. Jamali Z, Asl Aminabadi N, Attaran R, Pournagiazar F, Ghertasi Oskouei S, Ahmadpour F. MicroRNAs as prognostic molecular signatures in human head and neck squamous cell carcinoma: A systematic review and meta-analysis. Oral Oncol 2015, 51(4):321-331.

222. Chen Z, Ma T, Huang C, Hu T, Li J. The pivotal role of microRNA-155 in the control of cancer. J Cell Physiol 2014, 229(5):545-550.

223. Vigorito E, Kohlhaas S, Lu D, Leyland R. miR-155: an ancient regulator of the immune system. Immunol Rev 2013, 253(1):146-157.

224. Yang M, Shen H, Qiu C, Ni Y, Wang L, Dong W, Liao Y, Du J. High expression of miR-21 and miR-155 predicts recurrence and unfavourable survival in non-small cell lung cancer. Eur J Cancer 2013, 49(3):604-615.

225. Mattiske S, Suetani RJ, Neilsen PM, Callen DF. The oncogenic role of miR-155 in breast cancer. Cancer Epidemiol Biomarkers Prev 2012, 21(8):1236-1243.

226. Kohanbash G, Okada H. MicroRNAs and STAT interplay. Semin Cancer Biol 2012, 22(1):70-75.

227. Cai ZK, Chen Q, Chen YB, Gu M, Zheng DC, Zhou J, Wang Z. microRNA-155 promotes the proliferation of prostate cancer cells by targeting annexin 7. Mol Med Rep 2015, 11(1):533-538.

228. Lao G, Liu P, Wu Q, Zhang W, Liu Y, Yang L, Ma C. Mir-155 promotes cervical cancer cell proliferation through suppression of its target gene LKB1. Tumour Biol 2014, 35(12):11933-11938.

229. Liu Q, Chen J, Wang J, Amos C, Killary AM, Sen S, Wei C, Frazier ML. Putative tumor suppressor gene SEL1L was downregulated by aberrantly upregulated hsa-mir-155 in human pancreatic ductal adenocarcinoma. Mol Carcinog 2014, 53(9):711-721.

230. Sun S, Sun P, Wang C, Sun T. Downregulation of microRNA-155 accelerates cell growth and invasion by targeting c-myc in human gastric carcinoma cells. Oncol Rep 2014, 32(3):951-956.

231. Dinami R, Ercolani C, Petti E, Piazza S, Ciani Y, Sestito R, Sacconi A, Biagioni F, le Sage C, Agami R *et al*. miR-155 drives telomere fragility in human breast cancer by targeting TRF1. Cancer Res 2014, 74(15):4145-4156.

232. Zhang J, Cheng C, Yuan X, He JT, Pan QH, Sun FY. microRNA-155 acts as an oncogene by targeting the tumor protein 53-induced nuclear protein 1 in esophageal squamous cell carcinoma. Int J Clin Exp Pathol 2014, 7(2):602-610.

233. Johansson J, Berg T, Kurzejamska E, Pang MF, Tabor V, Jansson M, Roswall P, Pietras K, Sund M, Religa P *et al*. MiR-155-mediated loss of C/EBPbeta shifts the TGF-beta response from growth inhibition to epithelial-mesenchymal transition, invasion and metastasis in breast cancer. Oncogene 2013, 32(50):5614-5624.

234. Liu WJ, Zhao YP, Zhang TP, Zhou L, Cui QC, Zhou WX, You L, Chen G, Shu H. MLH1 as a direct target of MiR-155 and a potential predictor of favorable prognosis in pancreatic cancer. J Gastrointest Surg 2013, 17(8):1399-1405.

235. Cai J, Wu J, Zhang H, Fang L, Huang Y, Yang Y, Zhu X, Li R, Li M. miR-186 downregulation correlates with poor survival in lung adenocarcinoma, where it interferes with cell-cycle regulation. Cancer Res 2013, 73(2):756-766.

236. Zhang Y, Li M, Wang H, Fisher WE, Lin PH, Yao Q, Chen C. Profiling of 95 microRNAs in pancreatic cancer cell lines and surgical specimens by real-time PCR analysis. World J Surg 2009, 33(4):698-709.

237. Li H, Yin C, Zhang B, Sun Y, Shi L, Liu N, Liang S, Lu S, Liu Y, Zhang J *et al*. PTTG1 promotes migration and invasion of human non-small cell lung cancer cells and is modulated by miR-186. Carcinogenesis 2013, 34(9):2145-2155.

238. Cui G, Cui M, Li Y, Liang Y, Li W, Guo H, Zhao S. MiR-186 targets ROCK1 to suppress the growth and metastasis of NSCLC cells. Tumour Biol 2014, 35(9):8933-8937.

239. Zhang J, Zhang T, Ti X, Shi J, Wu C, Ren X, Yin H. Curcumin promotes apoptosis in A549/DDP multidrug-resistant human lung adenocarcinoma cells through an miRNA signaling pathway. Biochem Biophys Res Commun 2010, 399(1):1-6.

240. Zhao BS, Liu SG, Wang TY, Ji YH, Qi B, Tao YP, Li HC, Wu XN. Screening of microRNA in patients with esophageal cancer at same tumor node metastasis stage with different prognoses. Asian Pac J Cancer Prev 2013, 14(1):139-143.

241. Erdmann K, Kaulke K, Thomae C, Huebner D, Sergon M, Froehner M, Wirth MP, Fuessel S. Elevated expression of prostate cancer-associated genes is linked to down-regulation of microRNAs. BMC Cancer 2014, 14:82.

242. Mulrane L, Madden SF, Brennan DJ, Gremel G, McGee SF, McNally S, Martin F, Crown JP, Jirstrom K, Higgins DG *et al*. miR-187 is an independent prognostic factor in breast cancer and confers increased invasive potential in vitro. Clin Cancer Res 2012, 18(24):6702-6713.

243. Fuse M, Kojima S, Enokida H, Chiyomaru T, Yoshino H, Nohata N, Kinoshita T, Sakamoto S, Naya Y, Nakagawa M *et al*. Tumor suppressive microRNAs (miR-222 and miR-31) regulate molecular pathways based on microRNA expression signature in prostate cancer. J Hum Genet 2012, 57(11):691-699.

244. Wu X, Liu T, Fang O, Leach LJ, Hu X, Luo Z. miR-194 suppresses metastasis of non-small cell lung cancer through regulating expression of BMP1 and p27. Oncogene 2013.

245. Chiang Y, Song Y, Wang Z, Liu Z, Gao P, Liang J, Zhu J, Xing C, Xu H. microRNA-192, -194 and -215 are frequently downregulated in colorectal cancer. Exp Ther Med 2012, 3(3):560-566.

246. Senanayake U, Das S, Vesely P, Alzoughbi W, Frohlich LF, Chowdhury P, Leuschner I, Hoefler G, Guertl B. miR-192, miR-194, miR-215, miR-200c and miR-141 are downregulated and their common target ACVR2B is strongly expressed in renal childhood neoplasms. Carcinogenesis 2012, 33(5):1014-1021.

247. Dong P, Kaneuchi M, Watari H, Hamada J, Sudo S, Ju J, Sakuragi N. MicroRNA-194 inhibits epithelial to mesenchymal transition of endometrial cancer cells by targeting oncogene BMI-1. Mol Cancer 2011, 10:99.

248. Song Y, Zhao F, Wang Z, Liu Z, Chiang Y, Xu Y, Gao P, Xu H. Inverse association between miR-194 expression and tumor invasion in gastric cancer. Ann Surg Oncol 2012, 19 Suppl 3:S509-517.

249. Namlos HM, Meza-Zepeda LA, Baroy T, Ostensen IH, Kresse SH, Kuijjer ML, Serra M, Burger H, Cleton-Jansen AM, Myklebost O. Modulation of the osteosarcoma expression phenotype by microRNAs. PLoS One 2012, 7(10):e48086.

250. Abe W, Nasu K, Nakada C, Kawano Y, Moriyama M, Narahara H. miR-196b targets c-myc and Bcl-2 expression, inhibits proliferation and induces apoptosis in endometriotic stromal cells. Hum Reprod 2013, 28(3):750-761.

251. Shen J, Wang S, Zhang YJ, Kappil MA, Chen Wu H, Kibriya MG, Wang Q, Jasmine F, Ahsan H, Lee PH *et al*. Genome-wide aberrant DNA methylation of microRNA host genes in hepatocellular carcinoma. Epigenetics 2012, 7(11):1230-1237.

252. Lu YC, Chen YJ, Wang HM, Tsai CY, Chen WH, Huang YC, Fan KH, Tsai CN, Huang SF, Kang CJ *et al*. Oncogenic function and early detection potential of miRNA-10b in oral cancer as identified by microRNA profiling. Cancer Prev Res (Phila) 2012, 5(4):665-674.

253. de Oliveira JC, Scrideli CA, Brassesco MS, Morales AG, Pezuk JA, Queiroz Rde P, Yunes JA, Brandalise SR, Tone LG. Differential miRNA expression in childhood acute lymphoblastic leukemia and association with clinical and biological features. Leuk Res 2012, 36(3):293-298.

254. Lakomy R, Sana J, Hankeova S, Fadrus P, Kren L, Lzicarova E, Svoboda M, Dolezelova H, Smrcka M, Vyzula R *et al*. MiR-195, miR-196b, miR-181c, miR-21 expression levels and O-6-methylguanine-DNA methyltransferase methylation status are associated with clinical outcome in glioblastoma patients. Cancer Sci 2011, 102(12):2186-2190.

255. Tsai KW, Hu LY, Wu CW, Li SC, Lai CH, Kao HW, Fang WL, Lin WC. Epigenetic regulation of miR-196b expression in gastric cancer. Genes Chromosomes Cancer 2010, 49(11):969-980.

256. Coskun E, von der Heide EK, Schlee C, Kuhnl A, Gokbuget N, Hoelzer D, Hofmann WK, Thiel E, Baldus CD. The role of microRNA-196a and microRNA-196b as ERG regulators in acute myeloid leukemia and acute T-lymphoblastic leukemia. Leuk Res 2011, 35(2):208-213.

257. Wang Y, Li Z, He C, Wang D, Yuan X, Chen J, Jin J. MicroRNAs expression signatures are associated with lineage and survival in acute leukemias. Blood Cells Mol Dis 2010, 44(3):191-197.

258. Liu SG, Qin XG, Zhao BS, Qi B, Yao WJ, Wang TY, Li HC, Wu XN. Differential expression of miRNAs in esophageal cancer tissue. Oncol Lett 2013, 5(5):1639-1642.

259. Plummer PN, Freeman R, Taft RJ, Vider J, Sax M, Umer BA, Gao D, Johns C, Mattick JS, Wilton SD *et al*. MicroRNAs regulate tumor angiogenesis modulated by endothelial progenitor cells. Cancer Res 2013, 73(1):341-352.

260. Li Z, Huang H, Chen P, He M, Li Y, Arnovitz S, Jiang X, He C, Hyjek E, Zhang J *et al*. miR-196b directly targets both HOXA9/MEIS1 oncogenes and FAS tumour suppressor in MLL-rearranged leukaemia. Nat Commun 2012, 3:688.

261. Li Y, Zhang M, Chen H, Dong Z, Ganapathy V, Thangaraju M, Huang S. Ratio of miR-196s to HOXC8 messenger RNA correlates with breast cancer cell migration and metastasis. Cancer Res 2010, 70(20):7894-7904.

262. Wang YX, Zhang XY, Zhang BF, Yang CQ, Chen XM, Gao HJ. Initial study of microRNA expression profiles of colonic cancer without lymph node metastasis. J Dig Dis 2010, 11(1):50-54.

263. Szafranska AE, Doleshal M, Edmunds HS, Gordon S, Luttges J, Munding JB, Barth RJ, Jr., Gutmann EJ, Suriawinata AA, Marc Pipas J *et al*. Analysis of microRNAs in pancreatic fine-needle aspirates can classify benign and malignant tissues. Clin Chem 2008, 54(10):1716-1724.

264. Li X, Shi Y, Yin Z, Xue X, Zhou B. An eight-miRNA signature as a potential biomarker for predicting survival in lung adenocarcinoma. J Transl Med 2014, 12:159.

265. Boisen MK, Dehlendorff C, Linnemann D, Nielsen BS, Larsen JS, Osterlind K, Nielsen SE, Tarpgaard LS, Qvortrup C, Pfeiffer P *et al*. Tissue microRNAs as predictors of outcome in patients with metastatic colorectal cancer treated with first line Capecitabine and Oxaliplatin with or without Bevacizumab. PLoS One 2014, 9(10):e109430.

266. How C, Hui AB, Alajez NM, Shi W, Boutros PC, Clarke BA, Yan R, Pintilie M, Fyles A, Hedley DW *et al*. MicroRNA-196b regulates the homeobox B7-vascular endothelial growth factor axis in cervical cancer. PLoS One 2013, 8(7):e67846.

267. Luthra R, Singh RR, Luthra MG, Li YX, Hannah C, Romans AM, Barkoh BA, Chen SS, Ensor J, Maru DM *et al*. MicroRNA-196a targets annexin A1: a microRNA-mediated mechanism of annexin A1 downregulation in cancers. Oncogene 2008, 27(52):6667-6678.

268. Zhao JJ, Yang J, Lin J, Yao N, Zhu Y, Zheng J, Xu J, Cheng JQ, Lin JY, Ma X. Identification of miRNAs associated with tumorigenesis of retinoblastoma by miRNA microarray analysis. Childs Nerv Syst 2009, 25(1):13-20.

269. Sonkoly E, Stahle M, Pivarcsi A. MicroRNAs and immunity: novel players in the regulation of normal immune function and inflammation. Semin Cancer Biol 2008, 18(2):131-140.

270. Wei T, Xu N, Meisgen F, Stahle M, Sonkoly E, Pivarcsi A. Interleukin-8 is regulated by miR-203 at the posttranscriptional level in primary human keratinocytes. Eur J Dermatol 2013.

271. Zhang K, Dai L, Zhang B, Xu X, Shi J, Fu L, Chen X, Li J, Bai Y. miR-203 is a direct transcriptional target of E2F1 and causes G1 arrest in esophageal cancer cells. J Cell Physiol 2015, 230(4):903-910.

272. Xiang J, Bian C, Wang H, Huang S, Wu D. MiR-203 down-regulates Rap1A and suppresses cell proliferation, adhesion and invasion in prostate cancer. J Exp Clin Cancer Res 2015, 34(1):8.

273. Taipaleenmaki H, Browne G, Akech J, Zustin J, van Wijnen AJ, Stein JL, Hesse E, Stein GS, Lian JB. Targeting of Runx2 by miRNA-135 and miRNA-203 Impairs Progression of Breast Cancer and Metastatic Bone Disease. Cancer Res 2015.

274. Mao L, Zhang Y, Mo W, Yu Y, Lu H. BANF1 Is Downregulated by IRF1-Regulated MicroRNA-203 in Cervical Cancer. PLoS One 2015, 10(2):e0117035.

275. Li Z, Du L, Dong Z, Yang Y, Zhang X, Wang L, Li J, Zheng G, Qu A, Wang C. MiR-203 Suppresses ZNF217 Upregulation in Colorectal Cancer and Its Oncogenicity. PLoS One 2015, 10(1):e0116170.

276. Zhou M, Chen J, Zhou L, Chen W, Ding G, Cao L. Pancreatic cancer derived exosomes regulate the expression of TLR4 in dendritic cells via miR-203. Cell Immunol 2014, 292(1-2):65-69.

277. Stoyianni A, Pentheroudakis G, Benjamin H, Cervantes A, Ashkenazi K, Lazaridis G, Pavlidis N, Spector Y. Insights into the epithelial mesenchymal transition phenotype in cancer of unknown primary from a global microRNA profiling study. Clin Transl Oncol 2014, 16(8):725-731.

278. Hailer A, Grunewald TG, Orth M, Reiss C, Kneitz B, Spahn M, Butt E. Loss of tumor suppressor mir-203 mediates overexpression of LIM and SH3 Protein 1 (LASP1) in high-risk prostate cancer thereby increasing cell proliferation and migration. Oncotarget 2014, 5(12):4144-4153.

279. Siu MK, Abou-Kheir W, Yin JJ, Chang YS, Barrett B, Suau F, Casey O, Chen WY, Fang L, Hynes P *et al*. Loss of EGFR signaling regulated miR-203 promotes prostate cancer bone metastasis and tyrosine kinase inhibitors resistance. Oncotarget 2014, 5(11):3770-3784.

280. Yu X, Jiang X, Li H, Guo L, Jiang W, Lu SH. miR-203 inhibits the proliferation and self-renewal of esophageal cancer stem-like cells by suppressing stem renewal factor Bmi-1. Stem Cells Dev 2014, 23(6):576-585.

281. Miao L, Xiong X, Lin Y, Cheng Y, Lu J, Zhang J, Cheng N. miR-203 inhibits tumor cell migration and invasion via caveolin-1 in pancreatic cancer cells. Oncol Lett 2014, 7(3):658-662.

282. Zhang F, Yang Z, Cao M, Xu Y, Li J, Chen X, Gao Z, Xin J, Zhou S, Zhou Z *et al*. MiR-203 suppresses tumor growth and invasion and down-regulates MiR-21 expression through repressing Ran in esophageal cancer. Cancer Lett 2014, 342(1):121-129.

283. Wang N, Liang H, Zhou Y, Wang C, Zhang S, Pan Y, Wang Y, Yan X, Zhang J, Zhang CY *et al*. miR-203 suppresses the proliferation and migration and promotes the apoptosis of lung cancer cells by targeting SRC. PLoS One 2014, 9(8):e105570.

284. Ding X, Park SI, McCauley LK, Wang CY. Signaling between transforming growth factor beta (TGF-beta) and transcription factor SNAI2 represses expression of microRNA miR-203 to promote epithelial-mesenchymal transition and tumor metastasis. J Biol Chem 2013, 288(15):10241-10253.

285. Jin J, Deng J, Wang F, Xia X, Qiu T, Lu W, Li X, Zhang H, Gu X, Liu Y *et al*. The expression and function of microRNA-203 in lung cancer. Tumour Biol 2013, 34(1):349-357.

286. Zhu X, Er K, Mao C, Yan Q, Xu H, Zhang Y, Zhu J, Cui F, Zhao W, Shi H. miR-203 suppresses tumor growth and angiogenesis by targeting VEGFA in cervical cancer. Cell Physiol Biochem 2013, 32(1):64-73.

287. Wang S, Zhao X, Wang J, Wen Y, Zhang L, Wang D, Chen H, Chen Q, Xiang W. Upregulation of microRNA-203 is associated with advanced tumor progression and poor prognosis in epithelial ovarian cancer. Med Oncol 2013, 30(3):681.

288. Wang C, Wang X, Liang H, Wang T, Yan X, Cao M, Wang N, Zhang S, Zen K, Zhang C *et al*. miR-203 inhibits cell proliferation and migration of lung cancer cells by targeting PKCalpha. PLoS One 2013, 8(9):e73985.

289. Takeshita N, Mori M, Kano M, Hoshino I, Akutsu Y, Hanari N, Yoneyama Y, Ikeda N, Isozaki Y, Maruyama T *et al*. miR-203 inhibits the migration and invasion of esophageal squamous cell carcinoma by regulating LASP1. Int J Oncol 2012, 41(5):1653-1661.

290. Wang C, Zheng X, Shen C, Shi Y. MicroRNA-203 suppresses cell proliferation and migration by targeting BIRC5 and LASP1 in human triple-negative breast cancer cells. J Exp Clin Cancer Res 2012, 31:58.

291. Viticchie G, Lena AM, Latina A, Formosa A, Gregersen LH, Lund AH, Bernardini S, Mauriello A, Miano R, Spagnoli LG *et al*. MiR-203 controls proliferation, migration and invasive potential of prostate cancer cell lines. Cell Cycle 2011, 10(7):1121-1131.

292. Bo J, Yang G, Huo K, Jiang H, Zhang L, Liu D, Huang Y. microRNA-203 suppresses bladder cancer development by repressing bcl-w expression. FEBS J 2011, 278(5):786-792.

293. Ikenaga N, Ohuchida K, Mizumoto K, Yu J, Kayashima T, Sakai H, Fujita H, Nakata K, Tanaka M. MicroRNA-203 expression as a new prognostic marker of pancreatic adenocarcinoma. Ann Surg Oncol 2010, 17(12):3120-3128.

294. Sempere LF, Christensen M, Silahtaroglu A, Bak M, Heath CV, Schwartz G, Wells W, Kauppinen S, Cole CN. Altered MicroRNA expression confined to specific epithelial cell subpopulations in breast cancer. Cancer Res 2007, 67(24):11612-11620.

295. Feber A, Xi L, Luketich JD, Pennathur A, Landreneau RJ, Wu M, Swanson SJ, Godfrey TE, Litle VR. MicroRNA expression profiles of esophageal cancer. J Thorac Cardiovasc Surg 2008, 135(2):255-260; discussion 260.

296. Iorio MV, Visone R, Di Leva G, Donati V, Petrocca F, Casalini P, Taccioli C, Volinia S, Liu CG, Alder H *et al*. MicroRNA signatures in human ovarian cancer. Cancer Res 2007, 67(18):8699-8707.

297. Gottardo F, Liu CG, Ferracin M, Calin GA, Fassan M, Bassi P, Sevignani C, Byrne D, Negrini M, Pagano F *et al*. Micro-RNA profiling in kidney and bladder cancers. Urol Oncol 2007, 25(5):387-392.

298. Gregory PA, Bracken CP, Bert AG, Goodall GJ. MicroRNAs as regulators of epithelial-mesenchymal transition. Cell Cycle 2008, 7(20):3112-3118.

299. Verdoodt B, Neid M, Vogt M, Kuhn V, Liffers ST, Palisaar RJ, Noldus J, Tannapfel A, Mirmohammadsadegh A. MicroRNA-205, a novel regulator of the anti-apoptotic protein Bcl2, is downregulated in prostate cancer. Int J Oncol 2013, 43(1):307-314.

300. Qin AY, Zhang XW, Liu L, Yu JP, Li H, Wang SZ, Ren XB, Cao S. MiR-205 in cancer: an angel or a devil? Eur J Cell Biol 2013, 92(2):54-60.

301. Wang J, Raimondo M, Guha S, Chen J, Diao L, Dong X, Wallace MB, Killary AM, Frazier ML, Woodward TA *et al*. Circulating microRNAs in Pancreatic Juice as Candidate Biomarkers of Pancreatic Cancer. J Cancer 2014, 5(8):696-705.

302. Zhang G, Hou X, Li Y, Zhao M. MiR-205 inhibits cell apoptosis by targeting phosphatase and tensin homolog deleted on chromosome ten in endometrial cancer Ishikawa cells. BMC Cancer 2014, 14:440.

303. Larzabal L, de Aberasturi AL, Redrado M, Rueda P, Rodriguez MJ, Bodegas ME, Montuenga LM, Calvo A. TMPRSS4 regulates levels of integrin alpha5 in NSCLC through miR-205 activity to promote metastasis. Br J Cancer 2014, 110(3):764-774.

304. Markou A, Yousef GM, Stathopoulos E, Georgoulias V, Lianidou E. Prognostic significance of metastasis-related microRNAs in early breast cancer patients with a long follow-up. Clin Chem 2014, 60(1):197-205.

305. Yin WZ, Li F, Zhang L, Ren XP, Zhang N, Wen JF. Down-regulation of microRNA-205 promotes gastric cancer cell proliferation. Eur Rev Med Pharmacol Sci 2014, 18(7):1027-1032.

306. Lei L, Huang Y, Gong W. miR-205 promotes the growth, metastasis and chemoresistance of NSCLC cells by targeting PTEN. Oncol Rep 2013, 30(6):2897-2902.

307. Singh S, Chitkara D, Kumar V, Behrman SW, Mahato RI. miRNA profiling in pancreatic cancer and restoration of chemosensitivity. Cancer Lett 2013, 334(2):211-220.

308. Su N, Qiu H, Chen Y, Yang T, Yan Q, Wan X. miR-205 promotes tumor proliferation and invasion through targeting ESRRG in endometrial carcinoma. Oncol Rep 2013, 29(6):2297-2302.

309. Solomides CC, Evans BJ, Navenot JM, Vadigepalli R, Peiper SC, Wang ZX. MicroRNA profiling in lung cancer reveals new molecular markers for diagnosis. Acta Cytol 2012, 56(6):645-654.

310. Xie H, Zhao Y, Caramuta S, Larsson C, Lui WO. miR-205 expression promotes cell proliferation and migration of human cervical cancer cells. PLoS One 2012, 7(10):e46990.

311. Majid S, Saini S, Dar AA, Hirata H, Shahryari V, Tanaka Y, Yamamura S, Ueno K, Zaman MS, Singh K *et al*. MicroRNA-205 inhibits Src-mediated oncogenic pathways in renal cancer. Cancer Res 2011, 71(7):2611-2621.

312. Iorio MV, Casalini P, Piovan C, Di Leva G, Merlo A, Triulzi T, Menard S, Croce CM, Tagliabue E. microRNA-205 regulates HER3 in human breast cancer. Cancer Res 2009, 69(6):2195-2200.

313. Chan YC, Banerjee J, Choi SY, Sen CK. miR-210: the master hypoxamir. Microcirculation 2012, 19(3):215-223.

314. Miko E, Czimmerer Z, Csanky E, Boros G, Buslig J, Dezso B, Scholtz B. Differentially expressed microRNAs in small cell lung cancer. Exp Lung Res 2009, 35(8):646-664.

315. Tsuchiya S, Fujiwara T, Sato F, Shimada Y, Tanaka E, Sakai Y, Shimizu K, Tsujimoto G. MicroRNA-210 regulates cancer cell proliferation through targeting fibroblast growth factor receptor-like 1 (FGFRL1). J Biol Chem 2011, 286(1):420-428.

316. Rothe F, Ignatiadis M, Chaboteaux C, Haibe-Kains B, Kheddoumi N, Majjaj S, Badran B, Fayyad-Kazan H, Desmedt C, Harris AL *et al*. Global microRNA expression profiling identifies MiR-210 associated with tumor proliferation, invasion and poor clinical outcome in breast cancer. PLoS One 2011, 6(6):e20980.

317. Ying Q, Liang L, Guo W, Zha R, Tian Q, Huang S, Yao J, Ding J, Bao M, Ge C *et al*. Hypoxia-inducible microRNA-210 augments the metastatic potential of tumor cells by targeting vacuole membrane protein 1 in hepatocellular carcinoma. Hepatology 2011, 54(6):2064-2075.

318. Takikawa T, Masamune A, Hamada S, Nakano E, Yoshida N, Shimosegawa T. miR-210 regulates the interaction between pancreatic cancer cells and stellate cells. Biochem Biophys Res Commun 2013.

319. Ellermeier C, Vang S, Cleveland K, Durand W, Resnick MB, Brodsky AS. Prognostic microRNA expression signature from examination of colorectal primary and metastatic tumors. Anticancer Res 2014, 34(8):3957-3967.

320. Redova M, Poprach A, Besse A, Iliev R, Nekvindova J, Lakomy R, Radova L, Svoboda M, Dolezel J, Vyzula R *et al*. MiR-210 expression in tumor tissue and in vitro effects of its silencing in renal cell carcinoma. Tumour Biol 2013, 34(1):481-491.

321. Chen WY, Liu WJ, Zhao YP, Zhou L, Zhang TP, Chen G, Shu H. Induction, modulation and potential targets of miR-210 in pancreatic cancer cells. Hepatobiliary Pancreat Dis Int 2012, 11(3):319-324.

322. Radojicic J, Zaravinos A, Vrekoussis T, Kafousi M, Spandidos DA, Stathopoulos EN. MicroRNA expression analysis in triple-negative (ER, PR and Her2/neu) breast cancer. Cell Cycle 2011, 10(3):507-517.

323. Park JK, Henry JC, Jiang J, Esau C, Gusev Y, Lerner MR, Postier RG, Brackett DJ, Schmittgen TD. miR-132 and miR-212 are increased in pancreatic cancer and target the retinoblastoma tumor suppressor. Biochem Biophys Res Commun 2011, 406(4):518-523.

324. Meng X, Wu J, Pan C, Wang H, Ying X, Zhou Y, Yu H, Zuo Y, Pan Z, Liu RY *et al*. Genetic and Epigenetic Down-regulation of MicroRNA-212 Promotes Colorectal Tumor Metastasis via Dysregulation of MnSOD. Gastroenterology 2013.

325. Wu WY, Xue XY, Chen ZJ, Han SL, Huang YP, Zhang LF, Zhu GB, Shen X. Potentially predictive microRNAs of gastric cancer with metastasis to lymph node. World J Gastroenterol 2011, 17(31):3645-3651.

326. Li Y, Zhang D, Chen C, Ruan Z, Li Y, Huang Y. MicroRNA-212 displays tumor-promoting properties in non-small cell lung cancer cells and targets the hedgehog pathway receptor PTCH1. Mol Biol Cell 2012, 23(8):1423-1434.

327. Incoronato M, Urso L, Portela A, Laukkanen MO, Soini Y, Quintavalle C, Keller S, Esteller M, Condorelli G. Epigenetic regulation of miR-212 expression in lung cancer. PLoS One 2011, 6(11):e27722.

328. Ma C, Nong K, Wu B, Dong B, Bai Y, Zhu H, Wang W, Huang X, Yuan Z, Ai K. miR-212 promotes pancreatic cancer cell growth and invasion by targeting the hedgehog signaling pathway receptor patched-1. J Exp Clin Cancer Res 2014, 33:54.

329. Incoronato M, Garofalo M, Urso L, Romano G, Quintavalle C, Zanca C, Iaboni M, Nuovo G, Croce CM, Condorelli G. miR-212 increases tumor necrosis factor-related apoptosis-inducing ligand sensitivity in non-small cell lung cancer by targeting the antiapoptotic protein PED. Cancer Res 2010, 70(9):3638-3646.

330. Wada R, Akiyama Y, Hashimoto Y, Fukamachi H, Yuasa Y. miR-212 is downregulated and suppresses methyl-CpG-binding protein MeCP2 in human gastric cancer. Int J Cancer 2010, 127(5):1106-1114.

331. Jiping Z, Ming F, Lixiang W, Xiuming L, Yuqun S, Han Y, Zhifang L, Yundong S, Shili L, Chunyan C *et al*. MicroRNA-212 inhibits proliferation of gastric cancer by directly repressing retinoblastoma binding protein 2. J Cell Biochem 2013, 114(12):2666-2672.

332. Qi B, Liu SG, Qin XG, Yao WJ, Lu JG, Guo L, Wang TY, Li HC, Zhao BS. Overregulation of microRNA-212 in the poor prognosis of esophageal cancer patients. Genet Mol Res 2014, 13(3):7800-7807.

333. Endo K, Weng H, Kito N, Fukushima Y, Iwai N. MiR-216a and miR-216b as markers for acute phased pancreatic injury. Biomed Res 2013, 34(4):179-188.

334. Deng M, Tang H, Zhou Y, Zhou M, Xiong W, Zheng Y, Ye Q, Zeng X, Liao Q, Guo X *et al*. miR-216b suppresses tumor growth and invasion by targeting KRAS in nasopharyngeal carcinoma. J Cell Sci 2011, 124(Pt 17):2997-3005.

335. Kim SY, Lee YH, Bae YS. MiR-186, miR-216b, miR-337-3p, and miR-760 cooperatively induce cellular senescence by targeting alpha subunit of protein kinase CKII in human colorectal cancer cells. Biochem Biophys Res Commun 2012, 429(3-4):173-179.

336. Zheng L, Zhang X, Yang F, Zhu J, Zhou P, Yu F, Hou L, Xiao L, He Q, Wang B. Regulation of the P2X7R by microRNA-216b in human breast cancer. Biochem Biophys Res Commun 2014, 452(1):197-204.

337. Li H, Zhao J, Zhang JW, Huang QY, Huang JZ, Chi LS, Tang HJ, Liu GQ, Zhu DJ, Ma WM. MicroRNA-217, down-regulated in clear cell renal cell carcinoma and associated with lower survival, suppresses cell proliferation and migration. Neoplasma 2013, 60(5):511-515.

338. Wang W, Zhao LJ, Tan YX, Ren H, Qi ZT. MiR-138 induces cell cycle arrest by targeting cyclin D3 in hepatocellular carcinoma. Carcinogenesis 2012, 33(5):1113-1120.

339. Zhao WG, Yu SN, Lu ZH, Ma YH, Gu YM, Chen J. The miR-217 microRNA functions as a potential tumor suppressor in pancreatic ductal adenocarcinoma by targeting KRAS. Carcinogenesis 2010, 31(10):1726-1733.

340. Deng S, Zhu S, Wang B, Li X, Liu Y, Qin Q, Gong Q, Niu Y, Xiang C, Chen J *et al*. Chronic pancreatitis and pancreatic cancer demonstrate active epithelial-mesenchymal transition profile, regulated by miR-217-SIRT1 pathway. Cancer Lett 2014, 355(2):184-191.

341. Li Q, Shen K, Zhao Y, He X, Ma C, Wang L, Wang B, Liu J, Ma J. MicroRNA-222 promotes tumorigenesis via targeting DKK2 and activating the Wnt/beta-catenin signaling pathway. FEBS Lett 2013, 587(12):1742-1748.

342. Teixeira AL, Gomes M, Medeiros R. EGFR signaling pathway and related-miRNAs in age-related diseases: the example of miR-221 and miR-222. Front Genet 2012, 3:286.

343. Mardente S, Mari E, Consorti F, Di Gioia C, Negri R, Etna M, Zicari A, Antonaci A. HMGB1 induces the overexpression of miR-222 and miR-221 and increases growth and motility in papillary thyroid cancer cells. Oncol Rep 2012, 28(6):2285-2289.

344. Xu K, Liang X, Shen K, Sun L, Cui D, Zhao Y, Tian J, Ni L, Liu J. MiR-222 modulates multidrug resistance in human colorectal carcinoma by down-regulating ADAM-17. Exp Cell Res 2012, 318(17):2168-2177.

345. Tsunoda T, Takashima Y, Yoshida Y, Doi K, Tanaka Y, Fujimoto T, Machida T, Ota T, Koyanagi M, Kuroki M *et al*. Oncogenic KRAS regulates miR-200c and miR-221/222 in a 3D-specific manner in colorectal cancer cells. Anticancer Res 2011, 31(7):2453-2459.

346. Xiao L, Cui YH, Rao JN, Zou T, Liu L, Smith A, Turner DJ, Gorospe M, Wang JY. Regulation of cyclin-dependent kinase 4 translation through CUG-binding protein 1 and microRNA-222 by polyamines. Mol Biol Cell 2011, 22(17):3055-3069.

347. Galardi S, Mercatelli N, Farace MG, Ciafre SA. NF-kB and c-Jun induce the expression of the oncogenic miR-221 and miR-222 in prostate carcinoma and glioblastoma cells. Nucleic Acids Res 2011, 39(9):3892-3902.

348. Zhang CZ, Zhang JX, Zhang AL, Shi ZD, Han L, Jia ZF, Yang WD, Wang GX, Jiang T, You YP *et al*. MiR-221 and miR-222 target PUMA to induce cell survival in glioblastoma. Mol Cancer 2010, 9:229.

349. Chun-Zhi Z, Lei H, An-Ling Z, Yan-Chao F, Xiao Y, Guang-Xiu W, Zhi-Fan J, Pei-Yu P, Qing-Yu Z, Chun-Sheng K. MicroRNA-221 and microRNA-222 regulate gastric carcinoma cell proliferation and radioresistance by targeting PTEN. BMC Cancer 2010, 10:367.

350. Hwang MS, Yu N, Stinson SY, Yue P, Newman RJ, Allan BB, Dornan D. miR-221/222 Targets Adiponectin Receptor 1 to Promote the Epithelial-to-Mesenchymal Transition in Breast Cancer. PLoS One 2013, 8(6):e66502.

351. Medina R, Zaidi SK, Liu CG, Stein JL, van Wijnen AJ, Croce CM, Stein GS. MicroRNAs 221 and 222 bypass quiescence and compromise cell survival. Cancer Res 2008, 68(8):2773-2780.

352. Zhang C, Han L, Zhang A, Yang W, Zhou X, Pu P, Du Y, Zeng H, Kang C. Global changes of mRNA expression reveals an increased activity of the interferon-induced signal transducer and activator of transcription (STAT) pathway by repression of miR-221/222 in glioblastoma U251 cells. Int J Oncol 2010, 36(6):1503-1512.

353. Stinson S, Lackner MR, Adai AT, Yu N, Kim HJ, O'Brien C, Spoerke J, Jhunjhunwala S, Boyd Z, Januario T *et al*. miR-221/222 targeting of trichorhinophalangeal 1 (TRPS1) promotes epithelial-to-mesenchymal transition in breast cancer. Sci Signal 2011, 4(186):pt5.

354. Falkenberg N, Anastasov N, Rappl K, Braselmann H, Auer G, Walch A, Huber M, Hofig I, Schmitt M, Hofler H *et al*. MiR-221/-222 differentiate prognostic groups in advanced breast cancers and influence cell invasion. Br J Cancer 2013, 109(10):2714-2723.

355. Lee C, He H, Jiang Y, Di Y, Yang F, Li J, Jin C, Fu D. Elevated expression of tumor miR-222 in pancreatic cancer is associated with Ki67 and poor prognosis. Med Oncol 2013, 30(4):700.

356. Poy MN, Hausser J, Trajkovski M, Braun M, Collins S, Rorsman P, Zavolan M, Stoffel M. miR-375 maintains normal pancreatic alpha- and beta-cell mass. Proc Natl Acad Sci U S A 2009, 106(14):5813-5818.

357. Liu AM, Poon RT, Luk JM. MicroRNA-375 targets Hippo-signaling effector YAP in liver cancer and inhibits tumor properties. Biochem Biophys Res Commun 2010, 394(3):623-627.

358. Ding L, Xu Y, Zhang W, Deng Y, Si M, Du Y, Yao H, Liu X, Ke Y, Si J *et al*. MiR-375 frequently downregulated in gastric cancer inhibits cell proliferation by targeting JAK2. Cell Res 2010, 20(7):784-793.

359. Zhao H, Guan J, Lee HM, Sui Y, He L, Siu JJ, Tse PP, Tong PC, Lai FM, Chan JC. Up-regulated pancreatic tissue microRNA-375 associates with human type 2 diabetes through beta-cell deficit and islet amyloid deposition. Pancreas 2010, 39(6):843-846.

360. Szczyrba J, Nolte E, Wach S, Kremmer E, Stohr R, Hartmann A, Wieland W, Wullich B, Grasser FA. Downregulation of Sec23A protein by miRNA-375 in prostate carcinoma. Mol Cancer Res 2011, 9(6):791-800.

361. Nohata N, Hanazawa T, Kikkawa N, Mutallip M, Sakurai D, Fujimura L, Kawakami K, Chiyomaru T, Yoshino H, Enokida H *et al*. Tumor suppressive microRNA-375 regulates oncogene AEG-1/MTDH in head and neck squamous cell carcinoma (HNSCC). J Hum Genet 2011, 56(8):595-601.

362. Li X, Lin R, Li J. Epigenetic silencing of microRNA-375 regulates PDK1 expression in esophageal cancer. Dig Dis Sci 2011, 56(10):2849-2856.

363. Zhou J, Song S, Cen J, Zhu D, Li D, Zhang Z. MicroRNA-375 is downregulated in pancreatic cancer and inhibits cell proliferation in vitro. Oncol Res 2012, 20(5-6):197-203.

364. Li Y, Jiang Q, Xia N, Yang H, Hu C. Decreased expression of microRNA-375 in nonsmall cell lung cancer and its clinical significance. J Int Med Res 2012, 40(5):1662-1669.

365. Kong KL, Kwong DL, Chan TH, Law SY, Chen L, Li Y, Qin YR, Guan XY. MicroRNA-375 inhibits tumour growth and metastasis in oesophageal squamous cell carcinoma through repressing insulin-like growth factor 1 receptor. Gut 2012, 61(1):33-42.

366. Dai X, Chiang Y, Wang Z, Song Y, Lu C, Gao P, Xu H. Expression levels of microRNA-375 in colorectal carcinoma. Mol Med Rep 2012, 5(5):1299-1304.

367. Bierkens M, Krijgsman O, Wilting SM, Bosch L, Jaspers A, Meijer GA, Meijer CJ, Snijders PJ, Ylstra B, Steenbergen RD. Focal aberrations indicate EYA2 and hsa-miR-375 as oncogene and tumor suppressor in cervical carcinogenesis. Genes Chromosomes Cancer 2013, 52(1):56-68.

368. Wang Y, Tang Q, Li M, Jiang S, Wang X. MicroRNA-375 inhibits colorectal cancer growth by targeting PIK3CA. Biochem Biophys Res Commun 2014, 444(2):199-204.

369. Harris T, Jimenez L, Kawachi N, Fan JB, Chen J, Belbin T, Ramnauth A, Loudig O, Keller CE, Smith R *et al*. Low-level expression of miR-375 correlates with poor outcome and metastasis while altering the invasive properties of head and neck squamous cell carcinomas. Am J Pathol 2012, 180(3):917-928.

370. Ma MZ, Kong X, Weng MZ, Cheng K, Gong W, Quan ZW, Peng CH. Candidate microRNA biomarkers of pancreatic ductal adenocarcinoma: meta-analysis, experimental validation and clinical significance. J Exp Clin Cancer Res 2013, 32:71.

371. Song SD, Zhou J, Zhou J, Zhao H, Cen JN, Li DC. MicroRNA-375 targets the 3-phosphoinositide-dependent protein kinase-1 gene in pancreatic carcinoma. Oncol Lett 2013, 6(4):953-959.

372. Fu C, Dong W, Wang Z, Li H, Qin Q, Li B. The expression of miR-21 and miR-375 predict prognosis of esophageal cancer. Biochem Biophys Res Commun 2014, 446(4):1197-1203.

373. Shen ZY, Zhang ZZ, Liu H, Zhao EH, Cao H. miR-375 inhibits the proliferation of gastric cancer cells by repressing ERBB2 expression. Exp Ther Med 2014, 7(6):1757-1761.

374. van Schooneveld E, Wouters MC, Van der Auwera I, Peeters DJ, Wildiers H, Van Dam PA, Vergote I, Vermeulen PB, Dirix LY, Van Laere SJ. Expression profiling of cancerous and normal breast tissues identifies microRNAs that are differentially expressed in serum from patients with (metastatic) breast cancer and healthy volunteers. Breast Cancer Res 2012, 14(1):R34.

375. Skalsky RL, Cullen BR. Reduced expression of brain-enriched microRNAs in glioblastomas permits targeted regulation of a cell death gene. PLoS One 2011, 6(9):e24248.

376. Kim YW, Kim EY, Jeon D, Liu JL, Kim HS, Choi JW, Ahn WS. Differential microRNA expression signatures and cell type-specific association with Taxol resistance in ovarian cancer cells. Drug Des Devel Ther 2014, 8:293-314.

377. Nadal E, Zhong J, Lin J, Reddy RM, Ramnath N, Orringer MB, Chang AC, Beer DG, Chen G. A MicroRNA cluster at 14q32 drives aggressive lung adenocarcinoma. Clin Cancer Res 2014, 20(12):3107-3117.

378. Torres-Martin M, Lassaletta L, de Campos JM, Isla A, Gavilan J, Pinto GR, Burbano RR, Latif F, Melendez B, Castresana JS *et al*. Global profiling in vestibular schwannomas shows critical deregulation of microRNAs and upregulation in those included in chromosomal region 14q32. PLoS One 2013, 8(6):e65868.

379. Kanaan Z, Roberts H, Eichenberger MR, Billeter A, Ocheretner G, Pan J, Rai SN, Jorden J, Williford A, Galandiuk S. A plasma microRNA panel for detection of colorectal adenomas: a step toward more precise screening for colorectal cancer. Ann Surg 2013, 258(3):400-408.

380. Tanaka T, Arai M, Jiang X, Sugaya S, Kanda T, Fujii K, Kita K, Sugita K, Imazeki F, Miyashita T *et al*. Downregulation of microRNA-431 by human interferon-beta inhibits viability of medulloblastoma and glioblastoma cells via upregulation of SOCS6. Int J Oncol 2014, 44(5):1685-1690.

381. Sun MM, Li JF, Guo LL, Xiao HT, Dong L, Wang F, Huang FB, Cao D, Qin T, Yin XH *et al*. TGF-beta1 suppression of microRNA-450b-5p expression: a novel mechanism for blocking myogenic differentiation of rhabdomyosarcoma. Oncogene 2013.

382. Izzotti A, Larghero P, Longobardi M, Cartiglia C, Camoirano A, Steele VE, De Flora S. Dose-responsiveness and persistence of microRNA expression alterations induced by cigarette smoke in mouse lung. Mutat Res 2011, 717(1-2):9-16.

383. Pan X, Wang R, Wang ZX. The Potential Role of miR-451 in Cancer Diagnosis, Prognosis, and Therapy. Mol Cancer Ther 2013, 12(7):1153-1162.

384. Ali S, Saleh H, Sethi S, Sarkar FH, Philip PA. MicroRNA profiling of diagnostic needle aspirates from patients with pancreatic cancer. Br J Cancer 2012, 107(8):1354-1360.

385. Ng EK, Li R, Shin VY, Jin HC, Leung CP, Ma ES, Pang R, Chua D, Chu KM, Law WL *et al*. Circulating microRNAs as specific biomarkers for breast cancer detection. PLoS One 2013, 8(1):e53141.

386. Redova M, Poprach A, Nekvindova J, Iliev R, Radova L, Lakomy R, Svoboda M, Vyzula R, Slaby O. Circulating miR-378 and miR-451 in serum are potential biomarkers for renal cell carcinoma. J Transl Med 2012, 10:55.

387. Bitarte N, Bandres E, Boni V, Zarate R, Rodriguez J, Gonzalez-Huarriz M, Lopez I, Javier Sola J, Alonso MM, Fortes P *et al*. MicroRNA-451 is involved in the self-renewal, tumorigenicity, and chemoresistance of colorectal cancer stem cells. Stem Cells 2011, 29(11):1661-1671.

388. Bandres E, Bitarte N, Arias F, Agorreta J, Fortes P, Agirre X, Zarate R, Diaz-Gonzalez JA, Ramirez N, Sola JJ *et al*. microRNA-451 regulates macrophage migration inhibitory factor production and proliferation of gastrointestinal cancer cells. Clin Cancer Res 2009, 15(7):2281-2290.

389. Chen MB, Wei MX, Han JY, Wu XY, Li C, Wang J, Shen W, Lu PH. MicroRNA-451 regulates AMPK/mTORC1 signaling and fascin1 expression in HT-29 colorectal cancer. Cell Signal 2014, 26(1):102-109.

390. Chen D, Huang J, Zhang K, Pan B, Chen J, De W, Wang R, Chen L. MicroRNA-451 induces epithelial-mesenchymal transition in docetaxel-resistant lung adenocarcinoma cells by targeting proto-oncogene c-Myc. Eur J Cancer 2014, 50(17):3050-3067.

391. Slattery ML, Herrick JS, Mullany LE, Valeri N, Stevens J, Caan BJ, Samowitz W, Wolff RK. An evaluation and replication of miRNAs with disease stage and colorectal cancer-specific mortality. Int J Cancer 2014.

392. Wang J, Paris PL, Chen J, Ngo V, Yao H, Frazier ML, Killary AM, Liu CG, Liang H, Mathy C *et al*. Next generation sequencing of pancreatic cyst fluid microRNAs from low grade-benign and high grade-invasive lesions. Cancer Lett 2015, 356(2 Pt B):404-409.

393. Zhang LY, Liu M, Li X, Tang H. miR-490-3p modulates cell growth and epithelial to mesenchymal transition of hepatocellular carcinoma cells by targeting endoplasmic reticulum-Golgi intermediate compartment protein 3 (ERGIC3). J Biol Chem 2013, 288(6):4035-4047.

394. Li S, Xu X, Xu X, Hu Z, Wu J, Zhu Y, Chen H, Mao Y, Lin Y, Luo J *et al*. MicroRNA-490-5p inhibits proliferation of bladder cancer by targeting c-Fos. Biochem Biophys Res Commun 2013, 441(4):976-981.

395. Gu H, Yang T, Fu S, Chen X, Guo L, Ni Y. MicroRNA-490-3p inhibits proliferation of A549 lung cancer cells by targeting CCND1. Biochem Biophys Res Commun 2014, 444(1):104-108.

396. Hamfjord J, Stangeland AM, Hughes T, Skrede ML, Tveit KM, Ikdahl T, Kure EH. Differential expression of miRNAs in colorectal cancer: comparison of paired tumor tissue and adjacent normal mucosa using high-throughput sequencing. PLoS One 2012, 7(4):e34150.

397. Wojcicka A, Swierniak M, Kornasiewicz O, Gierlikowski W, Maciag M, Kolanowska M, Kotlarek M, Gornicka B, Koperski L, Niewinski G *et al*. Next generation sequencing reveals microRNA isoforms in liver cirrhosis and hepatocellular carcinoma. Int J Biochem Cell Biol 2014, 53:208-217.

398. Shen J, Xiao Z, Wu WK, Wang MH, To KF, Chen Y, Yang W, Li MS, Shin VY, Tong JH *et al*. Epigenetic Silencing of miR-490-3p Reactivates the Chromatin Remodeler SMARCD1 to Promote Helicobacter pylori-Induced Gastric Carcinogenesis. Cancer Res 2015, 75(4):754-765.

399. Gaedcke J, Grade M, Camps J, Sokilde R, Kaczkowski B, Schetter AJ, Difilippantonio MJ, Harris CC, Ghadimi BM, Moller S *et al*. The rectal cancer microRNAome--microRNA expression in rectal cancer and matched normal mucosa. Clin Cancer Res 2012, 18(18):4919-4930.

400. von Frowein J, Pagel P, Kappler R, von Schweinitz D, Roscher A, Schmid I. MicroRNA-492 is processed from the keratin 19 gene and up-regulated in metastatic hepatoblastoma. Hepatology 2011, 53(3):833-842.

401. Patella F, Leucci E, Evangelista M, Parker B, Wen J, Mercatanti A, Rizzo M, Chiavacci E, Lund AH, Rainaldi G. MiR-492 impairs the angiogenic potential of endothelial cells. J Cell Mol Med 2013, 17(8):1006-1015.

402. Jiang J, Zhang Y, Yu C, Li Z, Pan Y, Sun C. MicroRNA-492 expression promotes the progression of hepatic cancer by targeting PTEN. Cancer Cell Int 2014, 14(1):95.

403. Lin MS, Chen WC, Huang JX, Gao HJ, Sheng HH. Aberrant expression of microRNAs in serum may identify individuals with pancreatic cancer. Int J Clin Exp Med 2014, 7(12):5226-5234.

404. Xing F, Sharma S, Liu Y, Mo YY, Wu K, Zhang YY, Pochampally R, Martinez LA, Lo HW, Watabe K. miR-509 suppresses brain metastasis of breast cancer cells by modulating RhoC and TNF-alpha. Oncogene 2015.

405. Han Z, Yang Q, Liu B, Wu J, Li Y, Yang C, Jiang Y. MicroRNA-622 functions as a tumor suppressor by targeting K-Ras and enhancing the anticarcinogenic effect of resveratrol. Carcinogenesis 2012, 33(1):131-139.

406. Guo XB, Jing CQ, Li LP, Zhang L, Shi YL, Wang JS, Liu JL, Li CS. Down-regulation of miR-622 in gastric cancer promotes cellular invasion and tumor metastasis by targeting ING1 gene. World J Gastroenterol 2011, 17(14):1895-1902.

407. Zhang R, Luo H, Wang S, Chen Z, Hua L, Wang HW, Chen W, Yuan Y, Zhou X, Li D *et al*. miR-622 suppresses proliferation, invasion and migration by directly targeting activating transcription factor 2 in glioma cells. J Neurooncol 2015, 121(1):63-72.

408. Wang M, Li C, Nie H, Lv X, Qu Y, Yu B, Su L, Li J, Chen X, Ju J *et al*. Down-regulated miR-625 suppresses invasion and metastasis of gastric cancer by targeting ILK. FEBS Lett 2012, 586(16):2382-2388.

409. Roth C, Stuckrath I, Pantel K, Izbicki JR, Tachezy M, Schwarzenbach H. Low levels of cell-free circulating miR-361-3p and miR-625* as blood-based markers for discriminating malignant from benign lung tumors. PLoS One 2012, 7(6):e38248.

410. Rasmussen MH, Jensen NF, Tarpgaard LS, Qvortrup C, Romer MU, Stenvang J, Hansen TP, Christensen LL, Lindebjerg J, Hansen F *et al*. High expression of microRNA-625-3p is associated with poor response to first-line oxaliplatin based treatment of metastatic colorectal cancer. Mol Oncol 2013, 7(3):637-646.

411. Lou X, Qi X, Zhang Y, Long H, Yang J. Decreased expression of microRNA-625 is associated with tumor metastasis and poor prognosis in patients with colorectal cancer. J Surg Oncol 2013, 108(4):230-235.

412. Wang Z, Qiao Q, Chen M, Li X, Wang Z, Liu C, Xie Z. miR-625 down-regulation promotes proliferation and invasion in esophageal cancer by targeting Sox2. FEBS Lett 2014, 588(6):915-921.

413. Katayama Y, Maeda M, Miyaguchi K, Nemoto S, Yasen M, Tanaka S, Mizushima H, Fukuoka Y, Arii S, Tanaka H. Identification of pathogenesis-related microRNAs in hepatocellular carcinoma by expression profiling. Oncol Lett 2012, 4(4):817-823.

414. Gits CM, van Kuijk PF, de Rijck JC, Muskens N, Jonkers MB, van IWF, Mathijssen RH, Verweij J, Sleijfer S, Wiemer EA. MicroRNA response to hypoxic stress in soft tissue sarcoma cells: microRNA mediated regulation of HIF3alpha. BMC Cancer 2014, 14:429.

415. Zhou X, Zhang CZ, Lu SX, Chen GG, Li LZ, Liu LL, Yi C, Fu J, Hu W, Wen JM *et al*. miR-625 suppresses tumour migration and invasion by targeting IGF2BP1 in hepatocellular carcinoma. Oncogene 2015, 34(8):965-977.

416. Tsang WP, Ng EK, Ng SS, Jin H, Yu J, Sung JJ, Kwok TT. Oncofetal H19-derived miR-675 regulates tumor suppressor RB in human colorectal cancer. Carcinogenesis 2010, 31(3):350-358.

417. Gibson G, Asahara H. microRNAs and cartilage. J Orthop Res 2013, 31(9):1333-1344.

418. Keniry A, Oxley D, Monnier P, Kyba M, Dandolo L, Smits G, Reik W. The H19 lincRNA is a developmental reservoir of miR-675 that suppresses growth and Igf1r. Nat Cell Biol 2012, 14(7):659-665.

419. Hernandez JM, Elahi A, Clark CW, Wang J, Humphries LA, Centeno B, Bloom G, Fuchs BC, Yeatman T, Shibata D. miR-675 mediates downregulation of Twist1 and Rb in AFP-secreting hepatocellular carcinoma. Ann Surg Oncol 2013, 20 Suppl 3:S625-635.

420. Zhuang M, Gao W, Xu J, Wang P, Shu Y. The long non-coding RNA H19-derived miR-675 modulates human gastric cancer cell proliferation by targeting tumor suppressor RUNX1. Biochem Biophys Res Commun 2014, 448(3):315-322.

421. Zhu M, Chen Q, Liu X, Sun Q, Zhao X, Deng R, Wang Y, Huang J, Xu M, Yan J *et al*. lncRNA H19/miR-675 axis represses prostate cancer metastasis by targeting TGFBI. FEBS J 2014, 281(16):3766-3775.

422. Kallen AN, Zhou XB, Xu J, Qiao C, Ma J, Yan L, Lu L, Liu C, Yi JS, Zhang H *et al*. The imprinted H19 lncRNA antagonizes let-7 microRNAs. Mol Cell 2013, 52(1):101-112.

423. Matouk IJ, Raveh E, Abu-lail R, Mezan S, Gilon M, Gershtain E, Birman T, Gallula J, Schneider T, Barkali M *et al*. Oncofetal H19 RNA promotes tumor metastasis. Biochim Biophys Acta 2014, 1843(7):1414-1426.

424. Luo EC, Chang YC, Sher YP, Huang WY, Chuang LL, Chiu YC, Tsai MH, Chuang EY, Lai LC. MicroRNA-769-3p down-regulates NDRG1 and enhances apoptosis in MCF-7 cells during reoxygenation. Sci Rep 2014, 4:5908.

425. Nymark P, Guled M, Borze I, Faisal A, Lahti L, Salmenkivi K, Kettunen E, Anttila S, Knuutila S. Integrative analysis of microRNA, mRNA and aCGH data reveals asbestos- and histology-related changes in lung cancer. Genes Chromosomes Cancer 2011, 50(8):585-597.

426. Christensen LL, Tobiasen H, Holm A, Schepeler T, Ostenfeld MS, Thorsen K, Rasmussen MH, Birkenkamp-Demtroeder K, Sieber OM, Gibbs P *et al*. MiRNA-362-3p induces cell cycle arrest through targeting of E2F1, USF2 and PTPN1 and is associated with recurrence of colorectal cancer. Int J Cancer 2013, 133(1):67-78.

427. Ma J, Mannoor K, Gao L, Tan A, Guarnera MA, Zhan M, Shetty A, Stass SA, Xing L, Jiang F. Characterization of microRNA transcriptome in lung cancer by next-generation deep sequencing. Mol Oncol 2014, 8(7):1208-1219.

428. Xie H, Lee L, Scicluna P, Kavak E, Larsson C, Sandberg R, Lui WO. Novel functions and targets of miR-944 in human cervical cancer cells. Int J Cancer 2015, 136(5):E230-241.
