# Supplementary material for: Tissue MicroRNA profiles as diagnostic and prognostic biomarkers in patients with resectable pancreatic ductal adenocarcinoma and periampullary cancers
Source: Biomark Res. 2017 Feb 21;5:8. doi: 10.1186/s40364-017-0087-6 (PMC5320745; doi:10.1186/s40364-017-0087-6)
Supplement: Additional file 2: — All statistical calculations including insignificant results not presented in the manuscript. (DOC 1481 kb) [file 40364_2017_87_MOESM2_ESM.doc]

**Supplementary**

This is an additional file for the paper: *”Tissue MicroRNA Profiles as Diagnostic and Prognostic Biomarkers in Patients with Resectable Pancreatic Ductal Adenocarcinoma and Periampullary Cancers”* by Dan Calatayud et al.

**DIAGNOSTIC CALCULATIONS**

In this section we consider the diagnosis. Two types of comparisons are made PC vs. HS and PC vs. HS+CP. All analyses are based on logistic regression models, i.e., models where Diagnosis=PDAC is considered the event.

**Univariate analysis**

In this part we consider the univariate effects of the miRNA. All effects are reported as 1 unit increase effects and OR>1 implies that decreased expression increases the risk of cancer. All analyses are made on the *raw*values. Missing values are excluded.

**PC vs. HS**

**Univariate effects for Cancer vs. HS**

| **miRNA** | **OR** | **ORlow** | **ORhigh** | **pval** | **PC** | **Healthy** | **NAs** |
| --- | --- | --- | --- | --- | --- | --- | --- |
| miR-31-5p | 0.3836 | 0.2768 | 0.4997 | 0.0000 | 165 | 35 | 0 |
| miR-130b-3p | 5.3413 | 3.1748 | 9.9794 | 0.0000 | 165 | 35 | 0 |
| miR-375 | 26.0992 | 9.4806 | 90.2170 | 0.0000 | 165 | 35 | 0 |
| miR-93-3p | 0.1365 | 0.0634 | 0.2574 | 0.0000 | 165 | 34 | 1 |
| miR-210 | 0.1162 | 0.0471 | 0.2248 | 0.0000 | 165 | 34 | 1 |
| miR-217 | 2.9381 | 2.0327 | 4.6880 | 0.0000 | 142 | 35 | 23 |
| miR-216b | 6.2965 | 3.3634 | 14.2377 | 0.0000 | 149 | 35 | 16 |
| miR-155-5p | 0.1098 | 0.0348 | 0.2274 | 0.0000 | 165 | 33 | 2 |
| miR-21-5p | 0.1054 | 0.0306 | 0.2459 | 0.0000 | 134 | 13 | 53 |
| miR-205-5p | 0.7081 | 0.5932 | 0.8248 | 0.0000 | 148 | 21 | 31 |
| miR-944 | 0.4952 | 0.1671 | 1.1420 | 0.1377 | 46 | 3 | 151 |
| miR-411 | 1.0702 | 0.8708 | 1.3536 | 0.5440 | 165 | 35 | 0 |
| miR-34c-5p | 0.1732 | 0.0896 | 0.2840 | 0.0000 | 165 | 35 | 0 |
| miR-135b-3p | 0.3111 | 0.2005 | 0.4354 | 0.0000 | 165 | 30 | 5 |
| miR-203 | 0.3677 | 0.2481 | 0.5091 | 0.0000 | 165 | 35 | 0 |
| miR-222-3p | 0.0623 | 0.0181 | 0.1477 | 0.0000 | 165 | 35 | 0 |
| miR-451 | 0.1412 | 0.0561 | 0.2660 | 0.0000 | 165 | 35 | 0 |
| miR-622 | 0.5680 | 0.4129 | 0.7613 | 0.0003 | 165 | 34 | 1 |
| miR-122-5p | 2.0750 | 1.4040 | 3.5068 | 0.0014 | 30 | 18 | 152 |
| miR-23a-3p | 0.3601 | 0.1327 | 0.6679 | 0.0100 | 156 | 5 | 39 |
| miR-196b-5p | 0.1406 | 0.0154 | 0.4508 | 0.0151 | 147 | 3 | 50 |
| miR-186-5p | 0.7499 | 0.5486 | 1.0191 | 0.0664 | 165 | 35 | 0 |
| miR-490-3p | 0.4186 | 0.1024 | 1.1979 | 0.1329 | 41 | 2 | 157 |
| miR-136-3p | 1.1811 | 0.9183 | 1.5344 | 0.2013 | 157 | 34 | 9 |
| miR-198 | 0.6193 | 0.1974 | 1.6383 | 0.3463 | 95 | 2 | 103 |
| miR-614 | 0.9029 | 0.5841 | 1.3541 | 0.6412 | 110 | 3 | 87 |
| miR-509-5p | 0.9243 | 0.5546 | 1.4530 | 0.7332 | 11 | 4 | 185 |
| miR-492 | 0.0000 | 0.0000 | Inf | 0.9990 | 57 | 1 | 142 |

**PC vs. HS+CP**

**Univariate effects for Cancer vs. HS+CP**

| **miRNA** | **OR** | **ORlow** | **ORhigh** | **pval** | **PC** | **Healthy** | **NAs** |
| --- | --- | --- | --- | --- | --- | --- | --- |
| miR-210 | 0.2662 | 0.1840 | 0.3630 | 0.0000 | 165 | 73 | 1 |
| miR-155-5p | 0.4622 | 0.3736 | 0.5579 | 0.0000 | 165 | 72 | 2 |
| miR-31-5p | 0.4972 | 0.4075 | 0.5916 | 0.0000 | 165 | 74 | 0 |
| miR-93-3p | 0.2686 | 0.1743 | 0.3956 | 0.0000 | 165 | 73 | 1 |
| miR-21-5p | 0.2356 | 0.1430 | 0.3580 | 0.0000 | 134 | 42 | 63 |
| miR-216b | 1.5534 | 1.3438 | 1.8436 | 0.0000 | 149 | 73 | 17 |
| miR-217 | 1.4597 | 1.2837 | 1.6947 | 0.0000 | 142 | 71 | 26 |
| miR-375 | 2.2158 | 1.6204 | 3.1512 | 0.0000 | 165 | 74 | 0 |
| miR-205-5p | 0.7944 | 0.7081 | 0.8817 | 0.0000 | 148 | 46 | 45 |
| miR-130b-3p | 1.7058 | 1.3307 | 2.2324 | 0.0001 | 165 | 74 | 0 |
| miR-944 | 0.6126 | 0.3472 | 0.9863 | 0.0610 | 46 | 9 | 184 |
| miR-411 | 1.0378 | 0.8917 | 1.2219 | 0.6414 | 165 | 74 | 0 |
| miR-222-3p | 0.2313 | 0.1564 | 0.3232 | 0.0000 | 165 | 74 | 0 |
| miR-34c-5p | 0.3338 | 0.2469 | 0.4334 | 0.0000 | 165 | 74 | 0 |
| miR-451 | 0.4430 | 0.3517 | 0.5397 | 0.0000 | 165 | 74 | 0 |
| miR-135b-3p | 0.3098 | 0.2171 | 0.4129 | 0.0000 | 165 | 58 | 16 |
| miR-203 | 0.3551 | 0.2617 | 0.4618 | 0.0000 | 165 | 74 | 0 |
| miR-622 | 0.5248 | 0.4062 | 0.6616 | 0.0000 | 165 | 72 | 2 |
| miR-196b-5p | 0.5357 | 0.3890 | 0.7027 | 0.0000 | 147 | 20 | 72 |
| miR-122-5p | 1.9895 | 1.4641 | 2.9820 | 0.0001 | 30 | 40 | 169 |
| miR-23a-3p | 0.5408 | 0.3821 | 0.7405 | 0.0003 | 156 | 31 | 52 |
| miR-186-5p | 0.7051 | 0.5514 | 0.8911 | 0.0041 | 165 | 74 | 0 |
| miR-492 | 0.4565 | 0.2191 | 0.7761 | 0.0097 | 57 | 4 | 178 |
| miR-614 | 0.7474 | 0.5675 | 0.9415 | 0.0219 | 110 | 14 | 115 |
| miR-136-3p | 1.1556 | 0.9536 | 1.4073 | 0.1439 | 157 | 73 | 9 |
| miR-490-3p | 0.7002 | 0.3564 | 1.3141 | 0.2713 | 41 | 6 | 192 |
| miR-198 | 1.1251 | 0.8263 | 1.5425 | 0.4553 | 95 | 22 | 122 |
| miR-509-5p | 0.9607 | 0.6061 | 1.4820 | 0.8530 | 11 | 5 | 223 |

**Diagnostic indices**

The new diagnostic indices constructed in this paper are thoroughly and their performance is described in the paper and will not be touched further in this supplementary.

In the current paper we also tested 4 previously described indices (Schultz et al. MicroRNA expression profiles associated with pancreatic adenocarcinoma and ampullary adenocarcinoma. Mod Pathol 2012, 25(12):1609-1622)

In the following 4 indexes:

1. miR-196b-5p – miR-217

2. miR-411 – miR-198

3. miR-614 – miR-122-5p

4. miR-614 – miR-93-3p

For these indexes we only use observations, which are non missing (i.e. not imputed by a large CT-value). It should be noted that index 1 has 97 samples with at least one of the miRNAs missing, index 2 contains 122 samples with at least one of the miRNAs missing, index 3 contains 213 samples with at least one of the miRNAs missing and index 4 contains 115 samples with at least one of the miRNAs missing. For index 2 and 3 it is not possible to consider HS alone.

Missing values according to diagnosis can be seen in the tables below:

Missing values (TRUE=missing) for index 1 vs. diagnosis

|  | FALSE | TRUE |
| --- | --- | --- |
| PDAC | 125 | 40 |
| GC | 10 | 10 |
| A-AC | 38 | 21 |
| DC | 5 | 1 |
| CBD Cancer | 16 | 5 |
| Healthy | 3 | 32 |
| Chronic pancreatitis | 14 | 25 |

Missing values (TRUE=missing) for index 2 vs. Diagnosis

|  | FALSE | TRUE |
| --- | --- | --- |
| PDAC | 95 | 70 |
| GC | 15 | 5 |
| A-AC | 28 | 31 |
| DC | 2 | 4 |
| CBD Cancer | 10 | 11 |
| Healthy | 2 | 33 |
| Chronic pancreatitis | 20 | 19 |

Missing values (TRUE=missing) for index 3 vs. Diagnosis

|  | FALSE | TRUE |
| --- | --- | --- |
| PDAC | 20 | 145 |
| GC | 1 | 19 |
| A-AC | 3 | 56 |
| DC | 0 | 6 |
| CBD Cancer | 3 | 18 |
| Healthy | 1 | 34 |
| Chronic pancreatitis | 5 | 34 |

Missing values (TRUE=missing) for index 4 vs. diagnosis

|  | FALSE | TRUE |
| --- | --- | --- |
| PDAC | 110 | 55 |
| GC | 15 | 5 |
| A-AC | 34 | 25 |
| DC | 3 | 3 |
| CBD Cancer | 12 | 9 |
| Healthy | 3 | 32 |
| Chronic pancreatitis | 11 | 28 |

**PROGNOSTIC CALCULATIONS**

**Prognosis on diagnosis: PDAC and A-AC combined**

In the following miRNAs univariately are considered.

Unadjusted univariate effects

| **miRNA** | **HR** | **HRlow** | **HRhigh** | **P** | **N** |
| --- | --- | --- | --- | --- | --- |
| let-7g | 0.9023 | 0.8053 | 1.0109 | 0.0763 | 156 |
| miR-29a-5p | 0.8676 | 0.7631 | 0.9865 | 0.0302 | 156 |
| miR-34a-5p | 0.6612 | 0.5405 | 0.8088 | *<*0.0001 | 156 |
| miR-125a-3p | 0.8308 | 0.7296 | 0.9459 | 0.0051 | 153 |
| miR-146a-5p | 0.8651 | 0.7592 | 0.9857 | 0.0296 | 157 |
| miR-148a | 0.9998 | 0.9229 | 1.0831 | 0.9959 | 156 |
| miR-187 | 1.2076 | 0.9589 | 1.5208 | 0.1089 | 47 |
| miR-194-3p | 1.2224 | 0.8979 | 1.6643 | 0.2021 | 50 |
| miR-205-5p | 0.9102 | 0.8637 | 0.9593 | 4e-04 | 130 |
| miR-212-3p | 0.8063 | 0.7156 | 0.9085 | 4e-04 | 156 |
| miR-222-5p | 0.8947 | 0.7799 | 1.0265 | 0.1126 | 152 |
| miR-431-5p | 0.9087 | 0.7023 | 1.1756 | 0.4661 | 47 |
| miR-450b-5p | 0.8779 | 0.7289 | 1.0574 | 0.1701 | 103 |
| miR-625-5p | 1.0533 | 0.9220 | 1.2033 | 0.4448 | 79 |
| miR-675-5p | 0.8115 | 0.3454 | 1.9063 | 0.6316 | 8 |
| miR-769-5p | 0.9730 | 0.8001 | 1.1833 | 0.7841 | 155 |
| miR-944 | 0.9425 | 0.7725 | 1.1500 | 0.56 | 38 |

Adjusted (age, sex, tumor stage, ASA-score, tumor differentiation) univariate effects

| **miRNA** | **HR** | **HRlow** | **HRhigh** | **P** | **N** |
| --- | --- | --- | --- | --- | --- |
| let-7g | 0.9025 | 0.8019 | 1.0157 | 0.0889 | 145 |
| miR-29a-5p | 0.8507 | 0.7413 | 0.9761 | 0.0212 | 145 |
| miR-34a-5p | 0.6419 | 0.5194 | 0.7933 | *<*0.0001 | 145 |
| miR-125a-3p | 0.8292 | 0.7225 | 0.9518 | 0.0077 | 142 |
| miR-146a-5p | 0.8461 | 0.7357 | 0.9731 | 0.0191 | 146 |
| miR-148a | 0.9902 | 0.91 | 1.0775 | 0.82 | 145 |
| miR-187 | 1.2907 | 0.9426 | 1.7673 | 0.1116 | 44 |
| miR-194-3p | 1.1604 | 0.7943 | 1.6952 | 0.4417 | 46 |
| miR-205-5p | 0.9205 | 0.8704 | 0.9735 | 0.0037 | 120 |
| miR-212-3p | 0.8005 | 0.7078 | 0.9055 | 4e-04 | 145 |
| miR-222-5p | 0.8913 | 0.7801 | 1.0183 | 0.0905 | 141 |
| miR-431-5p | 0.8828 | 0.6168 | 1.2636 | 0.4958 | 42 |
| miR-450b-5p | 0.8384 | 0.6678 | 1.0526 | 0.1289 | 93 |
| miR-625-5p | 1.075 | 0.9245 | 1.2501 | 0.3474 | 74 |
| miR-675-5p | 0.262 | 0.0231 | 2.9676 | 0.2795 | 7 |
| miR-769-5p | 1.0108 | 0.8265 | 1.2361 | 0.9167 | 144 |
| miR-944 | 0.8742 | 0.6512 | 1.1735 | 0.3708 | 35 |

Unadjusted univariate effects (under median vs. over median)

| **miRNA** | **HR** | **HRlow** | **HRhigh** | **P** | **N** |
| --- | --- | --- | --- | --- | --- |
| let-7g | 0.6923 | 0.4745 | 1.0100 | 0.0564 | 156 |
| miR-29a-5p | 0.7046 | 0.4841 | 1.0254 | 0.0674 | 156 |
| miR-34a-5p | 0.4550 | 0.3095 | 0.6690 | *<*0.0001 | 156 |
| miR-125a-3p | 0.7019 | 0.4807 | 1.0248 | 0.0668 | 153 |
| miR-146a-5p | 0.7712 | 0.5310 | 1.1201 | 0.1725 | 157 |
| miR-148a | 1.2243 | 0.8419 | 1.7804 | 0.2894 | 156 |
| miR-187 | 1.5273 | 0.7496 | 3.1116 | 0.2435 | 47 |
| miR-194-3p | 1.3276 | 0.6113 | 2.8834 | 0.4739 | 50 |
| miR-205-5p | 0.3730 | 0.2454 | 0.5672 | *<*0.0001 | 130 |
| miR-212-3p | 0.5061 | 0.3459 | 0.7407 | 5e-04 | 156 |
| miR-222-5p | 0.6846 | 0.4691 | 0.9991 | 0.0495 | 152 |
| miR-431-5p | 0.9461 | 0.4960 | 1.8046 | 0.8664 | 47 |
| miR-450b-5p | 1.0538 | 0.6713 | 1.6544 | 0.8198 | 103 |
| miR-625-5p | 1.2485 | 0.7374 | 2.1137 | 0.4087 | 79 |
| miR-675-5p | 0.8872 | 0.1728 | 4.5568 | 0.886 | 8 |
| miR-769-5p | 0.8474 | 0.5838 | 1.2299 | 0.3836 | 155 |
| miR-944 | 1.0641 | 0.5341 | 2.1204 | 0.8597 | 38 |

Adjusted (age, sex, tumor stage, ASA-score, tumor differentiation) univariate effects (under median vs. over median)

| **miRNA** | **HR** | **HRlow** | **HRhigh** | **P** | **N** |
| --- | --- | --- | --- | --- | --- |
| let-7g | 0.62 | 0.4118 | 0.9334 | 0.0220 | 145 |
| miR-29a-5p | 0.637 | 0.4224 | 0.9606 | 0.0314 | 145 |
| miR-34a-5p | 0.4654 | 0.3064 | 0.7067 | 0.0003 | 145 |
| miR-125a-3p | 0.661 | 0.4347 | 1.005 | 0.0528 | 142 |
| miR-146a-5p | 0.722 | 0.4776 | 1.0914 | 0.1224 | 146 |
| miR-148a | 1.1773 | 0.7859 | 1.7636 | 0.4287 | 145 |
| miR-187 | 1.5748 | 0.6677 | 3.7142 | 0.2996 | 44 |
| miR-194-3p | 1.229 | 0.4757 | 3.1747 | 0.6703 | 46 |
| miR-205-5p | 0.4397 | 0.281 | 0.6882 | 0.0003 | 120 |
| miR-212-3p | 0.5283 | 0.3517 | 0.7936 | 0.0021 | 145 |
| miR-222-5p | 0.6752 | 0.4533 | 1.0057 | 0.0534 | 141 |
| miR-431-5p | 0.8936 | 0.3552 | 2.2481 | 0.8111 | 42 |
| miR-450b-5p | 0.8952 | 0.5249 | 1.5268 | 0.6844 | 93 |
| miR-625-5p | 1.5115 | 0.8471 | 2.6971 | 0.1620 | 74 |
| miR-675-5p | 14.575 | 0.0139 | *>*10000 | 0.4504 | 7 |
| miR-769-5p | 0.9375 | 0.6305 | 1.3938 | 0.7496 | 144 |
| miR-944 | 0.819 | 0.2697 | 2.4875 | 0.7247 | 35 |

**Differences in microRNAs**

In the following differences of miRNAs are considered. They are defined as miRNA1-miRNA2.

Unadjusted effects on differences

| **miRNA1** | **miRNA2** | **HR** | **HRlow** | **HRhigh** | **P** | **N** |
| --- | --- | --- | --- | --- | --- | --- |
| miR-148a | miR-212-3p | 1.2028 | 1.0911 | 1.3259 | 0.0002 | 155 |
| miR-205-5p | miR-769-5p | 0.9011 | 0.8520 | 0.9530 | 0.0003 | 129 |
| miR-148a | miR-205-5p | 1.0831 | 1.0360 | 1.1323 | 0.0004 | 130 |
| miR-34a-5p | miR-148a | 0.8328 | 0.7472 | 0.9281 | 0.0009 | 155 |
| miR-34a-5p | miR-187 | 0.6429 | 0.4950 | 0.8349 | 0.0009 | 47 |
| miR-146a-5p | miR-212-3p | 1.3245 | 1.1161 | 1.5718 | 0.0013 | 156 |
| miR-187 | miR-212-3p | 1.5503 | 1.1808 | 2.0354 | 0.0016 | 47 |
| miR-34a-5p | miR-769-5p | 0.7437 | 0.6181 | 0.8947 | 0.0017 | 154 |
| miR-212-3p | miR-769-5p | 0.8065 | 0.7038 | 0.9241 | 0.0020 | 154 |
| miR-205-5p | miR-625-5p | 0.9137 | 0.8621 | 0.9684 | 0.0023 | 72 |
| miR-205-5p | miR-450b-5p | 0.9118 | 0.8577 | 0.9694 | 0.0031 | 94 |
| miR-146a-5p | miR-205-5p | 1.0829 | 1.0269 | 1.1419 | 0.0033 | 130 |
| miR-205-5p | miR-222-5p | 0.9164 | 0.8645 | 0.9715 | 0.0034 | 127 |
| let-7g | miR-205-5p | 1.0786 | 1.0234 | 1.1369 | 0.0048 | 130 |
| miR-194-3p | miR-205-5p | 1.2588 | 1.0674 | 1.4845 | 0.0062 | 36 |
| miR-29a-5p | miR-205-5p | 1.0713 | 1.0188 | 1.1265 | 0.0072 | 129 |
| miR-125a-3p | miR-205-5p | 1.0835 | 1.0217 | 1.1490 | 0.0074 | 128 |
| let-7g | miR-187 | 0.8172 | 0.7018 | 0.9515 | 0.0093 | 47 |
| miR-34a-5p | miR-205-5p | 1.0733 | 1.0153 | 1.1346 | 0.0125 | 130 |
| miR-125a-3p | miR-148a | 0.8994 | 0.8266 | 0.9787 | 0.0139 | 152 |
| miR-125a-3p | miR-769-5p | 0.8372 | 0.7260 | 0.9655 | 0.0146 | 151 |
| miR-125a-3p | miR-187 | 0.7981 | 0.6649 | 0.9579 | 0.0155 | 46 |
| miR-212-3p | miR-625-5p | 0.8682 | 0.7712 | 0.9774 | 0.0194 | 79 |
| let-7g | miR-212-3p | 1.1246 | 1.0094 | 1.2530 | 0.0332 | 155 |
| miR-187 | miR-194-3p | 1.4147 | 1.0219 | 1.9586 | 0.0366 | 21 |
| miR-205-5p | miR-212-3p | 0.9456 | 0.8962 | 0.9977 | 0.0410 | 130 |
| miR-34a-5p | miR-625-5p | 0.8828 | 0.7819 | 0.9968 | 0.0443 | 79 |
| miR-146a-5p | miR-187 | 0.7925 | 0.6311 | 0.9951 | 0.0452 | 47 |
| miR-187 | miR-205-5p | 1.1244 | 1.0016 | 1.2621 | 0.0468 | 38 |
| miR-34a-5p | miR-146a-5p | 0.8254 | 0.6820 | 0.9989 | 0.0488 | 156 |
| miR-34a-5p | miR-212-3p | 1.1871 | 0.9884 | 1.4257 | 0.0665 | 156 |
| miR-125a-3p | miR-625-5p | 0.9015 | 0.8067 | 1.0074 | 0.0672 | 77 |
| miR-146a-5p | miR-148a | 0.9229 | 0.8465 | 1.0062 | 0.0688 | 156 |
| miR-187 | miR-450b-5p | 1.2944 | 0.9622 | 1.7412 | 0.0882 | 26 |
| miR-34a-5p | miR-194-3p | 0.8022 | 0.6211 | 1.0362 | 0.0914 | 49 |
| let-7g | miR-625-5p | 0.8978 | 0.7920 | 1.0178 | 0.0922 | 79 |
| miR-29a-5p | miR-769-5p | 0.8710 | 0.7409 | 1.0241 | 0.0946 | 154 |
| miR-625-5p | miR-944 | 1.1496 | 0.9758 | 1.3543 | 0.0954 | 22 |
| miR-148a | miR-187 | 0.8857 | 0.7657 | 1.0246 | 0.1024 | 47 |
| miR-29a-5p | miR-187 | 0.8585 | 0.7125 | 1.0343 | 0.1085 | 47 |
| miR-212-3p | miR-222-5p | 0.9028 | 0.7959 | 1.0241 | 0.1121 | 151 |
| miR-187 | miR-431-5p | 1.7920 | 0.8668 | 3.7046 | 0.1155 | 9 |
| miR-146a-5p | miR-769-5p | 0.8967 | 0.7781 | 1.0332 | 0.1315 | 155 |
| miR-148a | miR-450b-5p | 1.0816 | 0.9758 | 1.1990 | 0.1354 | 103 |
| let-7g | miR-34a-5p | 1.0985 | 0.9692 | 1.2452 | 0.1415 | 155 |
| miR-187 | miR-222-5p | 1.1513 | 0.9532 | 1.3905 | 0.1437 | 47 |
| miR-187 | miR-769-5p | 1.1671 | 0.9476 | 1.4374 | 0.1460 | 47 |
| miR-450b-5p | miR-769-5p | 0.8683 | 0.7164 | 1.0523 | 0.1498 | 103 |
| miR-148a | miR-431-5p | 1.1435 | 0.9507 | 1.3754 | 0.1547 | 47 |
| miR-125a-3p | miR-431-5p | 1.1888 | 0.9340 | 1.5130 | 0.1600 | 47 |
| miR-222-5p | miR-769-5p | 0.9075 | 0.7920 | 1.0398 | 0.1622 | 152 |
| miR-194-3p | miR-769-5p | 1.2302 | 0.9174 | 1.6495 | 0.1663 | 50 |
| miR-222-5p | miR-625-5p | 0.9151 | 0.8070 | 1.0376 | 0.1664 | 78 |
| miR-29a-5p | miR-194-3p | 0.8475 | 0.6700 | 1.0720 | 0.1675 | 50 |
| miR-29a-5p | miR-212-3p | 1.0735 | 0.9695 | 1.1886 | 0.1726 | 155 |
| let-7g | miR-769-5p | 0.9328 | 0.8427 | 1.0325 | 0.1794 | 154 |
| miR-194-3p | miR-212-3p | 1.1445 | 0.9385 | 1.3956 | 0.1825 | 49 |
| let-7g | miR-148a | 0.9472 | 0.8721 | 1.0288 | 0.1981 | 156 |
| miR-125a-3p | miR-212-3p | 1.0854 | 0.9566 | 1.2315 | 0.2034 | 153 |
| miR-125a-3p | miR-146a-5p | 0.9138 | 0.7942 | 1.0514 | 0.2078 | 153 |
| miR-194-3p | miR-222-5p | 1.1471 | 0.9214 | 1.4281 | 0.2197 | 50 |
| miR-29a-5p | miR-625-5p | 0.9302 | 0.8286 | 1.0442 | 0.2198 | 79 |
| miR-125a-3p | miR-194-3p | 0.8904 | 0.7369 | 1.0758 | 0.2290 | 47 |
| miR-146a-5p | miR-450b-5p | 1.1109 | 0.9352 | 1.3196 | 0.2312 | 103 |
| let-7g | miR-125a-3p | 1.0709 | 0.9553 | 1.2006 | 0.2399 | 152 |
| miR-29a-5p | miR-148a | 0.9683 | 0.9177 | 1.0218 | 0.2403 | 155 |
| miR-194-3p | miR-625-5p | 1.3402 | 0.8122 | 2.2113 | 0.2518 | 25 |
| miR-625-5p | miR-769-5p | 1.0769 | 0.9483 | 1.2230 | 0.2537 | 79 |
| miR-146a-5p | miR-431-5p | 1.1399 | 0.9062 | 1.4339 | 0.2632 | 47 |
| miR-148a | miR-675-5p | 1.2793 | 0.8286 | 1.9752 | 0.2663 | 8 |
| miR-212-3p | miR-450b-5p | 0.9233 | 0.8002 | 1.0652 | 0.2739 | 103 |
| miR-205-5p | miR-675-5p | 0.8262 | 0.5848 | 1.1671 | 0.2786 | 6 |
| let-7g | miR-944 | 1.0858 | 0.9332 | 1.2634 | 0.2868 | 38 |
| miR-431-5p | miR-769-5p | 0.8864 | 0.7077 | 1.1102 | 0.2938 | 47 |
| miR-222-5p | miR-944 | 1.0974 | 0.9147 | 1.3167 | 0.3172 | 38 |
| miR-29a-5p | miR-431-5p | 1.1145 | 0.8960 | 1.3864 | 0.3303 | 47 |
| miR-194-3p | miR-450b-5p | 1.1266 | 0.8845 | 1.4350 | 0.3342 | 32 |
| miR-29a-5p | miR-450b-5p | 1.0842 | 0.9157 | 1.2837 | 0.3483 | 103 |
| miR-187 | miR-944 | 1.2847 | 0.7526 | 2.1931 | 0.3585 | 8 |
| miR-148a | miR-222-5p | 1.0262 | 0.9669 | 1.0892 | 0.3944 | 152 |
| miR-675-5p | miR-769-5p | 0.7471 | 0.3794 | 1.4711 | 0.3991 | 8 |
| miR-125a-3p | miR-675-5p | 1.4604 | 0.6031 | 3.5365 | 0.4013 | 8 |
| miR-431-5p | miR-625-5p | 0.9141 | 0.7410 | 1.1277 | 0.4020 | 27 |
| miR-146a-5p | miR-194-3p | 0.9336 | 0.7948 | 1.0967 | 0.4032 | 50 |
| miR-187 | miR-625-5p | 1.0897 | 0.8840 | 1.3433 | 0.4209 | 28 |
| miR-125a-3p | miR-450b-5p | 0.9475 | 0.8277 | 1.0846 | 0.4338 | 103 |
| miR-194-3p | miR-431-5p | 1.2164 | 0.7400 | 1.9995 | 0.4398 | 10 |
| miR-212-3p | miR-944 | 0.9413 | 0.8025 | 1.1040 | 0.4571 | 38 |
| miR-222-5p | miR-675-5p | 0.6246 | 0.1802 | 2.1643 | 0.4579 | 8 |
| miR-146a-5p | miR-625-5p | 0.9503 | 0.8297 | 1.0884 | 0.4611 | 79 |
| miR-205-5p | miR-944 | 1.0439 | 0.9294 | 1.1726 | 0.4683 | 32 |
| let-7g | miR-675-5p | 1.1274 | 0.8139 | 1.5617 | 0.4707 | 8 |
| let-7g | miR-194-3p | 0.9399 | 0.7932 | 1.1138 | 0.4741 | 50 |
| miR-205-5p | miR-431-5p | 0.9719 | 0.8981 | 1.0516 | 0.4782 | 43 |
| miR-34a-5p | miR-450b-5p | 0.9387 | 0.7870 | 1.1195 | 0.4815 | 103 |
| miR-146a-5p | miR-675-5p | 1.4607 | 0.5039 | 4.2345 | 0.4853 | 8 |
| let-7g | miR-146a-5p | 1.0359 | 0.9373 | 1.1448 | 0.4895 | 156 |
| miR-34a-5p | miR-222-5p | 0.9552 | 0.8389 | 1.0877 | 0.4896 | 151 |
| miR-222-5p | miR-431-5p | 1.0699 | 0.8704 | 1.3152 | 0.5211 | 47 |
| miR-148a | miR-944 | 0.9454 | 0.7963 | 1.1223 | 0.5212 | 38 |
| let-7g | miR-431-5p | 1.0529 | 0.8940 | 1.2401 | 0.5365 | 47 |
| miR-450b-5p | miR-625-5p | 0.9547 | 0.8228 | 1.1078 | 0.5411 | 60 |
| miR-125a-3p | miR-222-5p | 0.9653 | 0.8582 | 1.0857 | 0.5557 | 148 |
| miR-450b-5p | miR-944 | 1.0503 | 0.8813 | 1.2517 | 0.5834 | 31 |
| let-7g | miR-29a-5p | 1.0187 | 0.9400 | 1.1039 | 0.6522 | 155 |
| miR-769-5p | miR-944 | 1.0458 | 0.8601 | 1.2716 | 0.6536 | 38 |
| miR-34a-5p | miR-431-5p | 1.0572 | 0.8197 | 1.3636 | 0.6683 | 47 |
| miR-450b-5p | miR-675-5p | 0.6587 | 0.0877 | 4.9490 | 0.6849 | 6 |
| miR-212-3p | miR-675-5p | 1.1788 | 0.5281 | 2.6313 | 0.6881 | 8 |
| miR-146a-5p | miR-222-5p | 0.9810 | 0.8900 | 1.0813 | 0.6993 | 152 |
| miR-29a-5p | miR-944 | 1.0387 | 0.8543 | 1.2629 | 0.7032 | 38 |
| miR-29a-5p | miR-222-5p | 1.0227 | 0.9026 | 1.1588 | 0.7243 | 151 |
| miR-29a-5p | miR-125a-3p | 1.0171 | 0.9220 | 1.1220 | 0.7352 | 152 |
| miR-29a-5p | miR-34a-5p | 1.0201 | 0.9089 | 1.1449 | 0.7359 | 155 |
| miR-148a | miR-194-3p | 0.9807 | 0.8739 | 1.1006 | 0.7410 | 50 |
| miR-431-5p | miR-450b-5p | 0.9612 | 0.7430 | 1.2435 | 0.7633 | 37 |
| miR-29a-5p | miR-146a-5p | 1.0124 | 0.9305 | 1.1015 | 0.7748 | 156 |
| miR-148a | miR-625-5p | 1.0144 | 0.9061 | 1.1355 | 0.8044 | 79 |
| let-7g | miR-222-5p | 0.9882 | 0.8980 | 1.0875 | 0.8083 | 152 |
| miR-34a-5p | miR-944 | 0.9787 | 0.8181 | 1.1708 | 0.8137 | 38 |
| miR-125a-3p | miR-944 | 1.0226 | 0.8460 | 1.2359 | 0.8176 | 38 |
| miR-34a-5p | miR-675-5p | 1.0647 | 0.5509 | 2.0578 | 0.8520 | 8 |
| miR-194-3p | miR-944 | 0.9700 | 0.6822 | 1.3792 | 0.8652 | 10 |
| miR-148a | miR-769-5p | 1.0052 | 0.9425 | 1.0721 | 0.8749 | 154 |
| miR-34a-5p | miR-125a-3p | 0.9915 | 0.8575 | 1.1465 | 0.9084 | 153 |
| miR-212-3p | miR-431-5p | 0.9881 | 0.7719 | 1.2649 | 0.9244 | 47 |
| miR-431-5p | miR-944 | 0.9914 | 0.7969 | 1.2333 | 0.9379 | 19 |
| miR-222-5p | miR-450b-5p | 0.9972 | 0.8645 | 1.1501 | 0.9689 | 102 |
| miR-29a-5p | miR-675-5p | 0.9897 | 0.2916 | 3.3595 | 0.9868 | 8 |
| miR-146a-5p | miR-944 | 1.0011 | 0.8331 | 1.2029 | 0.9908 | 38 |
| let-7g | miR-450b-5p | 0.9999 | 0.8800 | 1.1361 | 0.9987 | 103 |
| miR-187 | miR-675-5p |  |  |  |  | 1 |
| miR-194-3p | miR-675-5p |  |  |  |  | 2 |
| miR-431-5p | miR-675-5p |  |  |  |  | 5 |
| miR-625-5p | miR-675-5p |  |  |  |  | 2 |
| miR-675-5p | miR-944 |  |  |  |  | 3 |

Adjusted (age, sex, tumor stage, ASA-score, tumor differentiation) effects on differences

| **miRNA1** | **miRNA2** | **HR** | **HRlow** | **HRhigh** | **P** | **N** |
| --- | --- | --- | --- | --- | --- | --- |
| miR-34a-5p | miR-148a | 0.8184 | 0.7255 | 0.9231 | 0.0011 | 144 |
| miR-205-5p | miR-769-5p | 0.9055 | 0.8517 | 0.9626 | 0.0015 | 119 |
| miR-146a-5p | miR-212-3p | 1.3335 | 1.1136 | 1.5968 | 0.0017 | 145 |
| miR-34a-5p | miR-187 | 0.6747 | 0.5168 | 0.8809 | 0.0038 | 44 |
| miR-148a | miR-205-5p | 1.0722 | 1.0224 | 1.1243 | 0.004 | 120 |
| miR-29a-5p | miR-205-5p | 1.0845 | 1.0254 | 1.1472 | 0.0046 | 119 |
| miR-125a-3p | miR-769-5p | 0.8069 | 0.6901 | 0.9434 | 0.0071 | 140 |
| miR-187 | miR-212-3p | 1.4722 | 1.1072 | 1.9574 | 0.0078 | 44 |
| let-7g | miR-187 | 0.7374 | 0.5876 | 0.9253 | 0.0085 | 44 |
| miR-146a-5p | miR-205-5p | 1.0779 | 1.0183 | 1.141 | 0.0097 | 120 |
| miR-205-5p | miR-222-5p | 0.9258 | 0.8699 | 0.9852 | 0.0152 | 117 |
| miR-29a-5p | miR-769-5p | 0.8072 | 0.6769 | 0.9625 | 0.0171 | 143 |
| let-7g | miR-205-5p | 1.0679 | 1.0113 | 1.1275 | 0.018 | 120 |
| miR-29a-5p | miR-194-3p | 0.6832 | 0.4971 | 0.9388 | 0.0188 | 46 |
| miR-125a-3p | miR-187 | 0.7629 | 0.6087 | 0.9562 | 0.0188 | 43 |
| let-7g | miR-212-3p | 1.1434 | 1.0184 | 1.2838 | 0.0233 | 144 |
| miR-125a-3p | miR-205-5p | 1.0746 | 1.0097 | 1.1436 | 0.0236 | 118 |
| miR-205-5p | miR-450b-5p | 0.9255 | 0.8654 | 0.9899 | 0.024 | 85 |
| miR-34a-5p | miR-194-3p | 0.6395 | 0.4313 | 0.9484 | 0.0262 | 45 |
| miR-194-3p | miR-212-3p | 1.385 | 1.0372 | 1.8495 | 0.0273 | 45 |
| miR-212-3p | miR-625-5p | 0.8566 | 0.745 | 0.9849 | 0.0298 | 74 |
| miR-34a-5p | miR-205-5p | 1.0669 | 1.006 | 1.1315 | 0.0307 | 120 |
| miR-194-3p | miR-205-5p | 1.2154 | 1.0163 | 1.4535 | 0.0326 | 33 |
| miR-625-5p | miR-944 | 1.5138 | 1.0321 | 2.2203 | 0.0339 | 20 |
| miR-125a-3p | miR-148a | 0.9117 | 0.8354 | 0.995 | 0.0383 | 141 |
| miR-146a-5p | miR-769-5p | 0.8381 | 0.7085 | 0.9915 | 0.0394 | 144 |
| miR-34a-5p | miR-625-5p | 0.8668 | 0.7524 | 0.9986 | 0.0478 | 74 |
| miR-205-5p | miR-625-5p | 0.9376 | 0.8785 | 1.0006 | 0.0522 | 67 |
| miR-34a-5p | miR-212-3p | 1.2156 | 0.997 | 1.4821 | 0.0536 | 145 |
| miR-187 | miR-450b-5p | 3.7805 | 0.968 | 14.7641 | 0.0557 | 23 |
| miR-146a-5p | miR-187 | 0.7898 | 0.6188 | 1.008 | 0.058 | 44 |
| miR-222-5p | miR-769-5p | 0.8763 | 0.7627 | 1.0068 | 0.0623 | 141 |
| miR-29a-5p | miR-187 | 0.805 | 0.6405 | 1.0116 | 0.0628 | 44 |
| miR-450b-5p | miR-769-5p | 0.8071 | 0.644 | 1.0115 | 0.0628 | 93 |
| miR-187 | miR-625-5p | 0.752 | 0.5489 | 1.0302 | 0.076 | 28 |
| miR-146a-5p | miR-148a | 0.9197 | 0.838 | 1.0093 | 0.0774 | 145 |
| miR-148a | miR-431-5p | 1.2434 | 0.9744 | 1.5866 | 0.0799 | 42 |
| miR-148a | miR-187 | 0.8399 | 0.6905 | 1.0216 | 0.0808 | 44 |
| miR-148a | miR-450b-5p | 1.1108 | 0.9864 | 1.2508 | 0.0828 | 93 |
| miR-205-5p | miR-212-3p | 0.9517 | 0.8992 | 1.0074 | 0.0878 | 120 |
| miR-34a-5p | miR-146a-5p | 0.8452 | 0.6939 | 1.0295 | 0.0947 | 145 |
| miR-125a-3p | miR-212-3p | 1.12 | 0.9789 | 1.2815 | 0.0989 | 142 |
| miR-194-3p | miR-944 | 0.2202 | 0.0345 | 1.405 | 0.1095 | 10 |
| let-7g | miR-769-5p | 0.9191 | 0.8282 | 1.0201 | 0.1127 | 143 |
| let-7g | miR-34a-5p | 1.117 | 0.9742 | 1.2808 | 0.113 | 144 |
| miR-29a-5p | miR-212-3p | 1.0902 | 0.9755 | 1.2184 | 0.1277 | 144 |
| miR-125a-3p | miR-625-5p | 0.9159 | 0.8115 | 1.0338 | 0.155 | 72 |
| miR-194-3p | miR-769-5p | 1.3171 | 0.8952 | 1.9378 | 0.1621 | 46 |
| miR-212-3p | miR-222-5p | 0.9131 | 0.8027 | 1.0388 | 0.1671 | 140 |
| miR-125a-3p | miR-431-5p | 1.3132 | 0.8914 | 1.9346 | 0.168 | 42 |
| miR-450b-5p | miR-944 | 1.2535 | 0.9053 | 1.7357 | 0.1736 | 28 |
| miR-187 | miR-194-3p | 1.4515 | 0.8423 | 2.5012 | 0.1796 | 20 |
| miR-146a-5p | miR-450b-5p | 1.1536 | 0.9325 | 1.4272 | 0.1882 | 93 |
| miR-194-3p | miR-222-5p | 1.1665 | 0.9232 | 1.4738 | 0.1969 | 46 |
| miR-222-5p | miR-944 | 1.1863 | 0.9151 | 1.5377 | 0.197 | 35 |
| miR-212-3p | miR-450b-5p | 0.8918 | 0.7479 | 1.0635 | 0.2024 | 93 |
| miR-431-5p | miR-625-5p | 0.8358 | 0.632 | 1.1054 | 0.2085 | 24 |
| miR-29a-5p | miR-625-5p | 0.9173 | 0.8017 | 1.0496 | 0.2091 | 74 |
| miR-187 | miR-769-5p | 1.191 | 0.904 | 1.569 | 0.2141 | 44 |
| miR-222-5p | miR-431-5p | 1.2185 | 0.8916 | 1.6652 | 0.215 | 42 |
| miR-29a-5p | miR-450b-5p | 1.1243 | 0.9212 | 1.3721 | 0.249 | 93 |
| miR-34a-5p | miR-675-5p | 3.5062 | 0.4153 | 29.5985 | 0.249 | 7 |
| miR-146a-5p | miR-431-5p | 1.2099 | 0.8747 | 1.6737 | 0.2497 | 42 |
| miR-187 | miR-222-5p | 1.1372 | 0.9132 | 1.4162 | 0.2507 | 44 |
| miR-125a-3p | miR-194-3p | 0.8611 | 0.6669 | 1.112 | 0.2517 | 43 |
| miR-146a-5p | miR-675-5p | 3.037 | 0.4389 | 21.0133 | 0.2603 | 7 |
| miR-625-5p | miR-769-5p | 1.0819 | 0.9422 | 1.2423 | 0.2644 | 74 |
| miR-148a | miR-675-5p | 3.1888 | 0.412 | 24.6815 | 0.2667 | 7 |
| let-7g | miR-125a-3p | 1.0736 | 0.9468 | 1.2175 | 0.2681 | 141 |
| let-7g | miR-944 | 1.1077 | 0.9138 | 1.3429 | 0.2975 | 35 |
| miR-146a-5p | miR-194-3p | 0.8824 | 0.6958 | 1.119 | 0.3019 | 46 |
| let-7g | miR-148a | 0.9551 | 0.8741 | 1.0437 | 0.31 | 145 |
| miR-431-5p | miR-769-5p | 0.8416 | 0.603 | 1.1745 | 0.3104 | 42 |
| miR-29a-5p | miR-148a | 0.9719 | 0.9166 | 1.0306 | 0.3405 | 144 |
| miR-212-3p | miR-675-5p | 2.16 | 0.4432 | 10.5261 | 0.3406 | 7 |
| miR-194-3p | miR-625-5p | 0.6395 | 0.2514 | 1.6272 | 0.3482 | 24 |
| let-7g | miR-146a-5p | 1.0518 | 0.9432 | 1.1728 | 0.3638 | 145 |
| let-7g | miR-625-5p | 0.935 | 0.8057 | 1.0849 | 0.3756 | 74 |
| miR-125a-3p | miR-146a-5p | 0.9344 | 0.7987 | 1.0933 | 0.3972 | 142 |
| miR-769-5p | miR-944 | 1.1262 | 0.8547 | 1.484 | 0.3983 | 35 |
| miR-148a | miR-625-5p | 1.0678 | 0.9168 | 1.2435 | 0.3992 | 74 |
| miR-222-5p | miR-625-5p | 0.9397 | 0.8129 | 1.0862 | 0.4 | 73 |
| miR-34a-5p | miR-450b-5p | 0.911 | 0.7332 | 1.1319 | 0.4002 | 93 |
| miR-29a-5p | miR-431-5p | 1.1312 | 0.847 | 1.5108 | 0.4036 | 42 |
| miR-29a-5p | miR-944 | 1.1122 | 0.8587 | 1.4406 | 0.4204 | 35 |
| miR-146a-5p | miR-625-5p | 0.9418 | 0.8114 | 1.093 | 0.4298 | 74 |
| miR-148a | miR-222-5p | 1.0237 | 0.963 | 1.0883 | 0.4526 | 141 |
| miR-125a-3p | miR-944 | 1.0838 | 0.8533 | 1.3766 | 0.5095 | 35 |
| miR-34a-5p | miR-431-5p | 1.1243 | 0.765 | 1.6523 | 0.5509 | 42 |
| miR-29a-5p | miR-675-5p | 1.5976 | 0.3239 | 7.8796 | 0.565 | 7 |
| miR-146a-5p | miR-944 | 1.0752 | 0.8353 | 1.3839 | 0.5734 | 35 |
| let-7g | miR-431-5p | 1.064 | 0.8405 | 1.347 | 0.606 | 42 |
| miR-146a-5p | miR-222-5p | 0.9739 | 0.8807 | 1.077 | 0.6068 | 141 |
| miR-34a-5p | miR-222-5p | 0.9702 | 0.8516 | 1.1053 | 0.6491 | 140 |
| miR-29a-5p | miR-146a-5p | 1.021 | 0.9306 | 1.1203 | 0.6601 | 145 |
| let-7g | miR-29a-5p | 1.0192 | 0.9351 | 1.1109 | 0.6655 | 144 |
| miR-222-5p | miR-675-5p | 1.976 | 0.0866 | 45.0866 | 0.6695 | 7 |
| miR-34a-5p | miR-125a-3p | 0.9676 | 0.8315 | 1.1259 | 0.6698 | 142 |
| miR-29a-5p | miR-34a-5p | 1.027 | 0.9058 | 1.1643 | 0.6779 | 144 |
| miR-431-5p | miR-450b-5p | 0.9409 | 0.6961 | 1.2718 | 0.6918 | 32 |
| miR-29a-5p | miR-222-5p | 1.0221 | 0.8992 | 1.1617 | 0.7382 | 140 |
| let-7g | miR-675-5p | *>*10000 | *<*0.0001 | *>*10000 | 0.7425 | 7 |
| miR-450b-5p | miR-625-5p | 0.9698 | 0.7992 | 1.1769 | 0.7564 | 55 |
| miR-212-3p | miR-431-5p | 1.0463 | 0.7551 | 1.4498 | 0.7859 | 42 |
| let-7g | miR-450b-5p | 1.0202 | 0.8826 | 1.1793 | 0.7865 | 93 |
| let-7g | miR-194-3p | 0.9702 | 0.7779 | 1.2101 | 0.7886 | 46 |
| miR-205-5p | miR-431-5p | 0.9871 | 0.8901 | 1.0948 | 0.8064 | 38 |
| miR-29a-5p | miR-125a-3p | 1.014 | 0.9059 | 1.135 | 0.8085 | 141 |
| miR-212-3p | miR-944 | 0.9738 | 0.7799 | 1.2158 | 0.8144 | 35 |
| miR-148a | miR-944 | 1.0236 | 0.7895 | 1.3271 | 0.8603 | 35 |
| miR-148a | miR-769-5p | 0.9941 | 0.9285 | 1.0644 | 0.866 | 143 |
| miR-125a-3p | miR-222-5p | 0.9895 | 0.869 | 1.1266 | 0.8729 | 137 |
| miR-431-5p | miR-944 | 0.9686 | 0.6311 | 1.4865 | 0.8839 | 17 |
| let-7g | miR-222-5p | 0.9944 | 0.9011 | 1.0973 | 0.9107 | 141 |
| miR-222-5p | miR-450b-5p | 0.9915 | 0.8431 | 1.166 | 0.9175 | 92 |
| miR-187 | miR-944 | *<*0.0001 | *<*0.0001 | *>*10000 | 0.9309 | 7 |
| miR-34a-5p | miR-944 | 1.0116 | 0.7727 | 1.3245 | 0.9329 | 35 |
| miR-125a-3p | miR-450b-5p | 0.9931 | 0.8349 | 1.1813 | 0.9378 | 93 |
| miR-205-5p | miR-944 | 1.0039 | 0.8609 | 1.1707 | 0.9601 | 29 |
| miR-194-3p | miR-450b-5p | 0.9931 | 0.7042 | 1.4006 | 0.9685 | 29 |
| miR-187 | miR-205-5p | 1.0006 | 0.8665 | 1.1554 | 0.9939 | 36 |
| miR-125a-3p | miR-675-5p | *>*10000 | *<*0.0001 | *>*10000 | 0.9981 | 7 |
| miR-194-3p | miR-431-5p | *<*0.0001 | *<*0.0001 | *>*10000 | 0.9984 | 10 |
| miR-675-5p | miR-769-5p | *<*0.0001 | *<*0.0001 | *>*10000 | 0.9984 | 7 |
| miR-148a | miR-194-3p | 0.9999 | 0.8703 | 1.1488 | 0.999 | 46 |
| miR-205-5p | miR-675-5p | 0.4025 | *<*0.0001 | *>*10000 | 0.9999 | 5 |
| miR-450b-5p | miR-675-5p | 1319.5262 | *<*0.0001 | *>*10000 | 0.9999 | 5 |
| miR-187 | miR-431-5p | 10.0977 | *<*0.0001 | *>*10000 | 1 | 8 |
| miR-34a-5p | miR-769-5p | 0.6838 | 0.5588 | 0.8368 | 2e-04 | 143 |
| miR-148a | miR-212-3p | 1.2097 | 1.0906 | 1.3416 | 3e-04 | 144 |
| miR-212-3p | miR-769-5p | 0.7723 | 0.668 | 0.8928 | 5e-04 | 143 |
| miR-187 | miR-675-5p |  |  |  |  | 1 |
| miR-194-3p | miR-675-5p |  |  |  |  | 2 |
| miR-431-5p | miR-675-5p |  |  |  |  | 5 |
| miR-625-5p | miR-675-5p |  |  |  |  | 2 |
| miR-675-5p | miR-944 |  |  |  |  | 3 |

**Prognosis on diagnosis: PDAC**

In the following miRNAs univariately are considered.

Unadjusted univariate effects

| **miRNA** | **HR** | **HRlow** | **HRhigh** | **P** | **N** |
| --- | --- | --- | --- | --- | --- |
| let-7g | 1.0185 | 0.8790 | 1.1802 | 0.8072 | 103 |
| miR-29a-5p | 0.9111 | 0.7467 | 1.1119 | 0.3597 | 102 |
| miR-34a-5p | 0.7189 | 0.5585 | 0.9255 | 0.0104 | 103 |
| miR-125a-3p | 0.9026 | 0.7546 | 1.0795 | 0.2616 | 102 |
| miR-146a-5p | 0.9129 | 0.7455 | 1.1179 | 0.3779 | 103 |
| miR-148a | 1.0726 | 0.9543 | 1.2055 | 0.2401 | 103 |
| miR-187 | 1.0743 | 0.7992 | 1.4440 | 0.6349 | 23 |
| miR-194-3p | 1.2700 | 0.8520 | 1.8930 | 0.2406 | 25 |
| miR-205-5p | 0.9516 | 0.8961 | 1.0107 | 0.1064 | 93 |
| miR-212-3p | 0.8347 | 0.7071 | 0.9853 | 0.0328 | 103 |
| miR-222-5p | 0.8887 | 0.7534 | 1.0483 | 0.1616 | 102 |
| miR-431-5p | 0.9369 | 0.6933 | 1.2662 | 0.6716 | 39 |
| miR-450b-5p | 0.9213 | 0.7510 | 1.1302 | 0.4316 | 76 |
| miR-625-5p | 1.0253 | 0.8801 | 1.1945 | 0.7482 | 48 |
| miR-675-5p | 0.7535 | 0.2946 | 1.9269 | 0.5546 | 7 |
| miR-769-5p | 1.0129 | 0.7674 | 1.3369 | 0.9280 | 102 |
| miR-944 | 0.9912 | 0.7925 | 1.2397 | 0.9381 | 32 |

Adjusted (age, sex, tumor stage, ASA-score, tumor differentiation) univariate effects

| **miRNA** | **HR** | **HRlow** | **HRhigh** | **P** | **N** |
| --- | --- | --- | --- | --- | --- |
| let-7g | 1.0024 | 0.851 | 1.1806 | 0.9772 | 93 |
| miR-29a-5p | 0.9201 | 0.7357 | 1.1507 | 0.4654 | 92 |
| miR-34a-5p | 0.6962 | 0.521 | 0.9304 | 0.0144 | 93 |
| miR-125a-3p | 0.9233 | 0.7561 | 1.1275 | 0.4338 | 92 |
| miR-146a-5p | 0.9166 | 0.7312 | 1.149 | 0.4499 | 93 |
| miR-148a | 1.0632 | 0.9338 | 1.2104 | 0.3547 | 93 |
| miR-187 | 0.715 | 0.4462 | 1.1457 | 0.1631 | 20 |
| miR-194-3p | 1.5442 | 0.8128 | 2.934 | 0.1845 | 22 |
| miR-205-5p | 0.9541 | 0.8917 | 1.0208 | 0.1731 | 84 |
| miR-212-3p | 0.8217 | 0.6846 | 0.9863 | 0.0350 | 93 |
| miR-222-5p | 0.8897 | 0.7511 | 1.0539 | 0.1763 | 92 |
| miR-431-5p | 0.9041 | 0.6152 | 1.3286 | 0.6077 | 34 |
| miR-450b-5p | 0.9375 | 0.726 | 1.2106 | 0.6206 | 67 |
| miR-625-5p | 1.0392 | 0.8566 | 1.2608 | 0.6964 | 44 |
| miR-675-5p | *<*0.0001 | *<*0.0001 | *>*10000 | 0.9996 | 6 |
| miR-769-5p | 1.1664 | 0.8674 | 1.5685 | 0.3084 | 92 |
| miR-944 | 0.8633 | 0.6395 | 1.1653 | 0.3367 | 29 |

Unadjusted univariate effects (under median vs. over median)

| **miRNA** | **HR** | **HRlow** | **HRhigh** | **P** | **N** |
| --- | --- | --- | --- | --- | --- |
| let-7g | 1.1249 | 0.7287 | 1.7363 | 0.5953 | 103 |
| miR-29a-5p | 0.6966 | 0.4491 | 1.0806 | 0.1065 | 102 |
| miR-34a-5p | 0.4879 | 0.3095 | 0.7694 | 0.0020 | 103 |
| miR-125a-3p | 0.9174 | 0.5898 | 1.4270 | 0.7022 | 102 |
| miR-146a-5p | 0.8512 | 0.5511 | 1.3146 | 0.4675 | 103 |
| miR-148a | 1.4783 | 0.9537 | 2.2914 | 0.0804 | 103 |
| miR-187 | 1.5642 | 0.5990 | 4.0848 | 0.3610 | 23 |
| miR-194-3p | 1.6294 | 0.5790 | 4.5855 | 0.3551 | 25 |
| miR-205-5p | 0.6506 | 0.4075 | 1.0385 | 0.0716 | 93 |
| miR-212-3p | 0.6357 | 0.4110 | 0.9832 | 0.0417 | 103 |
| miR-222-5p | 0.7200 | 0.4651 | 1.1146 | 0.1407 | 102 |
| miR-431-5p | 1.0679 | 0.5329 | 2.1402 | 0.8530 | 39 |
| miR-450b-5p | 0.7937 | 0.4773 | 1.3198 | 0.3733 | 76 |
| miR-625-5p | 1.2469 | 0.6555 | 2.3718 | 0.5013 | 48 |
| miR-675-5p | 1.3270 | 0.2111 | 8.3407 | 0.7629 | 7 |
| miR-769-5p | 0.9529 | 0.6156 | 1.4750 | 0.8287 | 102 |
| miR-944 | 1.2742 | 0.5964 | 2.7221 | 0.5316 | 32 |

Adjusted (age, sex, tumor stage, ASA-score, tumor differentiation) univariate effects (under median vs. over median)

| **miRNA** | **HR** | **HRlow** | **HRhigh** | **P** | **N** |
| --- | --- | --- | --- | --- | --- |
| let-7g | 1.0064 | 0.6016 | 1.6839 | 0.9805 | 93 |
| miR-29a-5p | 0.7439 | 0.4545 | 1.2177 | 0.2393 | 92 |
| miR-34a-5p | 0.5348 | 0.3228 | 0.886 | 0.0151 | 93 |
| miR-125a-3p | 0.9178 | 0.5526 | 1.5242 | 0.7402 | 92 |
| miR-146a-5p | 0.8518 | 0.5232 | 1.3866 | 0.5187 | 93 |
| miR-148a | 1.5225 | 0.9351 | 2.4789 | 0.0910 | 93 |
| miR-187 | 0.2163 | 0.0466 | 1.0041 | 0.0506 | 20 |
| miR-194-3p | 2.7288 | 0.6014 | 12.3816 | 0.1933 | 22 |
| miR-205-5p | 0.738 | 0.4297 | 1.2675 | 0.2709 | 84 |
| miR-212-3p | 0.5933 | 0.3645 | 0.9659 | 0.0358 | 93 |
| miR-222-5p | 0.7156 | 0.4348 | 1.1776 | 0.1879 | 92 |
| miR-431-5p | 0.8229 | 0.2885 | 2.3467 | 0.7154 | 34 |
| miR-450b-5p | 0.7798 | 0.4277 | 1.4217 | 0.4169 | 67 |
| miR-625-5p | 1.1909 | 0.5418 | 2.6181 | 0.6637 | 44 |
| miR-675-5p | *<*0.0001 | *<*0.0001 | *>*10000 | 0.9996 | 6 |
| miR-769-5p | 1.2256 | 0.7596 | 1.9776 | 0.4046 | 92 |
| miR-944 | 0.8314 | 0.2675 | 2.584 | 0.7497 | 29 |

**Differences in microRNAs**

Unadjusted effects on differences

| **miRNA1** | **miRNA2** | **HR** | **HRlow** | **HRhigh** | **P** | **N** |
| --- | --- | --- | --- | --- | --- | --- |
| miR-148a | miR-212-3p | 1.1765 | 1.0440 | 1.3258 | 0.0077 | 103 |
| miR-34a-5p | miR-148a | 0.8617 | 0.7638 | 0.9721 | 0.0156 | 103 |
| miR-34a-5p | miR-769-5p | 0.7480 | 0.5859 | 0.9551 | 0.0199 | 102 |
| miR-146a-5p | miR-212-3p | 1.2578 | 1.0138 | 1.5605 | 0.0371 | 103 |
| miR-34a-5p | miR-146a-5p | 0.7446 | 0.5598 | 0.9904 | 0.0427 | 103 |
| miR-212-3p | miR-769-5p | 0.8343 | 0.6914 | 1.0068 | 0.0589 | 102 |
| let-7g | miR-212-3p | 1.1315 | 0.9937 | 1.2885 | 0.0622 | 103 |
| miR-148a | miR-205-5p | 1.0493 | 0.9974 | 1.1039 | 0.0630 | 93 |
| miR-148a | miR-222-5p | 1.0855 | 0.9917 | 1.1883 | 0.0753 | 102 |
| miR-205-5p | miR-769-5p | 0.9445 | 0.8866 | 1.0062 | 0.0768 | 93 |
| let-7g | miR-34a-5p | 1.1264 | 0.9814 | 1.2929 | 0.0904 | 103 |
| miR-205-5p | miR-450b-5p | 0.9446 | 0.8842 | 1.0092 | 0.0914 | 72 |
| miR-29a-5p | miR-212-3p | 1.1403 | 0.9788 | 1.3284 | 0.0919 | 102 |
| miR-194-3p | miR-205-5p | 1.1928 | 0.9706 | 1.4658 | 0.0936 | 21 |
| miR-29a-5p | miR-205-5p | 1.0541 | 0.9907 | 1.1216 | 0.0957 | 92 |
| miR-125a-3p | miR-431-5p | 1.2447 | 0.9528 | 1.6260 | 0.1083 | 39 |
| miR-148a | miR-431-5p | 1.1620 | 0.9663 | 1.3972 | 0.1106 | 39 |
| miR-125a-3p | miR-148a | 0.9205 | 0.8314 | 1.0192 | 0.1111 | 102 |
| miR-450b-5p | miR-944 | 1.1641 | 0.9626 | 1.4078 | 0.1171 | 27 |
| miR-125a-3p | miR-205-5p | 1.0510 | 0.9852 | 1.1213 | 0.1317 | 92 |
| miR-222-5p | miR-769-5p | 0.8833 | 0.7486 | 1.0421 | 0.1413 | 102 |
| miR-146a-5p | miR-205-5p | 1.0458 | 0.9845 | 1.1110 | 0.1463 | 93 |
| miR-431-5p | miR-625-5p | 0.8597 | 0.7003 | 1.0553 | 0.1484 | 24 |
| miR-29a-5p | miR-34a-5p | 1.1275 | 0.9568 | 1.3288 | 0.1520 | 102 |
| miR-146a-5p | miR-148a | 0.9291 | 0.8391 | 1.0287 | 0.1567 | 103 |
| let-7g | miR-205-5p | 1.0418 | 0.9832 | 1.1040 | 0.1659 | 93 |
| miR-29a-5p | miR-148a | 0.9358 | 0.8503 | 1.0299 | 0.1745 | 102 |
| miR-212-3p | miR-625-5p | 0.9007 | 0.7738 | 1.0484 | 0.1771 | 48 |
| miR-29a-5p | miR-222-5p | 1.1160 | 0.9498 | 1.3112 | 0.1824 | 101 |
| let-7g | miR-944 | 1.1090 | 0.9501 | 1.2945 | 0.1897 | 32 |
| miR-212-3p | miR-450b-5p | 0.9053 | 0.7788 | 1.0524 | 0.1952 | 76 |
| miR-194-3p | miR-431-5p | 1.6587 | 0.7701 | 3.5726 | 0.1962 | 6 |
| miR-148a | miR-187 | 0.8803 | 0.7249 | 1.0690 | 0.1983 | 23 |
| miR-194-3p | miR-222-5p | 1.1812 | 0.9141 | 1.5262 | 0.2029 | 25 |
| miR-34a-5p | miR-187 | 0.8393 | 0.6313 | 1.1159 | 0.2281 | 23 |
| miR-125a-3p | miR-212-3p | 1.1086 | 0.9370 | 1.3115 | 0.2296 | 102 |
| miR-34a-5p | miR-194-3p | 0.8045 | 0.5638 | 1.1480 | 0.2305 | 25 |
| let-7g | miR-222-5p | 1.0765 | 0.9535 | 1.2155 | 0.2337 | 102 |
| miR-146a-5p | miR-222-5p | 1.1165 | 0.9294 | 1.3414 | 0.2391 | 102 |
| miR-148a | miR-194-3p | 1.1476 | 0.9101 | 1.4472 | 0.2445 | 25 |
| miR-148a | miR-675-5p | 1.3606 | 0.7951 | 2.3285 | 0.2612 | 7 |
| miR-205-5p | miR-222-5p | 0.9639 | 0.9038 | 1.0281 | 0.2638 | 93 |
| miR-34a-5p | miR-205-5p | 1.0354 | 0.9730 | 1.1017 | 0.2726 | 93 |
| miR-222-5p | miR-450b-5p | 0.9147 | 0.7792 | 1.0739 | 0.2763 | 76 |
| let-7g | miR-187 | 0.8782 | 0.6941 | 1.1110 | 0.2789 | 23 |
| miR-148a | miR-769-5p | 1.0564 | 0.9557 | 1.1678 | 0.2831 | 102 |
| miR-187 | miR-212-3p | 1.1736 | 0.8746 | 1.5748 | 0.2860 | 23 |
| miR-125a-3p | miR-769-5p | 0.9018 | 0.7446 | 1.0922 | 0.2902 | 101 |
| miR-187 | miR-431-5p | 1.5119 | 0.6706 | 3.4089 | 0.3190 | 6 |
| miR-205-5p | miR-625-5p | 0.9667 | 0.9034 | 1.0344 | 0.3263 | 47 |
| miR-34a-5p | miR-450b-5p | 0.9143 | 0.7616 | 1.0977 | 0.3369 | 76 |
| let-7g | miR-148a | 0.9456 | 0.8434 | 1.0601 | 0.3374 | 103 |
| miR-187 | miR-194-3p | 1.2231 | 0.8091 | 1.8489 | 0.3396 | 7 |
| miR-431-5p | miR-944 | 1.1354 | 0.8715 | 1.4793 | 0.3468 | 17 |
| miR-675-5p | miR-769-5p | 0.6883 | 0.3132 | 1.5125 | 0.3524 | 7 |
| miR-205-5p | miR-212-3p | 0.9723 | 0.9162 | 1.0318 | 0.3535 | 93 |
| let-7g | miR-125a-3p | 1.0601 | 0.9355 | 1.2012 | 0.3601 | 102 |
| miR-625-5p | miR-944 | 1.0902 | 0.9038 | 1.3149 | 0.3667 | 18 |
| miR-34a-5p | miR-125a-3p | 0.9163 | 0.7575 | 1.1085 | 0.3683 | 102 |
| miR-34a-5p | miR-625-5p | 0.9329 | 0.8015 | 1.0858 | 0.3698 | 48 |
| miR-148a | miR-450b-5p | 1.0481 | 0.9420 | 1.1662 | 0.3879 | 76 |
| miR-125a-3p | miR-675-5p | 1.4640 | 0.6080 | 3.5255 | 0.3952 | 7 |
| miR-29a-5p | miR-194-3p | 0.8749 | 0.6401 | 1.1958 | 0.4019 | 25 |
| miR-194-3p | miR-769-5p | 1.1580 | 0.8081 | 1.6596 | 0.4242 | 25 |
| miR-146a-5p | miR-675-5p | 1.5986 | 0.4962 | 5.1499 | 0.4319 | 7 |
| miR-125a-3p | miR-194-3p | 0.9030 | 0.6986 | 1.1671 | 0.4356 | 25 |
| miR-29a-5p | miR-431-5p | 1.0935 | 0.8690 | 1.3761 | 0.4458 | 39 |
| miR-205-5p | miR-944 | 1.0454 | 0.9315 | 1.1733 | 0.4505 | 28 |
| let-7g | miR-146a-5p | 1.0503 | 0.9243 | 1.1935 | 0.4515 | 103 |
| miR-187 | miR-205-5p | 0.9425 | 0.8062 | 1.1018 | 0.4573 | 21 |
| miR-125a-3p | miR-944 | 1.0786 | 0.8831 | 1.3172 | 0.4584 | 32 |
| miR-146a-5p | miR-431-5p | 1.0934 | 0.8633 | 1.3849 | 0.4588 | 39 |
| let-7g | miR-675-5p | 1.1376 | 0.8061 | 1.6053 | 0.4633 | 7 |
| miR-148a | miR-944 | 0.9341 | 0.7783 | 1.1211 | 0.4641 | 32 |
| let-7g | miR-29a-5p | 1.0444 | 0.9270 | 1.1766 | 0.4750 | 102 |
| miR-146a-5p | miR-769-5p | 0.9200 | 0.7296 | 1.1599 | 0.4805 | 102 |
| miR-34a-5p | miR-212-3p | 1.0981 | 0.8418 | 1.4324 | 0.4901 | 103 |
| miR-431-5p | miR-769-5p | 0.9199 | 0.7255 | 1.1664 | 0.4906 | 39 |
| miR-187 | miR-222-5p | 1.0835 | 0.8597 | 1.3654 | 0.4970 | 23 |
| let-7g | miR-194-3p | 1.0868 | 0.8475 | 1.3936 | 0.5118 | 25 |
| miR-125a-3p | miR-222-5p | 1.0521 | 0.9013 | 1.2281 | 0.5200 | 101 |
| let-7g | miR-431-5p | 1.0575 | 0.8891 | 1.2580 | 0.5275 | 39 |
| miR-212-3p | miR-675-5p | 1.3927 | 0.4950 | 3.9188 | 0.5303 | 7 |
| miR-29a-5p | miR-769-5p | 0.9293 | 0.7381 | 1.1702 | 0.5330 | 101 |
| miR-431-5p | miR-450b-5p | 0.9224 | 0.7148 | 1.1903 | 0.5347 | 31 |
| miR-222-5p | miR-431-5p | 1.0693 | 0.8484 | 1.3478 | 0.5703 | 39 |
| miR-450b-5p | miR-769-5p | 0.9453 | 0.7762 | 1.1513 | 0.5760 | 76 |
| miR-222-5p | miR-625-5p | 0.9623 | 0.8410 | 1.1011 | 0.5765 | 48 |
| miR-222-5p | miR-944 | 1.0601 | 0.8599 | 1.3070 | 0.5845 | 32 |
| miR-125a-3p | miR-146a-5p | 0.9464 | 0.7746 | 1.1564 | 0.5901 | 102 |
| miR-222-5p | miR-675-5p | 0.7239 | 0.2174 | 2.4113 | 0.5987 | 7 |
| miR-29a-5p | miR-187 | 1.0820 | 0.7995 | 1.4643 | 0.6097 | 23 |
| miR-194-3p | miR-212-3p | 1.0796 | 0.7938 | 1.4683 | 0.6256 | 25 |
| miR-212-3p | miR-944 | 0.9568 | 0.7980 | 1.1472 | 0.6334 | 32 |
| miR-29a-5p | miR-125a-3p | 1.0369 | 0.8910 | 1.2067 | 0.6395 | 101 |
| let-7g | miR-625-5p | 0.9642 | 0.8250 | 1.1269 | 0.6470 | 48 |
| miR-194-3p | miR-625-5p | 0.8765 | 0.4874 | 1.5764 | 0.6598 | 9 |
| miR-625-5p | miR-769-5p | 1.0342 | 0.8863 | 1.2067 | 0.6694 | 48 |
| miR-125a-3p | miR-450b-5p | 0.9685 | 0.8349 | 1.1234 | 0.6721 | 76 |
| let-7g | miR-450b-5p | 1.0311 | 0.8860 | 1.1999 | 0.6926 | 76 |
| miR-194-3p | miR-944 | 0.8554 | 0.3462 | 2.1135 | 0.7350 | 6 |
| miR-34a-5p | miR-431-5p | 1.0396 | 0.8100 | 1.3344 | 0.7602 | 39 |
| miR-146a-5p | miR-187 | 0.9555 | 0.7127 | 1.2811 | 0.7610 | 23 |
| miR-146a-5p | miR-450b-5p | 1.0296 | 0.8446 | 1.2551 | 0.7726 | 76 |
| miR-125a-3p | miR-625-5p | 0.9780 | 0.8379 | 1.1416 | 0.7781 | 48 |
| miR-34a-5p | miR-944 | 0.9701 | 0.7816 | 1.2041 | 0.7830 | 32 |
| miR-34a-5p | miR-675-5p | 1.1075 | 0.5310 | 2.3101 | 0.7854 | 7 |
| miR-29a-5p | miR-450b-5p | 1.0256 | 0.8315 | 1.2650 | 0.8132 | 76 |
| miR-212-3p | miR-222-5p | 0.9839 | 0.8428 | 1.1485 | 0.8368 | 102 |
| miR-194-3p | miR-450b-5p | 1.0277 | 0.7803 | 1.3536 | 0.8458 | 18 |
| miR-29a-5p | miR-944 | 1.0188 | 0.8231 | 1.2610 | 0.8642 | 32 |
| miR-769-5p | miR-944 | 1.0181 | 0.8272 | 1.2531 | 0.8656 | 32 |
| miR-187 | miR-769-5p | 0.9794 | 0.7591 | 1.2636 | 0.8728 | 23 |
| miR-146a-5p | miR-194-3p | 0.9779 | 0.7373 | 1.2972 | 0.8770 | 25 |
| miR-187 | miR-625-5p | 0.9716 | 0.6630 | 1.4238 | 0.8825 | 10 |
| miR-29a-5p | miR-675-5p | 1.0872 | 0.3297 | 3.5853 | 0.8908 | 7 |
| miR-205-5p | miR-431-5p | 1.0060 | 0.9219 | 1.0978 | 0.8924 | 37 |
| let-7g | miR-769-5p | 1.0080 | 0.8886 | 1.1433 | 0.9019 | 102 |
| miR-146a-5p | miR-625-5p | 0.9898 | 0.8378 | 1.1693 | 0.9036 | 48 |
| miR-125a-3p | miR-187 | 0.9866 | 0.7882 | 1.2349 | 0.9063 | 23 |
| miR-29a-5p | miR-146a-5p | 1.0106 | 0.8373 | 1.2199 | 0.9124 | 102 |
| miR-146a-5p | miR-944 | 1.0068 | 0.8211 | 1.2344 | 0.9482 | 32 |
| miR-187 | miR-450b-5p | 1.0152 | 0.6270 | 1.6437 | 0.9512 | 16 |
| miR-34a-5p | miR-222-5p | 1.0035 | 0.8580 | 1.1736 | 0.9656 | 102 |
| miR-29a-5p | miR-625-5p | 1.0012 | 0.8586 | 1.1676 | 0.9874 | 48 |
| miR-450b-5p | miR-625-5p | 0.9989 | 0.8382 | 1.1903 | 0.9898 | 40 |
| miR-148a | miR-625-5p | 0.9993 | 0.8826 | 1.1315 | 0.9913 | 48 |
| miR-212-3p | miR-431-5p | 1.0003 | 0.7876 | 1.2705 | 0.9980 | 39 |
| miR-187 | miR-675-5p |  |  |  |  | 1 |
| miR-187 | miR-944 |  |  |  |  | 5 |
| miR-194-3p | miR-675-5p |  |  |  |  | 2 |
| miR-205-5p | miR-675-5p |  |  |  |  | 5 |
| miR-431-5p | miR-675-5p |  |  |  |  | 5 |
| miR-450b-5p | miR-675-5p |  |  |  |  | 5 |
| miR-625-5p | miR-675-5p |  |  |  |  | 1 |
| miR-675-5p | miR-944 |  |  |  |  | 3 |

Adjusted (age, sex, tumor stage, ASA-score, tumor differentiation) effects on differences

| **miRNA1** | **miRNA2** | **HR** | **HRlow** | **HRhigh** | **P** | **N** |
| --- | --- | --- | --- | --- | --- | --- |
| miR-34a-5p | miR-769-5p | 0.629 | 0.4689 | 0.8438 | 0.002 | 92 |
| miR-29a-5p | miR-187 | 1.991 | 1.2046 | 3.2908 | 0.0072 | 20 |
| miR-187 | miR-769-5p | 0.5357 | 0.3308 | 0.8673 | 0.0111 | 20 |
| miR-187 | miR-205-5p | 0.7222 | 0.5574 | 0.9357 | 0.0138 | 19 |
| miR-212-3p | miR-769-5p | 0.7543 | 0.6007 | 0.9473 | 0.0153 | 92 |
| miR-148a | miR-212-3p | 1.1759 | 1.0306 | 1.3417 | 0.016 | 93 |
| miR-450b-5p | miR-944 | 1.5595 | 1.0595 | 2.2954 | 0.0243 | 24 |
| miR-34a-5p | miR-148a | 0.8627 | 0.7526 | 0.989 | 0.0341 | 93 |
| miR-146a-5p | miR-212-3p | 1.2876 | 1.0189 | 1.6272 | 0.0343 | 93 |
| miR-148a | miR-431-5p | 1.322 | 1.0178 | 1.7171 | 0.0364 | 34 |
| miR-146a-5p | miR-187 | 1.5723 | 1.0127 | 2.4411 | 0.0438 | 20 |
| miR-222-5p | miR-769-5p | 0.8384 | 0.7033 | 0.9993 | 0.0491 | 92 |
| miR-29a-5p | miR-212-3p | 1.1849 | 0.9967 | 1.4087 | 0.0546 | 92 |
| miR-212-3p | miR-450b-5p | 0.8219 | 0.6707 | 1.0071 | 0.0585 | 67 |
| miR-34a-5p | miR-146a-5p | 0.745 | 0.5487 | 1.0115 | 0.0592 | 93 |
| miR-125a-3p | miR-187 | 1.4183 | 0.9848 | 2.0426 | 0.0605 | 20 |
| miR-194-3p | miR-222-5p | 1.3367 | 0.9677 | 1.8465 | 0.0783 | 22 |
| let-7g | miR-944 | 1.2118 | 0.976 | 1.5044 | 0.0818 | 29 |
| let-7g | miR-212-3p | 1.1363 | 0.9812 | 1.3159 | 0.0879 | 93 |
| miR-205-5p | miR-769-5p | 0.9386 | 0.8724 | 1.0098 | 0.0893 | 84 |
| miR-625-5p | miR-944 | 1.3864 | 0.9441 | 2.0359 | 0.0956 | 16 |
| miR-431-5p | miR-625-5p | 0.7841 | 0.585 | 1.0508 | 0.1035 | 21 |
| miR-29a-5p | miR-205-5p | 1.0585 | 0.9884 | 1.1336 | 0.1041 | 83 |
| miR-29a-5p | miR-222-5p | 1.1489 | 0.971 | 1.3595 | 0.1059 | 91 |
| miR-148a | miR-222-5p | 1.0818 | 0.9821 | 1.1916 | 0.111 | 92 |
| miR-125a-3p | miR-212-3p | 1.1682 | 0.9648 | 1.4146 | 0.1112 | 92 |
| miR-148a | miR-205-5p | 1.047 | 0.9894 | 1.1079 | 0.1115 | 84 |
| miR-29a-5p | miR-34a-5p | 1.1605 | 0.9629 | 1.3986 | 0.1181 | 92 |
| miR-34a-5p | miR-450b-5p | 0.8286 | 0.6521 | 1.0527 | 0.1237 | 67 |
| miR-148a | miR-194-3p | 1.3085 | 0.924 | 1.8529 | 0.1298 | 22 |
| miR-34a-5p | miR-194-3p | 0.6487 | 0.3697 | 1.1382 | 0.1314 | 22 |
| miR-146a-5p | miR-222-5p | 1.1611 | 0.9479 | 1.4221 | 0.149 | 92 |
| let-7g | miR-34a-5p | 1.1223 | 0.9588 | 1.3138 | 0.151 | 93 |
| miR-125a-3p | miR-431-5p | 1.3599 | 0.8924 | 2.0725 | 0.1527 | 34 |
| miR-125a-3p | miR-944 | 1.2096 | 0.931 | 1.5717 | 0.1543 | 29 |
| miR-212-3p | miR-625-5p | 0.8654 | 0.7078 | 1.0582 | 0.1589 | 44 |
| miR-146a-5p | miR-769-5p | 0.8275 | 0.6302 | 1.0866 | 0.1731 | 92 |
| miR-125a-3p | miR-205-5p | 1.0531 | 0.9772 | 1.1348 | 0.1751 | 83 |
| miR-205-5p | miR-450b-5p | 0.9494 | 0.8802 | 1.024 | 0.1783 | 64 |
| miR-222-5p | miR-944 | 1.2062 | 0.9165 | 1.5874 | 0.1809 | 29 |
| miR-146a-5p | miR-205-5p | 1.0478 | 0.9781 | 1.1224 | 0.1838 | 84 |
| miR-187 | miR-450b-5p | *>*10000 | *<*0.0001 | *>*10000 | 0.1885 | 13 |
| miR-187 | miR-212-3p | 0.7461 | 0.4771 | 1.1668 | 0.1992 | 20 |
| miR-34a-5p | miR-125a-3p | 0.8711 | 0.7034 | 1.0789 | 0.2062 | 92 |
| let-7g | miR-205-5p | 1.0403 | 0.9768 | 1.1079 | 0.2192 | 84 |
| miR-125a-3p | miR-148a | 0.9346 | 0.8382 | 1.0421 | 0.2233 | 92 |
| miR-222-5p | miR-450b-5p | 0.8895 | 0.7362 | 1.0747 | 0.225 | 67 |
| miR-222-5p | miR-431-5p | 1.2166 | 0.8836 | 1.6752 | 0.2296 | 34 |
| miR-125a-3p | miR-769-5p | 0.8756 | 0.7024 | 1.0916 | 0.2377 | 91 |
| let-7g | miR-431-5p | 1.1594 | 0.9048 | 1.4857 | 0.2424 | 34 |
| miR-34a-5p | miR-187 | 1.2659 | 0.8431 | 1.9009 | 0.2555 | 20 |
| miR-194-3p | miR-205-5p | 1.1481 | 0.9045 | 1.4573 | 0.2563 | 19 |
| miR-194-3p | miR-212-3p | 1.308 | 0.8228 | 2.0795 | 0.2563 | 22 |
| miR-146a-5p | miR-148a | 0.9379 | 0.8388 | 1.0487 | 0.2603 | 93 |
| miR-125a-3p | miR-222-5p | 1.1018 | 0.9266 | 1.3103 | 0.2725 | 91 |
| miR-29a-5p | miR-148a | 0.9418 | 0.8444 | 1.0504 | 0.2814 | 92 |
| miR-29a-5p | miR-769-5p | 0.8671 | 0.668 | 1.1254 | 0.2838 | 91 |
| let-7g | miR-222-5p | 1.0703 | 0.9378 | 1.2215 | 0.314 | 92 |
| miR-148a | miR-450b-5p | 1.0655 | 0.9404 | 1.2072 | 0.3194 | 67 |
| miR-34a-5p | miR-205-5p | 1.0358 | 0.9656 | 1.1112 | 0.3259 | 84 |
| miR-769-5p | miR-944 | 1.144 | 0.8691 | 1.5058 | 0.3375 | 29 |
| miR-205-5p | miR-222-5p | 0.9667 | 0.9002 | 1.0381 | 0.352 | 84 |
| let-7g | miR-187 | 0.804 | 0.506 | 1.2774 | 0.3557 | 20 |
| miR-29a-5p | miR-944 | 1.127 | 0.8694 | 1.4609 | 0.3667 | 29 |
| let-7g | miR-148a | 0.9443 | 0.8324 | 1.0712 | 0.3729 | 93 |
| miR-34a-5p | miR-212-3p | 1.1444 | 0.8484 | 1.5438 | 0.377 | 93 |
| miR-34a-5p | miR-625-5p | 0.9215 | 0.7594 | 1.1183 | 0.4079 | 44 |
| miR-205-5p | miR-212-3p | 0.9726 | 0.9095 | 1.0401 | 0.4174 | 84 |
| miR-212-3p | miR-431-5p | 1.1448 | 0.8226 | 1.5933 | 0.4224 | 34 |
| miR-146a-5p | miR-431-5p | 1.1428 | 0.8206 | 1.5916 | 0.4295 | 34 |
| miR-34a-5p | miR-431-5p | 1.1542 | 0.7916 | 1.6828 | 0.4561 | 34 |
| miR-146a-5p | miR-944 | 1.1016 | 0.8479 | 1.4312 | 0.4688 | 29 |
| miR-125a-3p | miR-194-3p | 0.8794 | 0.6161 | 1.2553 | 0.4791 | 22 |
| miR-450b-5p | miR-769-5p | 0.9237 | 0.7228 | 1.1805 | 0.5262 | 67 |
| let-7g | miR-125a-3p | 1.0464 | 0.9072 | 1.2068 | 0.5335 | 92 |
| miR-148a | miR-769-5p | 1.0326 | 0.9261 | 1.1513 | 0.5633 | 92 |
| miR-194-3p | miR-450b-5p | 0.8859 | 0.5869 | 1.3371 | 0.564 | 16 |
| miR-29a-5p | miR-431-5p | 1.0856 | 0.8206 | 1.4363 | 0.565 | 34 |
| miR-431-5p | miR-769-5p | 0.9156 | 0.6732 | 1.2454 | 0.5744 | 34 |
| miR-29a-5p | miR-194-3p | 0.8736 | 0.5413 | 1.4099 | 0.58 | 22 |
| let-7g | miR-769-5p | 0.9616 | 0.8369 | 1.1048 | 0.5804 | 92 |
| miR-431-5p | miR-450b-5p | 0.9205 | 0.681 | 1.2444 | 0.5903 | 26 |
| miR-148a | miR-944 | 1.0689 | 0.8308 | 1.3754 | 0.6043 | 29 |
| miR-205-5p | miR-625-5p | 0.9789 | 0.9022 | 1.062 | 0.6075 | 43 |
| let-7g | miR-146a-5p | 1.0375 | 0.8977 | 1.1992 | 0.6181 | 93 |
| miR-125a-3p | miR-450b-5p | 0.9541 | 0.7871 | 1.1566 | 0.6322 | 67 |
| miR-194-3p | miR-769-5p | 1.1408 | 0.6571 | 1.9806 | 0.6399 | 22 |
| let-7g | miR-29a-5p | 1.0294 | 0.8995 | 1.178 | 0.6739 | 92 |
| miR-34a-5p | miR-944 | 1.0601 | 0.8004 | 1.404 | 0.6839 | 29 |
| miR-625-5p | miR-769-5p | 1.0348 | 0.8628 | 1.2412 | 0.712 | 44 |
| miR-34a-5p | miR-222-5p | 1.031 | 0.8718 | 1.2193 | 0.7214 | 92 |
| miR-450b-5p | miR-625-5p | 0.964 | 0.7792 | 1.1927 | 0.7358 | 36 |
| miR-222-5p | miR-625-5p | 0.9698 | 0.8036 | 1.1702 | 0.7487 | 44 |
| let-7g | miR-450b-5p | 1.0251 | 0.8622 | 1.2187 | 0.7792 | 67 |
| miR-148a | miR-625-5p | 1.0255 | 0.8593 | 1.2239 | 0.7803 | 44 |
| miR-29a-5p | miR-125a-3p | 1.0249 | 0.8602 | 1.2213 | 0.7829 | 91 |
| miR-205-5p | miR-431-5p | 0.9832 | 0.8699 | 1.1112 | 0.7856 | 32 |
| miR-431-5p | miR-944 | 1.0622 | 0.6335 | 1.7807 | 0.8191 | 15 |
| miR-29a-5p | miR-625-5p | 0.9799 | 0.8101 | 1.1853 | 0.8345 | 44 |
| miR-187 | miR-222-5p | 0.9657 | 0.6892 | 1.3533 | 0.8395 | 20 |
| miR-146a-5p | miR-194-3p | 0.9683 | 0.6821 | 1.3747 | 0.8571 | 22 |
| miR-29a-5p | miR-146a-5p | 1.0171 | 0.8192 | 1.2628 | 0.8777 | 92 |
| miR-212-3p | miR-944 | 1.0174 | 0.8109 | 1.2763 | 0.8818 | 29 |
| miR-29a-5p | miR-450b-5p | 1.0184 | 0.7878 | 1.3164 | 0.8896 | 67 |
| miR-125a-3p | miR-146a-5p | 0.9846 | 0.7828 | 1.2384 | 0.8946 | 92 |
| let-7g | miR-625-5p | 1.0125 | 0.8211 | 1.2486 | 0.9072 | 44 |
| miR-205-5p | miR-944 | 1.0079 | 0.8623 | 1.1781 | 0.9213 | 25 |
| miR-125a-3p | miR-625-5p | 0.9946 | 0.8341 | 1.1859 | 0.9517 | 44 |
| miR-146a-5p | miR-625-5p | 1.0064 | 0.8137 | 1.2448 | 0.953 | 44 |
| miR-146a-5p | miR-450b-5p | 0.9932 | 0.7627 | 1.2935 | 0.9599 | 67 |
| miR-212-3p | miR-222-5p | 1.003 | 0.8519 | 1.1809 | 0.9715 | 92 |
| miR-148a | miR-187 | 1.0039 | 0.7499 | 1.3437 | 0.9794 | 20 |
| let-7g | miR-194-3p | 0.9976 | 0.698 | 1.4259 | 0.9897 | 22 |
| miR-194-3p | miR-625-5p | *<*0.0001 | *<*0.0001 | *>*10000 | 0.9901 | 9 |
| miR-187 | miR-194-3p | *>*10000 | *<*0.0001 | *>*10000 | 0.9984 | 6 |
| miR-187 | miR-625-5p | *<*0.0001 | *<*0.0001 | *>*10000 | 0.9986 | 10 |
| miR-194-3p | miR-944 | *<*0.0001 | *<*0.0001 | *>*10000 | 0.9994 | 6 |
| let-7g | miR-675-5p | *>*10000 | *<*0.0001 | *>*10000 | 0.9996 | 6 |
| miR-29a-5p | miR-675-5p | *>*10000 | *<*0.0001 | *>*10000 | 0.9996 | 6 |
| miR-34a-5p | miR-675-5p | *>*10000 | *<*0.0001 | *>*10000 | 0.9996 | 6 |
| miR-125a-3p | miR-675-5p | *>*10000 | *<*0.0001 | *>*10000 | 0.9996 | 6 |
| miR-146a-5p | miR-675-5p | *>*10000 | *<*0.0001 | *>*10000 | 0.9996 | 6 |
| miR-148a | miR-675-5p | *>*10000 | *<*0.0001 | *>*10000 | 0.9996 | 6 |
| miR-212-3p | miR-675-5p | *>*10000 | *<*0.0001 | *>*10000 | 0.9996 | 6 |
| miR-222-5p | miR-675-5p | *>*10000 | *<*0.0001 | *>*10000 | 0.9996 | 6 |
| miR-675-5p | miR-769-5p | *<*0.0001 | *<*0.0001 | *>*10000 | 0.9996 | 6 |
| miR-194-3p | miR-431-5p | *>*10000 | *<*0.0001 | *>*10000 | 0.9997 | 6 |
| miR-187 | miR-431-5p | 2.1505 | *<*0.0001 | *>*10000 | 1 | 5 |
| miR-187 | miR-675-5p |  |  |  |  | 1 |
| miR-187 | miR-944 |  |  |  |  | 5 |
| miR-194-3p | miR-675-5p |  |  |  |  | 2 |
| miR-205-5p | miR-675-5p |  |  |  |  | 5 |
| miR-431-5p | miR-675-5p |  |  |  |  | 5 |
| miR-450b-5p | miR-675-5p |  |  |  |  | 5 |
| miR-625-5p | miR-675-5p |  |  |  |  | 1 |
| miR-675-5p | miR-944 |  |  |  |  | 3 |

**Prognosis on diagnosis: A-AC**

In the following miRNAs univariately are considered

Unadjusted univariate effects

| **miRNA** | **HR** | **HRlow** | **HRhigh** | **P** | **N** |
| --- | --- | --- | --- | --- | --- |
| let-7g | 0.7365 | 0.5836 | 0.9296 | 0.0100 | 53 |
| miR-29a-5p | 0.9121 | 0.7586 | 1.0966 | 0.3278 | 54 |
| miR-34a-5p | 0.6595 | 0.4622 | 0.9411 | 0.0218 | 53 |
| miR-125a-3p | 0.8310 | 0.6620 | 1.0431 | 0.1105 | 51 |
| miR-146a-5p | 0.9001 | 0.7488 | 1.0820 | 0.2626 | 54 |
| miR-148a | 0.8987 | 0.7458 | 1.0828 | 0.2613 | 53 |
| miR-187 | 1.5053 | 1.0112 | 2.2408 | 0.0439 | 24 |
| miR-194-3p | 1.1027 | 0.6985 | 1.7408 | 0.6746 | 25 |
| miR-205-5p | 0.7346 | 0.6269 | 0.8607 | 0.0001 | 37 |
| miR-212-3p | 0.8578 | 0.7087 | 1.0384 | 0.1155 | 53 |
| miR-222-5p | 1.0295 | 0.8050 | 1.3167 | 0.8168 | 50 |
| miR-431-5p | 0.9354 | 0.5060 | 1.7291 | 0.8312 | 8 |
| miR-450b-5p | 0.8479 | 0.5559 | 1.2934 | 0.4439 | 27 |
| miR-625-5p | 1.0927 | 0.8200 | 1.4561 | 0.5451 | 31 |
| miR-675-5p |  |  |  |  | 1 |
| miR-769-5p | 1.1732 | 0.8382 | 1.6422 | 0.3518 | 53 |
| miR-944 | 1.2440 | 0.6063 | 2.5524 | 0.5516 | 6 |

Adjusted (age, sex, tumor stage, ASA-score, tumor differentiation) univariate effects

| **miRNA** | **HR** | **HRlow** | **HRhigh** | **P** | **N** |
| --- | --- | --- | --- | --- | --- |
| let-7g | 0.866 | 0.6592 | 1.1377 | 0.3014 | 52 |
| miR-29a-5p | 0.8333 | 0.6685 | 1.0387 | 0.1047 | 53 |
| miR-34a-5p | 0.5834 | 0.383 | 0.8887 | 0.0121 | 52 |
| miR-125a-3p | 0.796 | 0.6219 | 1.0189 | 0.0701 | 50 |
| miR-146a-5p | 0.8749 | 0.7016 | 1.0909 | 0.2351 | 53 |
| miR-148a | 0.9062 | 0.7463 | 1.1004 | 0.3201 | 52 |
| miR-187 | 2.3375 | 1.2205 | 4.4768 | 0.0104 | 24 |
| miR-194-3p | 0.9773 | 0.5273 | 1.8112 | 0.9419 | 24 |
| miR-205-5p | 0.8286 | 0.6743 | 1.0181 | 0.0736 | 36 |
| miR-212-3p | 0.8561 | 0.6888 | 1.0639 | 0.1612 | 52 |
| miR-222-5p | 1.0238 | 0.7773 | 1.3483 | 0.8673 | 49 |
| miR-431-5p | *<*0.0001 | *<*0.0001 | *>*10000 | 0.9999 | 8 |
| miR-450b-5p | 0.4795 | 0.2331 | 0.9864 | 0.0458 | 26 |
| miR-625-5p | 1.3401 | 0.9949 | 1.8051 | 0.054 | 30 |
| miR-675-5p |  |  |  |  | 1 |
| miR-769-5p | 1.3179 | 0.8872 | 1.9578 | 0.1716 | 52 |
| miR-944 | *>*10000 | *<*0.0001 | *>*10000 | 0.9352 | 6 |

Unadjusted univariate effects (under median vs. over median)

| **miRNA** | **HR** | **HRlow** | **HRhigh** | **P** | **N** |
| --- | --- | --- | --- | --- | --- |
| let-7g | 0.5483 | 0.2611 | 1.1518 | 0.1126 | 53 |
| miR-29a-5p | 0.7833 | 0.3775 | 1.6253 | 0.5119 | 54 |
| miR-34a-5p | 0.4027 | 0.1892 | 0.8571 | 0.0183 | 53 |
| miR-125a-3p | 0.9815 | 0.4733 | 2.0351 | 0.9599 | 51 |
| miR-146a-5p | 0.6956 | 0.3341 | 1.4480 | 0.3318 | 54 |
| miR-148a | 0.8879 | 0.4269 | 1.8466 | 0.7503 | 53 |
| miR-187 | 1.3845 | 0.4733 | 4.0499 | 0.5524 | 24 |
| miR-194-3p | 0.6602 | 0.2010 | 2.1687 | 0.4938 | 25 |
| miR-205-5p | 0.4609 | 0.2032 | 1.0456 | 0.0638 | 37 |
| miR-212-3p | 0.6473 | 0.3107 | 1.3485 | 0.2454 | 53 |
| miR-222-5p | 0.7617 | 0.3561 | 1.6293 | 0.4829 | 50 |
| miR-431-5p | 1.7835 | 0.2904 | 10.9527 | 0.5321 | 8 |
| miR-450b-5p | 1.3222 | 0.4914 | 3.5577 | 0.5802 | 27 |
| miR-625-5p | 1.1292 | 0.4444 | 2.8694 | 0.7984 | 31 |
| miR-675-5p |  |  |  |  | 1 |
| miR-769-5p | 1.0203 | 0.4904 | 2.1229 | 0.9571 | 53 |
| miR-944 | 1.4368 | 0.2342 | 8.8158 | 0.6954 | 6 |

Adjusted (age, sex, tumor stage, ASA-score, tumor differentiation) univariate effects (under median vs. over median)

| **miRNA** | **HR** | **HRlow** | **HRhigh** | **P** | **N** |
| --- | --- | --- | --- | --- | --- |
| let-7g | 0.9666 | 0.352 | 2.6546 | 0.9475 | 52 |
| miR-29a-5p | 0.7082 | 0.3122 | 1.6066 | 0.4091 | 53 |
| miR-34a-5p | 0.3646 | 0.1563 | 0.8502 | 0.0195 | 52 |
| miR-125a-3p | 0.6534 | 0.2615 | 1.6329 | 0.3625 | 50 |
| miR-146a-5p | 0.6845 | 0.3056 | 1.5332 | 0.3569 | 53 |
| miR-148a | 0.9091 | 0.3941 | 2.0972 | 0.8233 | 52 |
| miR-187 | 2.2488 | 0.5563 | 9.0905 | 0.2555 | 24 |
| miR-194-3p | 0.812 | 0.1833 | 3.5969 | 0.7838 | 24 |
| miR-205-5p | 0.6246 | 0.1806 | 2.1606 | 0.4573 | 36 |
| miR-212-3p | 0.7936 | 0.3397 | 1.8538 | 0.5934 | 52 |
| miR-222-5p | 0.5776 | 0.2425 | 1.376 | 0.2152 | 49 |
| miR-431-5p | *>*10000 | *<*0.0001 | *>*10000 | 0.9999 | 8 |
| miR-450b-5p | 1.0083 | 0.16 | 6.355 | 0.9930 | 26 |
| miR-625-5p | 1.5033 | 0.5355 | 4.2198 | 0.4389 | 30 |
| miR-675-5p |  |  |  |  | 1 |
| miR-769-5p | 0.7813 | 0.3495 | 1.7468 | 0.5478 | 52 |
| miR-944 | *>*10000 | *<*0.0001 | *>*10000 | 1.0000 | 6 |

**Differences in microRNAs**

In the following differences of miRNAs are considered. They are defined as miRNA1-miRNA2.

Unadjusted effects on differences

| **miRNA1** | **miRNA2** | **HR** | **HRlow** | **HRhigh** | **P** | **N** |
| --- | --- | --- | --- | --- | --- | --- |
| miR-205-5p | miR-769-5p | 0.7102 | 0.601 | 0.8394 | *<*0.0001 | 36 |
| miR-34a-5p | miR-187 | 0.4431 | 0.2716 | 0.723 | 0.0011 | 24 |
| miR-148a | miR-205-5p | 1.2534 | 1.0873 | 1.4449 | 0.0018 | 37 |
| miR-125a-3p | miR-187 | 0.6892 | 0.5384 | 0.8824 | 0.0032 | 23 |
| miR-187 | miR-205-5p | 1.3529 | 1.1006 | 1.6632 | 0.0041 | 17 |
| miR-187 | miR-212-3p | 2.2175 | 1.286 | 3.8239 | 0.0042 | 24 |
| miR-205-5p | miR-450b-5p | 0.7309 | 0.5886 | 0.9075 | 0.0045 | 22 |
| let-7g | miR-205-5p | 1.2752 | 1.0723 | 1.5164 | 0.006 | 37 |
| miR-146a-5p | miR-205-5p | 1.1873 | 1.0492 | 1.3436 | 0.0065 | 37 |
| let-7g | miR-769-5p | 0.7410 | 0.5931 | 0.9256 | 0.0083 | 52 |
| miR-34a-5p | miR-769-5p | 0.6644 | 0.4825 | 0.9149 | 0.0122 | 52 |
| miR-34a-5p | miR-205-5p | 1.2197 | 1.0434 | 1.4257 | 0.0126 | 37 |
| let-7g | miR-187 | 0.7698 | 0.6211 | 0.9543 | 0.017 | 24 |
| let-7g | miR-625-5p | 0.7412 | 0.5789 | 0.949 | 0.0175 | 31 |
| miR-125a-3p | miR-205-5p | 1.2107 | 1.0271 | 1.4272 | 0.0227 | 36 |
| let-7g | miR-222-5p | 0.8043 | 0.6655 | 0.972 | 0.0242 | 50 |
| miR-29a-5p | miR-187 | 0.7389 | 0.5649 | 0.9665 | 0.0272 | 24 |
| miR-205-5p | miR-212-3p | 0.8584 | 0.7486 | 0.9844 | 0.0289 | 37 |
| miR-146a-5p | miR-187 | 0.6683 | 0.4635 | 0.9635 | 0.0308 | 24 |
| miR-187 | miR-769-5p | 1.4705 | 1.0241 | 2.1114 | 0.0367 | 24 |
| miR-450b-5p | miR-769-5p | 0.5934 | 0.353 | 0.9975 | 0.0489 | 27 |
| miR-29a-5p | miR-205-5p | 1.0940 | 0.9973 | 1.2001 | 0.0571 | 37 |
| miR-194-3p | miR-205-5p | 1.5464 | 0.9794 | 2.4417 | 0.0614 | 15 |
| miR-125a-3p | miR-769-5p | 0.7807 | 0.6021 | 1.0124 | 0.0619 | 50 |
| miR-187 | miR-450b-5p | 1.6100 | 0.9746 | 2.6597 | 0.063 | 10 |
| miR-125a-3p | miR-944 | 0.4158 | 0.1568 | 1.1026 | 0.0778 | 6 |
| miR-212-3p | miR-769-5p | 0.8254 | 0.6612 | 1.0302 | 0.0897 | 52 |
| miR-187 | miR-194-3p | 1.4820 | 0.9307 | 2.3599 | 0.0974 | 14 |
| miR-187 | miR-222-5p | 1.3157 | 0.9503 | 1.8216 | 0.0983 | 24 |
| miR-146a-5p | miR-212-3p | 1.2869 | 0.9513 | 1.7408 | 0.1018 | 53 |
| miR-34a-5p | miR-222-5p | 0.8195 | 0.6435 | 1.0435 | 0.1064 | 49 |
| miR-34a-5p | miR-146a-5p | 0.7729 | 0.5626 | 1.062 | 0.112 | 53 |
| miR-212-3p | miR-222-5p | 0.8214 | 0.6392 | 1.0556 | 0.1242 | 49 |
| miR-431-5p | miR-450b-5p | 27.6318 | 0.3588 | 2127.6949 | 0.1343 | 6 |
| miR-146a-5p | miR-450b-5p | 1.3130 | 0.9157 | 1.8826 | 0.1386 | 27 |
| miR-205-5p | miR-431-5p | 0.7823 | 0.5647 | 1.0839 | 0.14 | 6 |
| miR-194-3p | miR-625-5p | 1.6571 | 0.8383 | 3.2756 | 0.1463 | 16 |
| miR-125a-3p | miR-222-5p | 0.8527 | 0.6792 | 1.0705 | 0.1698 | 47 |
| let-7g | miR-450b-5p | 0.8276 | 0.6305 | 1.0863 | 0.1727 | 27 |
| miR-148a | miR-187 | 0.8573 | 0.6807 | 1.0796 | 0.1905 | 24 |
| miR-29a-5p | miR-769-5p | 0.8454 | 0.6516 | 1.0969 | 0.2063 | 53 |
| miR-222-5p | miR-450b-5p | 1.2241 | 0.8941 | 1.676 | 0.2071 | 26 |
| miR-125a-3p | miR-625-5p | 0.8813 | 0.7235 | 1.0735 | 0.2093 | 29 |
| miR-34a-5p | miR-625-5p | 0.8552 | 0.6674 | 1.0957 | 0.216 | 31 |
| miR-148a | miR-769-5p | 0.9111 | 0.7807 | 1.0633 | 0.2376 | 52 |
| let-7g | miR-194-3p | 0.8128 | 0.5667 | 1.1658 | 0.2601 | 25 |
| miR-29a-5p | miR-625-5p | 0.8823 | 0.7076 | 1.1 | 0.2657 | 31 |
| miR-146a-5p | miR-769-5p | 0.8869 | 0.7107 | 1.1069 | 0.2885 | 53 |
| miR-34a-5p | miR-148a | 0.8823 | 0.6987 | 1.1142 | 0.293 | 52 |
| miR-212-3p | miR-431-5p | 0.3625 | 0.0542 | 2.4233 | 0.2952 | 8 |
| miR-148a | miR-944 | 0.7119 | 0.3721 | 1.362 | 0.3047 | 6 |
| miR-212-3p | miR-944 | 0.7862 | 0.4904 | 1.2605 | 0.318 | 6 |
| miR-148a | miR-222-5p | 0.9345 | 0.8159 | 1.0703 | 0.3278 | 50 |
| miR-125a-3p | miR-146a-5p | 0.8917 | 0.7082 | 1.1227 | 0.3293 | 51 |
| miR-29a-5p | miR-450b-5p | 1.1609 | 0.8576 | 1.5715 | 0.3342 | 27 |
| miR-146a-5p | miR-222-5p | 0.9020 | 0.7271 | 1.1189 | 0.3481 | 50 |
| miR-29a-5p | miR-944 | 0.7309 | 0.3772 | 1.4162 | 0.353 | 6 |
| miR-34a-5p | miR-944 | 0.7973 | 0.4861 | 1.3079 | 0.3698 | 6 |
| miR-187 | miR-625-5p | 1.1264 | 0.8672 | 1.4631 | 0.3723 | 18 |
| miR-148a | miR-212-3p | 1.0882 | 0.9028 | 1.3117 | 0.3751 | 52 |
| miR-148a | miR-450b-5p | 1.1364 | 0.8547 | 1.5109 | 0.3789 | 27 |
| miR-125a-3p | miR-450b-5p | 0.8634 | 0.6147 | 1.2127 | 0.3968 | 27 |
| let-7g | miR-944 | 0.0005 | *<*0.0001 | *>*10000 | 0.4015 | 6 |
| miR-34a-5p | miR-194-3p | 0.8516 | 0.577 | 1.2567 | 0.4184 | 24 |
| miR-29a-5p | miR-222-5p | 0.9194 | 0.7439 | 1.1363 | 0.4366 | 50 |
| miR-194-3p | miR-212-3p | 1.1205 | 0.8247 | 1.5223 | 0.4669 | 24 |
| miR-769-5p | miR-944 | 0.7637 | 0.3677 | 1.5859 | 0.4696 | 6 |
| miR-212-3p | miR-625-5p | 0.9200 | 0.7252 | 1.167 | 0.4918 | 31 |
| miR-125a-3p | miR-431-5p | 0.7119 | 0.2626 | 1.9299 | 0.5042 | 8 |
| miR-148a | miR-194-3p | 0.9386 | 0.7782 | 1.132 | 0.5072 | 25 |
| miR-146a-5p | miR-944 | 0.8533 | 0.5226 | 1.3932 | 0.5259 | 6 |
| miR-194-3p | miR-450b-5p | 1.1785 | 0.7072 | 1.9638 | 0.5285 | 14 |
| miR-431-5p | miR-769-5p | 0.7958 | 0.3836 | 1.6509 | 0.5396 | 8 |
| miR-194-3p | miR-769-5p | 1.1735 | 0.703 | 1.9588 | 0.5406 | 25 |
| miR-146a-5p | miR-194-3p | 0.9377 | 0.7623 | 1.1536 | 0.543 | 25 |
| miR-125a-3p | miR-148a | 0.9503 | 0.8045 | 1.1226 | 0.5491 | 50 |
| miR-125a-3p | miR-194-3p | 0.9164 | 0.6769 | 1.2405 | 0.5719 | 22 |
| miR-29a-5p | miR-194-3p | 0.8981 | 0.6143 | 1.313 | 0.5792 | 25 |
| let-7g | miR-29a-5p | 0.9656 | 0.8527 | 1.0933 | 0.5803 | 53 |
| miR-212-3p | miR-450b-5p | 1.1062 | 0.751 | 1.6293 | 0.6096 | 27 |
| miR-222-5p | miR-944 | 0.8908 | 0.5517 | 1.4383 | 0.6362 | 6 |
| let-7g | miR-146a-5p | 0.9668 | 0.8286 | 1.1281 | 0.6679 | 53 |
| let-7g | miR-125a-3p | 0.9461 | 0.7302 | 1.2259 | 0.6752 | 50 |
| let-7g | miR-431-5p | 0.9050 | 0.5391 | 1.5192 | 0.7056 | 8 |
| miR-146a-5p | miR-625-5p | 1.0482 | 0.7976 | 1.3777 | 0.7355 | 31 |
| miR-34a-5p | miR-212-3p | 1.0497 | 0.7702 | 1.4306 | 0.7589 | 53 |
| miR-29a-5p | miR-431-5p | 1.1166 | 0.5462 | 2.2826 | 0.7625 | 8 |
| miR-29a-5p | miR-146a-5p | 1.0168 | 0.9034 | 1.1443 | 0.7827 | 54 |
| miR-625-5p | miR-769-5p | 1.0399 | 0.7853 | 1.3771 | 0.7847 | 31 |
| miR-29a-5p | miR-148a | 1.0116 | 0.9227 | 1.1092 | 0.8053 | 53 |
| miR-125a-3p | miR-212-3p | 1.0270 | 0.8267 | 1.2758 | 0.8097 | 51 |
| miR-450b-5p | miR-625-5p | 0.9612 | 0.693 | 1.3332 | 0.8125 | 20 |
| let-7g | miR-148a | 0.9833 | 0.8495 | 1.1381 | 0.8212 | 53 |
| let-7g | miR-34a-5p | 0.9700 | 0.7418 | 1.2684 | 0.8241 | 52 |
| miR-222-5p | miR-769-5p | 0.9736 | 0.7549 | 1.2557 | 0.8369 | 50 |
| miR-146a-5p | miR-431-5p | 1.1157 | 0.3853 | 3.2306 | 0.8401 | 8 |
| miR-29a-5p | miR-212-3p | 1.0160 | 0.8653 | 1.1929 | 0.8466 | 53 |
| miR-146a-5p | miR-148a | 1.0154 | 0.8596 | 1.1994 | 0.8573 | 53 |
| miR-148a | miR-625-5p | 0.9839 | 0.7831 | 1.2362 | 0.8891 | 31 |
| miR-222-5p | miR-625-5p | 0.9755 | 0.6711 | 1.4178 | 0.8965 | 30 |
| let-7g | miR-212-3p | 0.9885 | 0.8104 | 1.2058 | 0.9091 | 52 |
| miR-29a-5p | miR-125a-3p | 1.0072 | 0.8631 | 1.1753 | 0.9276 | 51 |
| miR-34a-5p | miR-125a-3p | 0.9884 | 0.7587 | 1.2877 | 0.931 | 51 |
| miR-34a-5p | miR-431-5p | 1.0797 | 0.1846 | 6.3167 | 0.9322 | 8 |
| miR-222-5p | miR-431-5p | 0.9797 | 0.5846 | 1.6417 | 0.9378 | 8 |
| miR-194-3p | miR-222-5p | 1.0095 | 0.6859 | 1.4858 | 0.9617 | 25 |
| miR-34a-5p | miR-450b-5p | 1.0110 | 0.6286 | 1.626 | 0.9641 | 27 |
| miR-29a-5p | miR-34a-5p | 1.0042 | 0.8297 | 1.2156 | 0.9653 | 53 |
| miR-148a | miR-431-5p | 1.0083 | 0.5362 | 1.8963 | 0.9794 | 8 |
| miR-205-5p | miR-222-5p | 0.7516 | 0.643 | 0.8785 | 3e-04 | 34 |
| miR-205-5p | miR-625-5p | 0.7422 | 0.6275 | 0.8779 | 5e-04 | 25 |
| let-7g | miR-675-5p |  |  |  |  | 1 |
| miR-29a-5p | miR-675-5p |  |  |  |  | 1 |
| miR-34a-5p | miR-675-5p |  |  |  |  | 1 |
| miR-125a-3p | miR-675-5p |  |  |  |  | 1 |
| miR-146a-5p | miR-675-5p |  |  |  |  | 1 |
| miR-148a | miR-675-5p |  |  |  |  | 1 |
| miR-187 | miR-431-5p |  |  |  |  | 3 |
| miR-187 | miR-675-5p |  |  |  |  | 0 |
| miR-187 | miR-944 |  |  |  |  | 3 |
| miR-194-3p | miR-431-5p |  |  |  |  | 4 |
| miR-194-3p | miR-675-5p |  |  |  |  | 0 |
| miR-194-3p | miR-944 |  |  |  |  | 4 |
| miR-205-5p | miR-675-5p |  |  |  |  | 1 |
| miR-205-5p | miR-944 |  |  |  |  | 4 |
| miR-212-3p | miR-675-5p |  |  |  |  | 1 |
| miR-222-5p | miR-675-5p |  |  |  |  | 1 |
| miR-431-5p | miR-625-5p |  |  |  |  | 3 |
| miR-431-5p | miR-675-5p |  |  |  |  | 0 |
| miR-431-5p | miR-944 |  |  |  |  | 2 |
| miR-450b-5p | miR-675-5p |  |  |  |  | 1 |
| miR-450b-5p | miR-944 |  |  |  |  | 4 |
| miR-625-5p | miR-675-5p |  |  |  |  | 1 |
| miR-625-5p | miR-944 |  |  |  |  | 4 |
| miR-675-5p | miR-769-5p |  |  |  |  | 1 |
| miR-675-5p | miR-944 |  |  |  |  | 0 |

Adjusted (age, sex, tumor stage, ASA-score, tumor differentiation) effects on differences

| **miRNA1** | **miRNA2** | **HR** | **HRlow** | **HRhigh** | **P** | **N** |
| --- | --- | --- | --- | --- | --- | --- |
| miR-34a-5p | miR-769-5p | 0.505 | 0.3158 | 0.8076 | 0.0043 | 51 |
| miR-125a-3p | miR-187 | 0.3708 | 0.1842 | 0.7467 | 0.0055 | 23 |
| miR-34a-5p | miR-187 | 0.4797 | 0.2821 | 0.8158 | 0.0067 | 24 |
| miR-148a | miR-187 | 0.5891 | 0.4001 | 0.8674 | 0.0074 | 24 |
| miR-29a-5p | miR-769-5p | 0.6529 | 0.4771 | 0.8935 | 0.0077 | 52 |
| miR-222-5p | miR-450b-5p | 2.1182 | 1.1772 | 3.8117 | 0.0123 | 25 |
| miR-187 | miR-769-5p | 2.0889 | 1.1553 | 3.7769 | 0.0148 | 24 |
| miR-29a-5p | miR-187 | 0.617 | 0.4175 | 0.912 | 0.0154 | 24 |
| miR-187 | miR-212-3p | 2.2339 | 1.1613 | 4.2969 | 0.016 | 24 |
| miR-146a-5p | miR-187 | 0.5432 | 0.3283 | 0.8987 | 0.0175 | 24 |
| miR-148a | miR-450b-5p | 2.1228 | 1.137 | 3.9634 | 0.0181 | 26 |
| miR-450b-5p | miR-769-5p | 0.3123 | 0.1159 | 0.8416 | 0.0214 | 26 |
| miR-34a-5p | miR-625-5p | 0.7088 | 0.5227 | 0.9611 | 0.0267 | 30 |
| miR-125a-3p | miR-769-5p | 0.7532 | 0.5846 | 0.9703 | 0.0283 | 49 |
| miR-29a-5p | miR-625-5p | 0.7417 | 0.557 | 0.9875 | 0.0408 | 30 |
| miR-205-5p | miR-222-5p | 0.8097 | 0.6596 | 0.994 | 0.0436 | 33 |
| let-7g | miR-187 | 0.6803 | 0.4624 | 1.0008 | 0.0505 | 24 |
| miR-205-5p | miR-769-5p | 0.8012 | 0.6416 | 1.0005 | 0.0506 | 35 |
| miR-146a-5p | miR-450b-5p | 1.7939 | 0.9892 | 3.2532 | 0.0543 | 26 |
| miR-212-3p | miR-450b-5p | 3.5008 | 0.9685 | 12.655 | 0.056 | 26 |
| miR-34a-5p | miR-148a | 0.7327 | 0.532 | 1.0092 | 0.0569 | 51 |
| miR-29a-5p | miR-222-5p | 0.8008 | 0.6341 | 1.0112 | 0.062 | 49 |
| miR-34a-5p | miR-146a-5p | 0.7096 | 0.4944 | 1.0186 | 0.0629 | 52 |
| miR-125a-3p | miR-450b-5p | 1.7573 | 0.9643 | 3.2027 | 0.0656 | 26 |
| miR-125a-3p | miR-222-5p | 0.7608 | 0.5569 | 1.0394 | 0.0859 | 46 |
| miR-205-5p | miR-625-5p | 0.7812 | 0.5887 | 1.0366 | 0.0871 | 24 |
| miR-125a-3p | miR-625-5p | 0.8195 | 0.6498 | 1.0337 | 0.0929 | 28 |
| miR-212-3p | miR-769-5p | 0.8065 | 0.6257 | 1.0397 | 0.0971 | 51 |
| miR-148a | miR-205-5p | 1.1697 | 0.9652 | 1.4176 | 0.1099 | 36 |
| miR-187 | miR-625-5p | 0.5438 | 0.253 | 1.169 | 0.1187 | 18 |
| miR-34a-5p | miR-222-5p | 0.8121 | 0.6237 | 1.0575 | 0.1224 | 48 |
| let-7g | miR-625-5p | 0.8015 | 0.605 | 1.0619 | 0.1232 | 30 |
| let-7g | miR-769-5p | 0.8318 | 0.6582 | 1.0513 | 0.1233 | 51 |
| miR-29a-5p | miR-450b-5p | 1.5252 | 0.8839 | 2.6319 | 0.1294 | 26 |
| miR-625-5p | miR-769-5p | 1.2558 | 0.9337 | 1.689 | 0.132 | 30 |
| let-7g | miR-34a-5p | 1.2639 | 0.929 | 1.7194 | 0.1359 | 51 |
| miR-212-3p | miR-625-5p | 0.8066 | 0.5976 | 1.0888 | 0.1603 | 30 |
| miR-187 | miR-222-5p | 1.3343 | 0.8856 | 2.0104 | 0.1679 | 24 |
| let-7g | miR-450b-5p | 1.4199 | 0.8606 | 2.3428 | 0.1699 | 26 |
| miR-34a-5p | miR-194-3p | 0.6026 | 0.2919 | 1.2443 | 0.171 | 23 |
| miR-194-3p | miR-205-5p | 1.6385 | 0.801 | 3.3515 | 0.1762 | 14 |
| miR-450b-5p | miR-625-5p | 0.7435 | 0.4831 | 1.1441 | 0.1777 | 19 |
| miR-146a-5p | miR-212-3p | 1.2652 | 0.8942 | 1.7902 | 0.1841 | 52 |
| miR-29a-5p | miR-194-3p | 0.6595 | 0.3524 | 1.2342 | 0.1929 | 24 |
| miR-146a-5p | miR-769-5p | 0.8347 | 0.6337 | 1.0995 | 0.1986 | 52 |
| miR-29a-5p | miR-205-5p | 1.0865 | 0.9502 | 1.2424 | 0.2253 | 36 |
| let-7g | miR-125a-3p | 1.2061 | 0.89 | 1.6345 | 0.2268 | 49 |
| let-7g | miR-222-5p | 0.8661 | 0.6775 | 1.1073 | 0.2516 | 49 |
| miR-148a | miR-769-5p | 0.9073 | 0.7647 | 1.0765 | 0.265 | 51 |
| miR-212-3p | miR-222-5p | 0.8536 | 0.6447 | 1.1302 | 0.2689 | 48 |
| miR-187 | miR-205-5p | 0.8217 | 0.5725 | 1.1794 | 0.2869 | 17 |
| miR-148a | miR-212-3p | 1.1319 | 0.9 | 1.4235 | 0.2896 | 51 |
| miR-194-3p | miR-212-3p | 1.2976 | 0.797 | 2.1128 | 0.2948 | 23 |
| miR-146a-5p | miR-205-5p | 1.0833 | 0.9272 | 1.2656 | 0.3136 | 36 |
| miR-146a-5p | miR-222-5p | 0.8997 | 0.7269 | 1.1137 | 0.3317 | 49 |
| let-7g | miR-205-5p | 1.0988 | 0.9043 | 1.335 | 0.3432 | 36 |
| miR-125a-3p | miR-146a-5p | 0.8932 | 0.7046 | 1.1324 | 0.351 | 50 |
| miR-125a-3p | miR-148a | 0.9204 | 0.7658 | 1.1062 | 0.3766 | 49 |
| let-7g | miR-212-3p | 1.1017 | 0.876 | 1.3855 | 0.4078 | 51 |
| miR-222-5p | miR-625-5p | 0.8548 | 0.5852 | 1.2485 | 0.4169 | 29 |
| miR-146a-5p | miR-625-5p | 0.8766 | 0.6244 | 1.2307 | 0.4468 | 30 |
| miR-34a-5p | miR-450b-5p | 1.4621 | 0.5434 | 3.9339 | 0.4519 | 26 |
| miR-29a-5p | miR-944 | *<*0.0001 | *<*0.0001 | *>*10000 | 0.4546 | 6 |
| miR-148a | miR-625-5p | 0.9012 | 0.6859 | 1.184 | 0.455 | 30 |
| miR-148a | miR-222-5p | 0.9478 | 0.8235 | 1.091 | 0.4555 | 49 |
| miR-34a-5p | miR-205-5p | 1.0782 | 0.8809 | 1.3197 | 0.4654 | 36 |
| miR-205-5p | miR-450b-5p | 0.8729 | 0.5971 | 1.276 | 0.4828 | 21 |
| let-7g | miR-146a-5p | 1.0833 | 0.865 | 1.3568 | 0.4857 | 52 |
| miR-146a-5p | miR-194-3p | 0.8927 | 0.6487 | 1.2286 | 0.4862 | 24 |
| miR-187 | miR-194-3p | 1.409 | 0.5335 | 3.7214 | 0.489 | 14 |
| let-7g | miR-148a | 1.0553 | 0.8859 | 1.2571 | 0.5467 | 52 |
| let-7g | miR-29a-5p | 1.0453 | 0.9042 | 1.2086 | 0.5491 | 52 |
| miR-29a-5p | miR-212-3p | 0.9445 | 0.7825 | 1.14 | 0.5519 | 52 |
| miR-205-5p | miR-212-3p | 0.95 | 0.8003 | 1.1278 | 0.558 | 36 |
| miR-148a | miR-944 | *>*10000 | *<*0.0001 | *>*10000 | 0.5695 | 6 |
| miR-125a-3p | miR-205-5p | 1.0599 | 0.8643 | 1.2998 | 0.5762 | 35 |
| miR-194-3p | miR-769-5p | 1.2043 | 0.6112 | 2.3729 | 0.5912 | 24 |
| miR-148a | miR-194-3p | 0.9487 | 0.7593 | 1.1853 | 0.6428 | 24 |
| miR-29a-5p | miR-125a-3p | 0.9596 | 0.8013 | 1.1493 | 0.6543 | 50 |
| miR-29a-5p | miR-34a-5p | 0.954 | 0.7729 | 1.1774 | 0.6609 | 52 |
| miR-194-3p | miR-450b-5p | 0.7149 | 0.159 | 3.2147 | 0.6618 | 13 |
| miR-222-5p | miR-769-5p | 0.9366 | 0.6923 | 1.2671 | 0.6709 | 49 |
| miR-34a-5p | miR-212-3p | 0.9148 | 0.6017 | 1.391 | 0.6772 | 52 |
| miR-34a-5p | miR-944 | *>*10000 | *<*0.0001 | *>*10000 | 0.7637 | 6 |
| miR-125a-3p | miR-194-3p | 1.0509 | 0.6595 | 1.6746 | 0.8346 | 21 |
| miR-205-5p | miR-431-5p | *>*10000 | *<*0.0001 | *>*10000 | 0.8697 | 6 |
| miR-194-3p | miR-625-5p | 0.8773 | 0.1419 | 5.4242 | 0.888 | 15 |
| miR-34a-5p | miR-125a-3p | 0.9833 | 0.7639 | 1.2656 | 0.8958 | 50 |
| miR-194-3p | miR-222-5p | 0.9785 | 0.5871 | 1.6309 | 0.9337 | 24 |
| miR-146a-5p | miR-148a | 1.0053 | 0.805 | 1.2556 | 0.9626 | 52 |
| miR-29a-5p | miR-146a-5p | 0.9975 | 0.8858 | 1.1232 | 0.9667 | 53 |
| miR-431-5p | miR-769-5p | *<*0.0001 | *<*0.0001 | *>*10000 | 0.9667 | 8 |
| let-7g | miR-194-3p | 0.9946 | 0.7136 | 1.3862 | 0.9746 | 24 |
| miR-29a-5p | miR-148a | 0.9988 | 0.906 | 1.1011 | 0.9809 | 52 |
| miR-125a-3p | miR-212-3p | 1.0011 | 0.8006 | 1.2517 | 0.9923 | 50 |
| miR-187 | miR-450b-5p | *>*10000 | *<*0.0001 | *>*10000 | 0.998 | 10 |
| let-7g | miR-944 | 4e-04 | *<*0.0001 | *>*10000 | 0.9997 | 6 |
| miR-146a-5p | miR-944 | *<*0.0001 | *<*0.0001 | *>*10000 | 0.9997 | 6 |
| miR-222-5p | miR-944 | 2e-04 | *<*0.0001 | *>*10000 | 0.9997 | 6 |
| let-7g | miR-431-5p | 0.1248 | *<*0.0001 | *>*10000 | 0.9999 | 8 |
| miR-29a-5p | miR-431-5p | *>*10000 | *<*0.0001 | *>*10000 | 0.9999 | 8 |
| miR-34a-5p | miR-431-5p | *<*0.0001 | *<*0.0001 | *>*10000 | 0.9999 | 8 |
| miR-125a-3p | miR-431-5p | 0.0237 | *<*0.0001 | *>*10000 | 0.9999 | 8 |
| miR-146a-5p | miR-431-5p | *>*10000 | *<*0.0001 | *>*10000 | 0.9999 | 8 |
| miR-148a | miR-431-5p | 96.1474 | *<*0.0001 | *>*10000 | 0.9999 | 8 |
| miR-212-3p | miR-431-5p | *<*0.0001 | *<*0.0001 | *>*10000 | 0.9999 | 8 |
| miR-222-5p | miR-431-5p | 0.0018 | *<*0.0001 | *>*10000 | 0.9999 | 8 |
| miR-125a-3p | miR-944 | 0.001 | *<*0.0001 | *>*10000 | 1 | 6 |
| miR-212-3p | miR-944 | *<*0.0001 | *<*0.0001 | *>*10000 | 1 | 6 |
| miR-431-5p | miR-450b-5p | *<*0.0001 | *<*0.0001 | *>*10000 | 1 | 6 |
| miR-769-5p | miR-944 | *<*0.0001 | *<*0.0001 | *>*10000 | 1 | 6 |
| let-7g | miR-675-5p |  |  |  |  | 1 |
| miR-29a-5p | miR-675-5p |  |  |  |  | 1 |
| miR-34a-5p | miR-675-5p |  |  |  |  | 1 |
| miR-125a-3p | miR-675-5p |  |  |  |  | 1 |
| miR-146a-5p | miR-675-5p |  |  |  |  | 1 |
| miR-148a | miR-675-5p |  |  |  |  | 1 |
| miR-187 | miR-431-5p |  |  |  |  | 3 |
| miR-187 | miR-675-5p |  |  |  |  | 0 |
| miR-187 | miR-944 |  |  |  |  | 3 |
| miR-194-3p | miR-431-5p |  |  |  |  | 4 |
| miR-194-3p | miR-675-5p |  |  |  |  | 0 |
| miR-194-3p | miR-944 |  |  |  |  | 4 |
| miR-205-5p | miR-675-5p |  |  |  |  | 1 |
| miR-205-5p | miR-944 |  |  |  |  | 4 |
| miR-212-3p | miR-675-5p |  |  |  |  | 1 |
| miR-222-5p | miR-675-5p |  |  |  |  | 1 |
| miR-431-5p | miR-625-5p |  |  |  |  | 3 |
| miR-431-5p | miR-675-5p |  |  |  |  | 0 |
| miR-431-5p | miR-944 |  |  |  |  | 2 |
| miR-450b-5p | miR-675-5p |  |  |  |  | 1 |
| miR-450b-5p | miR-944 |  |  |  |  | 4 |
| miR-625-5p | miR-675-5p |  |  |  |  | 1 |
| miR-625-5p | miR-944 |  |  |  |  | 4 |
| miR-675-5p | miR-769-5p |  |  |  |  | 1 |
| miR-675-5p | miR-944 |  |  |  |  | 0 |
